# Dysregulation of the miR‐30c/DLL4 axis by circHIPK3 is essential for KSHV lytic replication

**DOI:** 10.15252/embr.202154117

**Published:** 2022-03-03

**Authors:** Katherine L Harper, Timothy J Mottram, Chinedu A Anene, Becky Foster, Molly R Patterson, Euan McDonnell, Andrew Macdonald, David Westhead, Adrian Whitehouse

**Affiliations:** ^1^ School of Molecular and Cellular Biology and Astbury Centre for Structural Molecular Biology University of Leeds Leeds UK; ^2^ Centre for Cancer Genomics and Computational Biology Barts Cancer Institute Queen Mary University of London London UK; ^3^ Department of Biochemistry and Microbiology Rhodes University Grahamstown South Africa

**Keywords:** circRNAs, Kaposi's sarcoma (KS)‐associated herpesvirus, ncRNAs, regulatory network, Microbiology, Virology & Host Pathogen Interaction, RNA Biology, Signal Transduction

## Abstract

Non‐coding RNA (ncRNA) regulatory networks are emerging as critical regulators of gene expression. These intricate networks of ncRNA:ncRNA interactions modulate multiple cellular pathways and impact the development and progression of multiple diseases. Herpesviruses, including Kaposi’s sarcoma‐associated herpesvirus, are adept at utilising ncRNAs, encoding their own as well as dysregulating host ncRNAs to modulate virus gene expression and the host response to infection. Research has mainly focused on unidirectional ncRNA‐mediated regulation of target protein‐coding transcripts; however, we identify a novel host ncRNA regulatory network essential for KSHV lytic replication in B cells. KSHV‐mediated upregulation of the host cell circRNA, circHIPK3, is a key component of this network, functioning as a competing endogenous RNA of miR‐30c, leading to increased levels of the miR‐30c target, DLL4. Dysregulation of this network highlights a novel mechanism of cell cycle control during KSHV lytic replication in B cells. Importantly, disruption at any point within this novel ncRNA regulatory network has a detrimental effect on KSHV lytic replication, highlighting the essential nature of this network and potential for therapeutic intervention.

## Graphical abstract

Lytic replication of Kaposi’s sarcoma‐associated herpesvirus in B cells depends on the dysregulation of a host non‐coding RNA regulatory network. The circular RNA circHIPK3 interferes with the miR‐30c/DLL4 axis to enhance viral lytic replication.

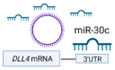

## Introduction

Within the huge diversity of RNA species transcribed from the human genome, the majority have no coding capacity and are instead designated non‐coding RNAs (ncRNAs). Even within ncRNAs, there is a wide repertoire of species generally categorised based on their size. Small ncRNAs are less than 200 nucleotides in length, and include microRNAs (miRNAs), small‐interfering, small nuclear, small nucleolar and PIWI‐interacting RNAs (Heyns & Kovalchuk, 2015). In contrast, long ncRNAs (lncRNAs) vary in length from 200 nucleotides to 100 kilobases, and include long intergenic, long intronic, sense and antisense RNAs (Tsagakis *et al*, 2020). As interest in different ncRNAs species has increased, so too have their known assigned functions. It is now understood that far being from errors in transcription, ncRNAs have key roles in regulating all aspects of cell biology and are critical regulators of gene expression. This is reinforced by ncRNA dysregulation being implicated in the development and progression of many disease states, including cancer, neurological disorders and infection (Schmitt & Chang, 2016; Li *et al*, 2018a; Qiu *et al*, 2018).

To date, research has mainly focused on unidirectional ncRNA‐mediated regulation of target protein‐coding transcripts, exemplified by miRNA‐mediated regulation of mRNA targets (O'Brien *et al*, 2018). However, there is emerging evidence of the existence of interplay between ncRNAs that strongly influence how gene expression is regulated by forming ncRNA regulatory networks. A cornerstone of these networks is the ability to act as competing endogenous RNA (ceRNAs), here ncRNAs can compete with each other for mRNA binding, thereby adding a new layer of regulatory potential (Kartha & Subramanian, 2014; Zhong *et al*, 2018). Several mechanistic interactions allow ncRNAs to function as ceRNA regulators including; miRNAs interacting with lncRNAs to reduce their stability, ncRNAs competing with miRNAs for the interaction with shared mRNA targets and ncRNAs acting as miRNA sponges or decoys to enhance target mRNAs expression (Ulitsky, 2018; Yamamura *et al*, 2018).

Circular RNAs (circRNAs) are a novel class of lncRNAs, characterised by a covalently closed loop lacking a 5' end cap or 3' poly (A) tail. Originally thought to be rare splicing errors, recent breakthroughs have shown circRNAs are highly abundant and play key roles in gene regulation (Taylor, 2014; Qu *et al*, 2018). circRNAs are mostly formed through a unique backsplicing mechanism, where a downstream splice donor is joined to an upstream splice acceptor forming a closed circle (Bolha *et al*, 2017). These structures are highly stable, conserved across many species and often cell type or condition specific (Holdt *et al*, 2018). Implicated in a wide range of functions, including protein interactions and transcriptional regulators, interest in circRNAs was ignited with the emergence of their role as ceRNAs through miRNA interactions. In 2008, ciRS‐7 was shown to sponge miR‐7, acting as a key ncRNA regulator, since then numerous examples of functional ceRNA circRNAs have emerged, strongly implicated circRNAs as key regulators (Hansen *et al*, 2013).

The ability of ncRNAs to act as regulators of gene expression offers the potential for viruses to subvert these processes aiding in their own replication or to modulate the host cell response to infection. Herpesviruses, such as Kaposi’s sarcoma‐associated herpesvirus (KSHV), are particularly proficient at utilising ncRNA regulatory pathways (Wang *et al*, 2018). KSHV‐encoded miRNAs and lncRNAs, as well as dysregulating host cell miRNAs help in the establishment of a persistent latent infection, evading the immune response and regulating the switch between latent and lytic replication cycles (Catrina Ene *et al*, 2014; Qin *et al*, 2017). Recent evidence has also shown that KSHV encodes its own circRNAs, with a circ‐vIRF4 identified by multiple studies (Toptan *et al*, 2018; Abere *et al*, 2020). Hundreds of highly variable low copy number circRNAs are also expressed from the PAN locus, although functionality has not yet been established. Another circRNA derived from K12 gene contained the miRNA sequences also expressed from this gene; it has therefore been hypothesised that it could act as ceRNA of its own miRNAs (Tagawa *et al*, 2021). This suggests viruses may dysregulate cellular circRNAs as part of complex ncRNA regulatory networks, aiding in viral manipulation of the host cell.

Herein, we have identified that KSHV dysregulates a novel host cell ncRNA regulatory network which is essential for successful viral lytic replication and infectious virion production in B cells. CircHIPK3 is a key component of this network and we have demonstrated that circHIPK3 acts as a ceRNA, sponging miR‐30c leading to an increase in the levels of the miR‐30c target, DLL4. We further demonstrate that disruption at any point within this novel network has a detrimental effect on KSHV lytic replication, highlighting the essential nature of this network.

## Results

### miR‐29b and miR‐30c are inhibitory miRNAs dysregulated during KSHV lytic replication

**Figure 1:**
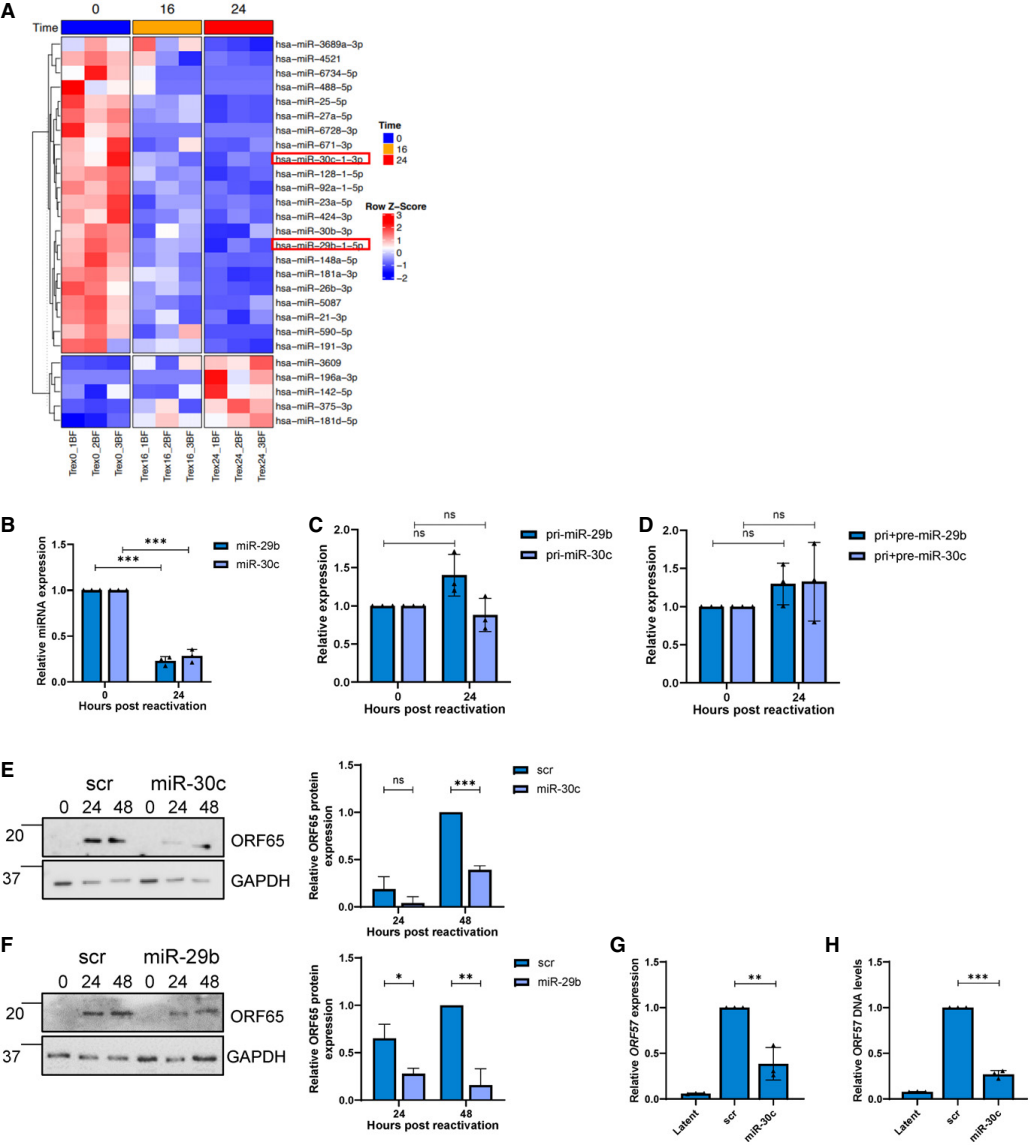
KSHV dysregulates inhibitory miRNA AHeat map of differentially expressed miRNAs during KSHV replication at 0, 16 and 24 h identified through miR‐Seq. miR‐29b and miR‐30c are highlighted in red, with *T* test analysis.BqPCR analysis of miR‐29b and miR‐30c levels in cells at 0 and 24 h post‐lytic induction. SNORD68 was used as a housekeeper (*n* = 3).C, DqPCR analysis of TREx 0 and 24 post‐induction (*n* = 3) using GAPDH as a housekeeper, and analysis is of levels of pri‐miR‐29b/pri‐miR‐30c (C) and pri+pre‐miR‐29b/pri+pre‐miR‐30c (D).ERepresentative western blot of scr or miR‐30c transfected TREx cells analysed for ORF65 expression with GAPDH as a loading control. Densitometry analysis performed on *n* = 3.FRepresentative western blot of scr or miR‐29b transfected TREx cells analysed for ORF65 expression with GAPDH as a loading control. Densitometry analysis performed on *n* = 3.GqPCR analysis of ORF57 DNA levels for viral load at 72 h post induction, with scr and miR‐30c transfected TREx cells with uninduced cells as a control and GAPDH as a housekeeper (*n* = 3).HqPCR analysis of *ORF57* in HEK‐293T cells for reinfection assay. Data information: In (B–H), data are presented as mean ± SD. **P < 0.01 and ***P < 0.001 (unpaired Student’s *t*‐test). All repeats are biological. Source data are available online for this figure.

To determine whether KSHV infection dysregulates specific host cell ncRNA networks, we first assessed which cellular miRNAs were dysregulated during KSHV reactivation in B cells, as miRNAs are central in ncRNA axes. miR‐Seq (Yeri *et al*, 2018) was utilised to identify miRNAs which are altered during the course of KSHV lytic replication at 0, 16 and 24 h post‐reactivation in TREx‐BCBL1‐RTA cells, a KSHV‐latently infected B‐lymphocyte cell line containing a Myc‐tagged version of the viral RTA under the control of a doxycycline‐inducible promoter. Z‐score analysis of the miR‐Seq highlighted the most consistently dysregulated miRNAs, with the vast majority downregulated during lytic replication in B cells (Fig 1A and Appendix Fig S1A). Several of the most dysregulated miRNAs were selected for further investigation.

miR‐Seq results were further supported by available microarray online datasets showing two of the candidates: miR‐29b and miR‐30c are downregulated in KSHV‐positive B cell lines (Data ref: Wu *et al*, 2015a) (Appendix Fig S1B and C) (Wu *et al*, 2015a; Wu *et al*, 2015b). In addition, the miR‐30 family has been previously observed to be downregulated upon KSHV infection of lymphatic endothelial cells (Bridge *et al*, 2012). Other miRNAs highlighted in our miR‐Seq were not significantly changed in online microarray datasets, further prioritising selection of miR‐29b and miR‐30c for further investigation (Appendix Fig S1D–F). To conclusively confirm downregulation of miR‐29b and miR‐30c in TREx‐BCBL1‐RTA cells, qPCR analysis was utilised to determine mature miRNA levels. Results confirmed a dramatic reduction in mature miR‐29b and miR‐30c levels at 24 h post‐reactivation, 75 and 70% respectively (Fig 1B). Levels of a miRNA that was unchanged during lytic replication were also confirmed via qPCR to confirm validity of the assay (Appendix Fig S1G). Notably, quantification of the primary and pre‐miRNAs levels via qPCR showed no significant decrease, implicating downregulation occurs at the miRNA mature level and hypothesised this downregulation could be occurring through a ncRNA network (Fig 1C and D).

Due to their consistent downregulation, we speculated that miR‐29b and miR‐30c may have an inhibitory effect on KSHV lytic replication in B cells. miRNA mimics were therefore transfected separately into TREx‐BCBL1‐RTA cells resulting in miRNA overexpression (Appendix Fig S1H). Reactivation assays demonstrated that miR‐29b and miR‐30c overexpression resulted in a significant reduction in the lytically expressed late ORF65 protein, compared to a scrambled control (Fig 1E and F). Further experiments focused on miR‐30c due to its more consistent effect on virus replication. Additionally, viral genomic DNA was measured via qPCR from scrambled and miR‐30c overexpressing TREx‐BCBL1‐RTA cells to assess whether viral DNA load was affected, with cells overexpressing the miRNA having a 75% reduction (Fig 1G). To examine whether miR‐30c overexpression also affected infectious virion production, supernatants of reactivated scrambled and miRNA overexpressing TREx‐BCBL1‐RTA cells were used to reinfect naive HEK‐293T cells and qPCR used to determine KSHV ORF57 expression. Cells reinfected with supernatant from miRNA‐overexpressing cells contained 60% less viral RNA compared to controls (Fig 1H). A miR‐30c antagomiR was also transfected into TREx‐BCBL1‐RTA cells, upon KSHV reactivation, this led to a small further reductions in miR‐30c levels (Appendix Fig S1I) and a non‐significant increase in viral ORF65 levels, likely due to the majority of miR‐30c already reduced during lytic replication (Appendix Fig S1J). Taken together these data suggest that KSHV lytic replication in B cells is impacted by the overexpression of miR‐29b and miR‐30c, suggesting that they are downregulated during KSHV lytic replication due to their inhibitory effect.

### CircHIPK3 sponges miR‐29b and miR‐30c

**Figure 2:**
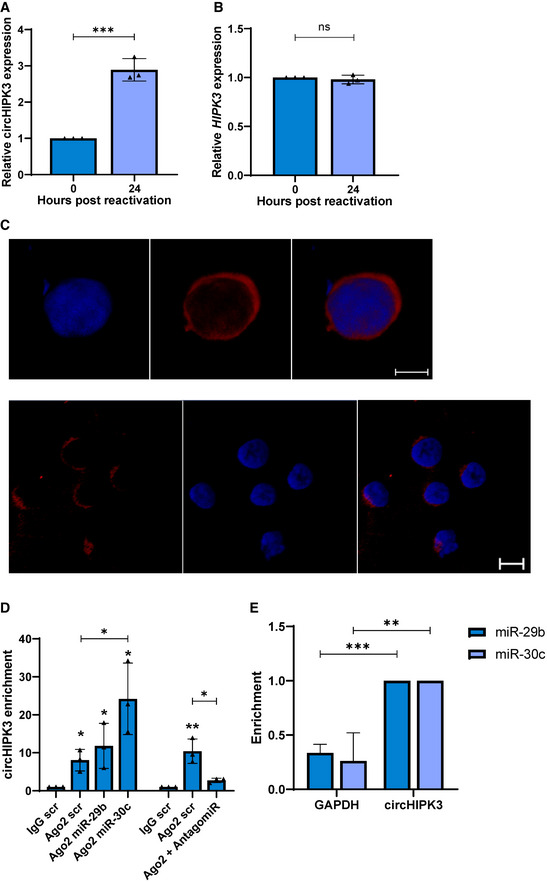
CircHIPK3 sponges miR‐29b and miR‐30c A, BqPCR analysis of TREx 0 and 24 post‐induction (*n* = 3) using GAPDH as a housekeeper, analysis is of levels of circHIPK3 (A) and *HIPK3* (B).CFISH analysis of TREx cells reactivated for 16 h with probes against circHIPK3 (red). DAPI was used as nuclei stain (blue), scale bars represent 5 μm on upper panel and 10 μm on lower panel. The lower panel shows a wider field of view.DqPCR analysis of Ago2 RIPs in TREx cells transfected with scr, miR‐29b or miR‐30c showing circHIPK3 enrichment over GAPDH, with each repeat normalised to an IgG scr RIP (*n* = 3).EqPCR analysis of Streptavidin RIPs of circHIPK3 or GAPDH enrichment in HEK‐293T cells transfected with either biotinylated miR‐29b or miR‐30c over scr (*n* = 4). Data information: In (A, B, D and E) data are presented as mean ± SD. *P < 0.05, **P < 0.01 and ***P < 0.001 (unpaired Student’s *t*‐test). All repeats are biological. Source data are available online for this figure.

We next determined whether KSHV could dysregulate these inhibitory miRNAs through a ncRNA network. CircRNAs, have previously been reported to act as miRNA sponges thereby regulating miRNA/mRNA axes. Notably, RNA‐binding prediction software (Freiburg RNA tools, IntaRNA–RNA–RNA interactions and University of Bielefeld BiBiServ RNAhybrid program) highlighted circHIPK3 (hsa_circ_0000284) contained complementary sequences to both miR‐29b and miR‐30c, with both software identifying two high score binding sites for miR‐29b, and two and three, respectively, for miR‐30c (Appendix Fig S2A). To assess whether circHIPK3 levels were altered during KSHV lytic replication, qPCR was performed using divergent primers annealing at the distal ends of circHIPK3 (Appendix Fig S2B). Results showed a ~ 3‐fold increase in circHIPK3 at 24 h post‐reactivation compared to latent samples (Fig 2A). Notably, this upregulation was specific to the circular transcript, as the mRNA levels of *HIPK3* showed no significant change (Fig 2B). We next assessed the subcellular localisation of circHIPK3 by fluorescent *in situ* hybridisation (FISH), using specific probes against the unique backsplice site within circHIPK3. Results demonstrated a clear cytoplasmic localisation in TREx‐BCBL1‐RTA cells (Fig 2C). To further support a potential sponging activity of circHIPK3, Ago2 RNA immunoprecipitations (RIPs) were performed to determine whether circHIPK3 associates with the miRNA machinery. CircHIPK3 levels were normalised to GAPDH in each respective RIP to account for background, with each enrichment then compared to the scrambled transfected IgG control. Results show a greater fold enrichment of circHIPK3 over GAPDH in the Ago2 RIPs. Moreover, overexpression of either miR‐29b or miR‐30c mimics further enhanced this enrichment in the Ago2 RIP. In contrast, transfection of a miR‐30c antagomiR reduced enrichment (Fig 2D).

Finally, to confirm direct binding between circHIPK3 and miR‐29b or miR‐30c, biotinylated miR‐29b or miR‐30c was transfected into HEK‐293Ts and a RIP was performed using Streptavidin beads. Results showed a significant enrichment of circHIPK3 in the miRNA transfected samples compared to scrambled controls (Fig 2E). Together these data suggest that circHIPK3 is upregulated during KSHV lytic replication and functions as a molecular sponge for miR‐29b and miR‐30c.

### CircHIPK3 is essential for KSHV replication

**Figure 3:**
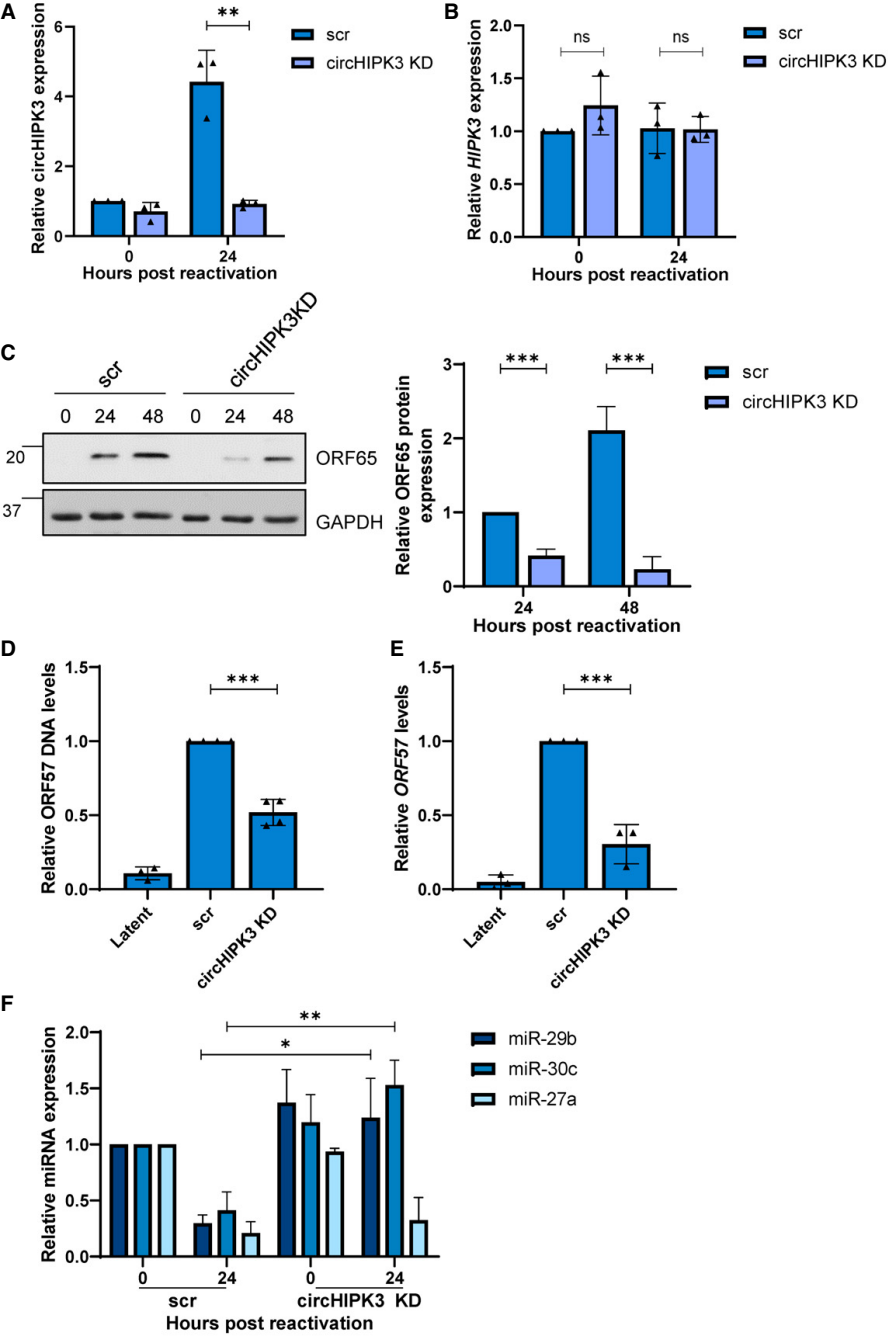
CircHIPK3 is essential for KSHV replication qPCR analysis of circHIPK3 levels in scr and circHIPK3 KD stably expressing TREx cells. GAPDH was used as a housekeeper (*n* = 3).qPCR analysis of *HIPK3* levels in scr and circHIPK3 KD stably expressing TREx cells. GAPDH was used as a housekeeper (*n* = 3).Representative western blot of ORF65 levels in scr and circHIPK3 KD cell lines with GAPDH as a loading control. Densitometry analysis of *n* = 3 western blots.qPCR analysis of ORF57 DNA levels for viral load at 72 h post‐induction, with scr and circHIPK3 KD TREx cells with uninduced cells as a control and GAPDH as a housekeeper (*n* = 3).qPCR analysis of *ORF57* in HEK‐293T cells for reinfection assay (*n* = 3).qPCR analysis of levels of miR‐29b, miR‐30c and miR‐27a in scr and circHIPK3 KD cells 0 and 24 h post‐induction. SNORD68 was used as a housekeeper (*n* = 3). Data information: In (A–F), data are presented as mean ± SD. *P < 0.05, **P < 0.01 and ***P < 0.001 (unpaired Student’s *t*‐test). All repeats are biological. Source data are available online for this figure.

To assess the importance of circHIPK3 on KSHV lytic replication, TREx‐BCBL1‐RTA cells were transduced with lentivirus‐based shRNAs targeting the unique backsplice of circHIPK3. qPCR confirmed successful depletion of circHIPK3, but importantly knockdown had little effect on the *HIPK3* transcript (Fig 3A and B), meaning any effects of the knockdown was solely due to circHIPK3 depletion. Reactivation assays demonstrated that circHIPK3 depletion resulted in a significant reduction in ORF65 protein production compared to the scrambled control (Fig 3C). The effect of circHIPK3 knockdown was further evaluated using viral DNA load and reinfection assays, with a significant decrease in both viral load and infectious virion production being observed upon depletion (Fig 3D and E). Together this confirms that KSHV specifically requires the function of circHIPK3 to undergo efficient lytic replication and infectious virion production. With the importance of circHIPK3 of KSHV lytic replication confirmed, we next assessed what effect circHIPK3 depletion had upon levels of miR‐29b or miR‐30c during lytic replication. As previously observed, both miR‐29b and miR‐30c were downregulated in scrambled control cells at 24 h post‐reactivation, in contrast no significant downregulation was observed in miR‐29b and miR‐30c levels upon circHIPK3 depletion (Fig 3F). This effect was specific to miR‐29b and miR‐30c as circHIPK3 depletion had no effect on miR‐27a levels, another miRNA downregulated upon KSHV lytic replication (Fig 3F). Together this shows that KSHV‐mediated circHIPK3 dysregulation can specifically affect miR‐29b and miR‐30c levels.

### KSHV ORF57 expression enhances circHIPK3 levels

**Figure 4:**
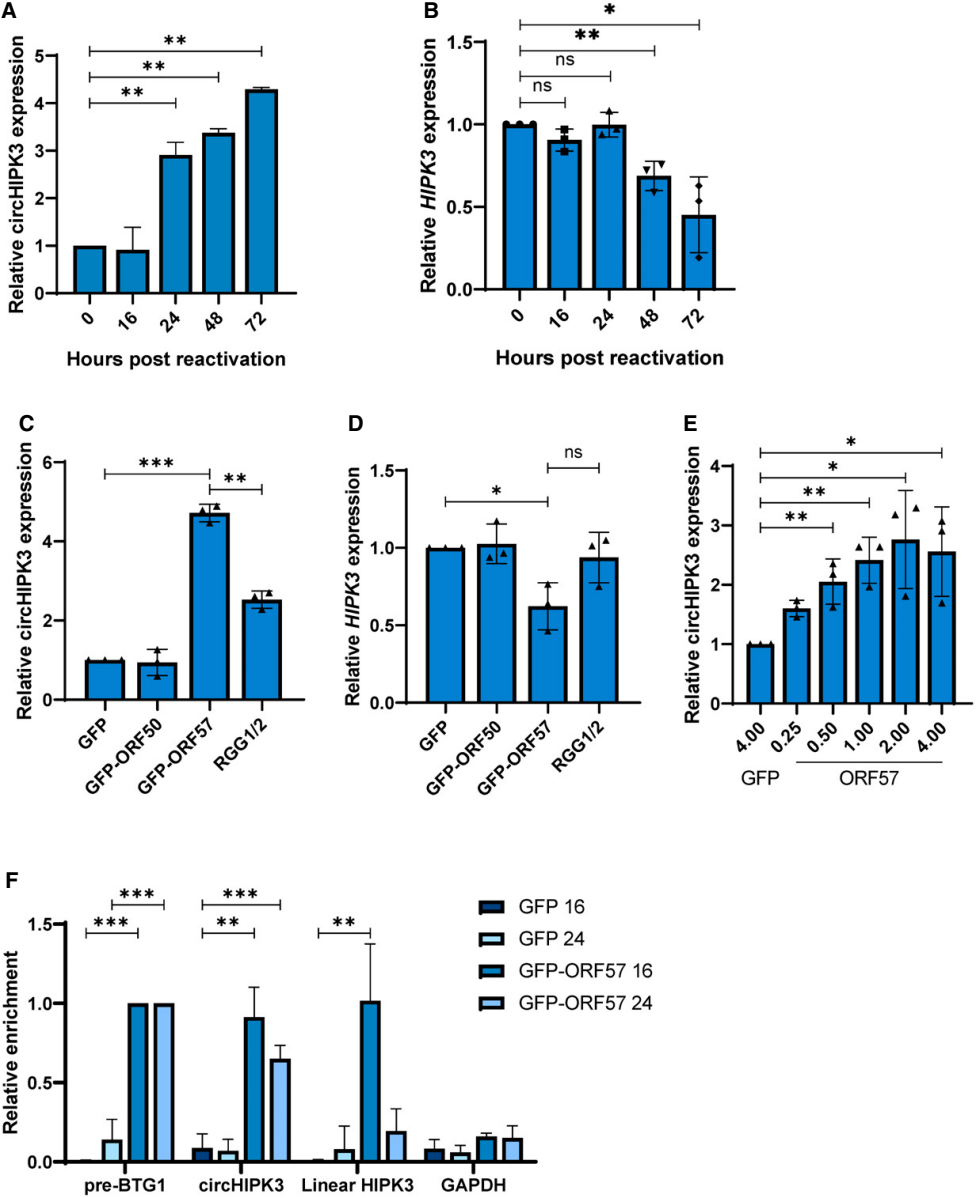
ORF57 contributes to the upregulation of circHIPK3 levels qPCR analysis of circHIPK3 levels at time points in TREx post‐induction with GAPDH as a housekeeper (*n* = 3).qPCR analysis of *HIPK3* levels at time points in TREx post‐induction with GAPDH as a housekeeper (*n* = 3).qPCR analysis of circHIPK3 expression in HEK‐293Ts transfected with GFP, ORF50‐GFP, ORF57‐GFP or ORF‐57‐GFP RGG1/2 with GAPDH as a housekeeper (*n* = 3).qPCR analysis of *HIPK3* expression in HEK‐293Ts transfected with GFP, ORF50‐GFP, ORF57‐GFP or ORF‐57‐GFP RGG1/2 with GAPDH as a housekeeper (*n* = 3).qPCR analysis of circHIPK3 expression in HEK‐293Ts transfected with 4 µg GFP, or 0.25–4 µg ORF57‐GFP, GAPDH was used as a housekeeper (*n* = 3).qPCR analysis GFP RIPs in GFP or ORF57‐GFP transfected HEK‐293Ts at 16 and 24 h post‐transfection, respectively, *n* = 3 for each time course. Data information: In (A–F), data are presented as mean ± SD. *P < 0.05, **P < 0.01 and ***P < 0.001 (unpaired Student’s *t*‐test). All repeats are biological. Source data are available online for this figure.

To determine if any KSHV‐encoded proteins are involved in dysregulating circHIPK3 levels, we firstly assessed circHIPK3 levels at different time points post‐reactivation. qPCR showed a clear upregulation between 16 and 24 h (Fig 4A), linear *HIPK3* levels in contrast were stable until 24 h before a rapid drop‐off at 72 h post‐lytic induction (Fig 4B). We therefore examined whether any early KSHV proteins were sufficient to induce circHIPK3 levels independently. HEK‐293T cells were transfected with control GFP, KSHV ORF50‐GFP or ORF57‐GFP expression constructs (Gould *et al*, 2009; Schumann *et al*, 2016) and circHIPK3 levels assessed by qPCR at 24 h post‐transfection. Results showed expression of ORF57‐GFP alone was sufficient to increase circHIPK3 levels (Fig 4C) and this upregulation was dose dependent (Fig 4E), interestingly transfection with GFP‐ORF57 led to a small decrease in linear *HIPK3* levels (Fig 4D). Western blotting confirmed successful transfections (Appendix Fig S3A and B).

The role of ORF57 was further investigated with previously characterised RNA‐binding and dimerisation‐negative mutants (RGG1/2 and W292A respectively) (Gould *et al*, 2009; Schumann *et al*, 2016), with the RNA‐binding mutant being unable to bind to circHIPK3 (Appendix Fig S3C); however, the dimerisation mutant was too unstable to utilise (Appendix Fig S3D). CircHIPK3 levels in HEK‐293Ts were lower when transfected with the RNA‐binding mutant compared to wild‐type ORF57, however, an upregulation was still observed over GFP and GFP‐ORF50 (Fig 4C). Additionally, the observed drop in linear *HIPK3* levels when transfected with GFP‐ORF57 did not occur with the RGG1/2 mutant. This is indicative that ORF57 RNA‐binding domains play a role in the observed increased levels of circHIPK3 (Fig 4D), although the exact mechanism by which ORF57 increases circHIPK3 levels is yet to be fully elucidated.

KSHV ORF57 is a multifunctional RNA‐binding protein involved in several stages of viral RNA processing (Schumann *et al*, 2013; Majerciak & Zheng, 2015). Therefore, we assessed whether ORF57 interacted with circHIPK3 using RNA immunoprecipitations performed in control or ORF57‐GFP‐transfected HEK‐293T cells utilising GFP‐TRAP beads. RIPs were performed at 16 and 24 h post‐transfection and results showed that ORF57 associated with both circHIPK3 and the linear HIPK3 transcript strongly at 16 h. In contrast, at 24 h, ORF57 showed stronger association with circHIPK3 compared to the linear form (Fig 4F). This suggests that ORF57 binds both the linear and circular forms of HIPK3 with the ratio of binding changing over time.

Together these data suggest that ORF57 may be involved in the increase in circHIPK3 levels during KSHV lytic replication, however, whether ORF57 promotes increased biogenesis or is involved in other aspects, such as promoting circRNA stability, is, as yet, unknown.

### DLL4 is regulated by miR‐30c and circHIPK3

**Figure 5:**
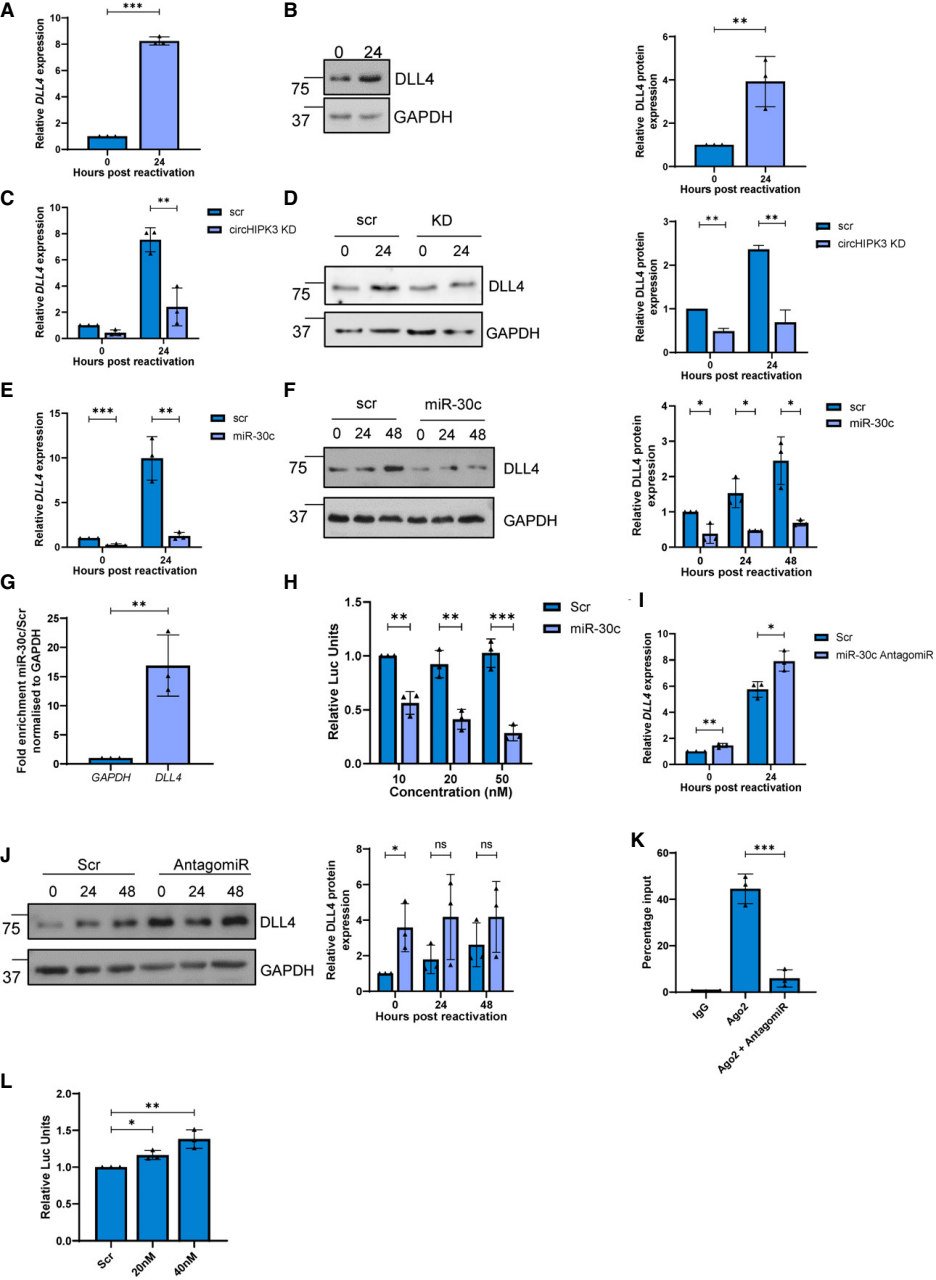
DLL4 is targeted by circHIPK3 and miR‐30c qPCR analysis of *DLL4* levels in TREx cells 0 and 24 h post‐induction. GAPDH was used as a housekeeper (*n* = 3).Representative western blot of DLL4 expression in TREx cells 0 and 24 h post‐induction. GAPDH was used a loading control (*n* = 3).qPCR of *DLL4* levels in scr and circHIPK3 KD stable TREx cells at 0 and 24 h post‐induction with GAPDH as a housekeeper (*n* = 3).Representative western blot of DLL4 levels in scr and circHIPK3 stable TREx cells at 0 and 24 h post‐induction with GAPDH as a loading control. Densitometry analysis of *n* = 3.qPCR analysis of *DLL4* levels in TREx cells that have been transfected with either scr or miR‐30c 24 h pre‐induction, analysis performed 0 and 24 h post‐induction. GAPDH was used as a housekeeper (*n* = 3).Representative western blot of DLL4 levels in scr‐ or miR‐30c‐transfected TREx cells at 0, 24 and 48 h post induction with GAPDH as a loading control. Densitometry analysis of *n* = 3Fold enrichment of *GAPDH* and *DLL4* in biotinylated miR‐30c RIPs over Scr transfected into HEK‐293Ts (*n* = 3)Luciferase reporter assay from HEK‐293Ts co‐transfected with DLL4 3’UTR reporter plasmid and either scr or miR‐30c. Data presented are relative to an internal firefly control (*n* = 3).qPCR analysis of *DLL4* levels in TREx cells that have transfected with either scr or a miR‐30c antagomiR 24 h pre‐induction, with analysis performed at 0 and 24 h post‐induction. GAPDH was used as a housekeeper (*n* = 3).Representative western blot of DLL4 levels in scr or miR‐30c antagomiR‐transfected TREx cells at 0, 24 and 48 h post‐induction with GAPDH as a loading control. Densitometry analysis of *n* = 3.qPCR analysis of *DLL4* expression in Ago2 RIPs as a percentage of input. TREx cells were transfected with either a scrambled control or a miR‐30c antagomiR (*n* = 3).Luciferase reporter assay from HEK‐293Ts co‐transfected with DLL4 3’UTR reporter plasmid and either scr or miR‐30c antagomiR. Data presented are relative to an internal firefly control (*n* = 3). Data information: In (A–L), data are presented as mean ± SD. *P < 0.05, **P < 0.01 and ***P < 0.001 (unpaired Student’s *t*‐test). All repeats are biological. Source data are available online for this figure.

The dependence of the circHIPK3/miR‐30c axis to enhance KSHV lytic replication in B cells suggests that KSHV may modulate this ncRNA axis to regulate expression of miR‐30c‐specific mRNA targets. Previous results have shown that the miR‐30 family targets DLL4 (Bridge *et al*, 2012) and combined cross referencing of online databases with upregulated mRNAs from RNA‐Seq during KSHV infection (Appendix Fig S4A), qPCR and immunoblotting confirmed DLL4 was upregulated at both the RNA and protein level during KSHV lytic replication in TREx‐BCBL1‐RTA cells (Fig 5A and B). Moreover, we found that other mRNA targets of miR‐30c were significantly overexpressed during KSHV lytic replication (Appendix Fig S4B), further suggesting miR‐30c plays a role in KSHV lytic replication.

To determine whether the circHIPK3/miR‐30c axis specifically affected DLL4 expression, we assessed what effect either circHIPK3 depletion or overexpression of a miR‐30c mimic had on DLL4 expression in TREx‐BCBL1‐RTA cells. In both cases, we observed a significant reduction in DLL4 mRNA and protein levels, implying both circHIPK3 and miR‐30c regulate DLL4 levels (Fig 5C–F). These results were further supported by analysis of *DLL4* levels in miR‐30c RIPs, where biotinylated miR‐30c or a scrambled control was transfected into HEK‐293Ts prior to a RIP being performed using Streptavidin beads. *DLL4* showed significant enrichment in the miR‐30c RIP over scrambled controls, indicating it directly associates with miR‐30c (Fig 5G). A 3’ UTR luciferase assay was also utilised, where the 3’ UTR of *DLL4* was cloned into a luciferase reporter plasmid. Transfection of miR‐30c leads to a reduction in luminescence, which confirmed miR‐30c directly regulates *DLL4* expression in TREx‐BCBL1‐RTA cells (Fig 5H).

Transfection of a miR‐30c antagomiR also resulted in small increases in DLL4 at both RNA and protein levels (Fig 5I and J). Ago2 RIPs were then performed in the absence or presence of the antagomiR. *DLL4* had strong enrichment in Ago2 RIPs, however, transfection of the antagomiR led to a reduced association. This indicates that when miR‐30c levels are reduced, *DLL4* as a putative target associates less with the miRNA machinery (Fig 5K). Finally, the 3’ UTR luciferase assay was performed in the presence of the antagomiR, which led to a slight increase in luminescence (Fig 5L), reinforcing that DLL4 is a target of miR‐30c.

### DLL4 is essential for KSHV lytic replication in B cells

**Figure 6:**
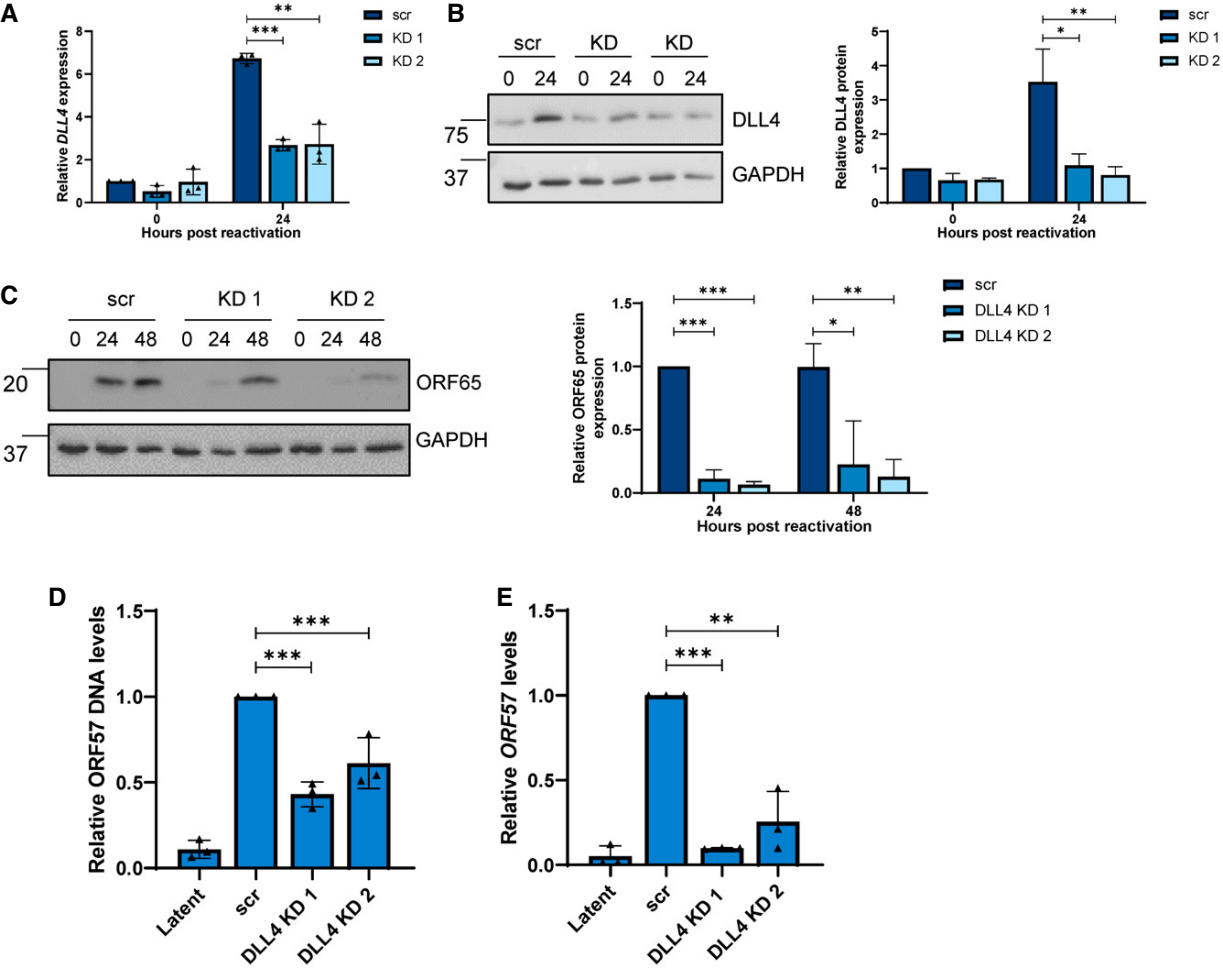
CircHIPK3:miR‐30c:DLL4 dysregulation affects cell cycle qPCR of *DLL4* expression in stable expression scr and two *DLL4* KD TREx cell lines at 0 and 24 h post‐induction. GAPDH was used as a housekeeper (*n* = 3).Representative western blot of DLL4 levels in scr and DLL4 KD TREx cells with GAPDH as a loading control, densitometry analysis is *n* = 3.Representative western blot of ORF65 levels at 0, 24 and 48 h post‐induction in scr and DLL4 KD TREx cells, GAPDH was used as a loading control and densitometry analysis performed on *n* = 3.qPCR analysis of ORF57 DNA levels for viral load at 72 h post‐induction, with scr and DLL4 KD cells with uninduced cells as a control and GAPDH as a housekeeper (*n* = 3).qPCR analysis of *ORF57* in HEK‐293T cells for reinfection assay (*n* = 3). Data information: In (A–E), data are presented as mean ± SD. *P < 0.05, **P < 0.01 and ***P < 0.001 (unpaired Student’s *t*‐test). All repeats are biological. Source data are available online for this figure.

To confirm the importance of DLL4 during KSHV lytic replication, TREx‐BCBL1‐RTA cells were transduced with lentivirus‐based shRNAs, significantly depleting DLL4 RNA and protein expression during lytic replication (Fig 6A and B). Reactivation assays demonstrated that DLL4 depletion resulted in a significant reduction in late ORF65 protein expression compared to the scrambled control (Fig 6C). Similarly, DLL4 depletion resulted in a significant reduction in viral load and infectious virion production, compared to scrambled control (Fig 6D and E). Together, these data highlight that dysregulation of a circHIPK3/miR‐30c/DLL4 regulatory circuit is essential for efficient KSHV lytic replication. Experiments were also performed to investigate overall levels of circHIPK3, using a standard curve qPCR method. To successfully act in the axis, circHIPK3 must be highly expressed with estimates in TREx‐BCBL‐1‐Rta cells showing circHIPK3 had the highest expression of the three components of the axis (Appendix Fig S5A–C).

The role of circHIPK3, miR‐30c and *DLL4* was also investigated via qPCR in an additional KSHV‐infected cell line, namely the adherent cell line HEK‐293T‐rKSHV.219 (Appendix Fig S5D–F). Here, circHIPK3 and *DLL4* were upregulated upon lytic reactivation, while miR‐30c was downregulated. Finally, a second KSHV‐infected B cell line was utilised, with levels of circHIPK3, miR‐30c and *DLL4* measured via qPCR in BC‐3s, with both circHIPK3 and DLL4 upregulated upon lytic replication and miR‐30c downregulated (Appendix Fig S5G–I). This highlights the potential importance of this circuit across KSHV infection.

**Figure 7:**
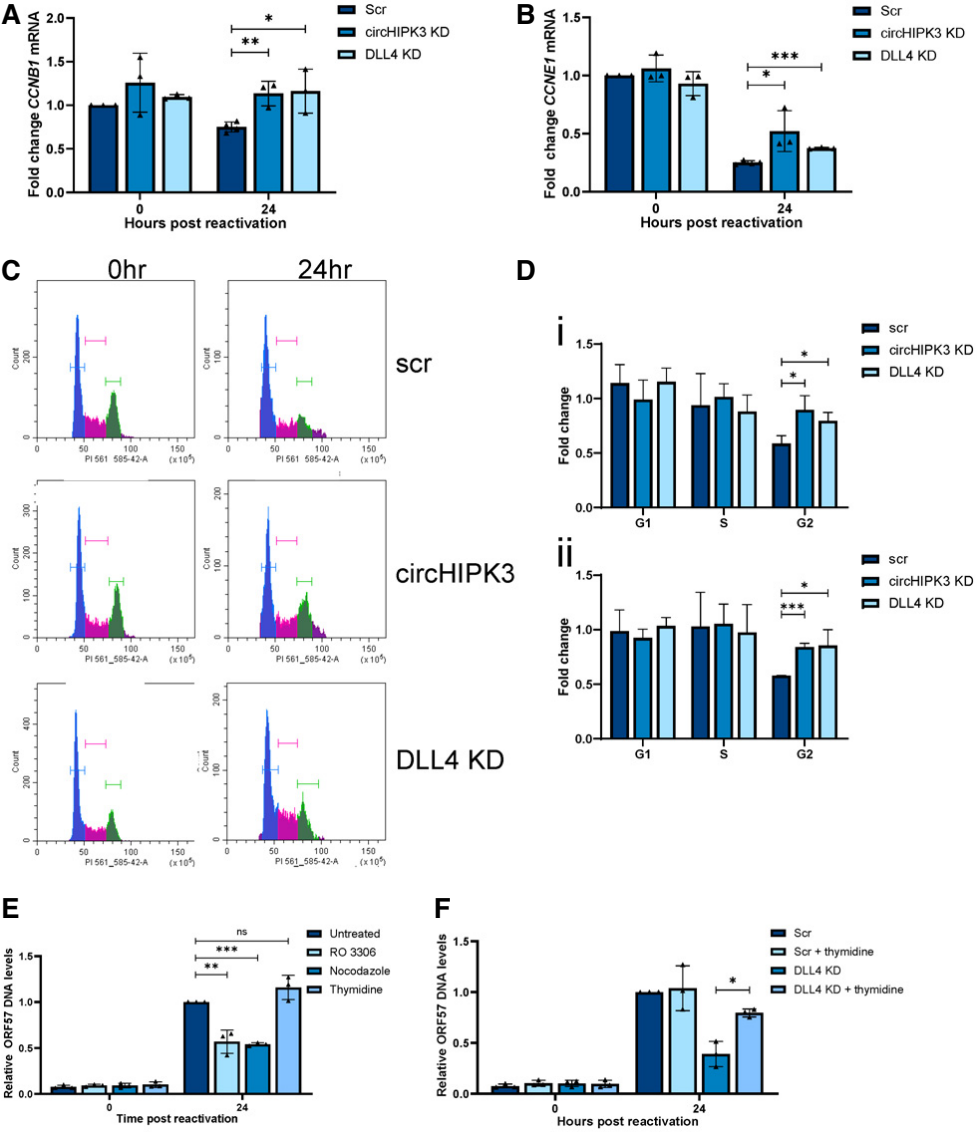
CircHIPK3:miR‐30c:DLL4 dysregulation affects cell cycle A, BqPCR of *CCNB1* and *CCNE1* levels in scr, circHIPK3 KD and DLL4 KD cells at 0 and 24 h post‐induction (*n* = 3).C, DRepresentative plot of cell cycle distribution at 48 h post‐induction in scr, circHIPK3 and DLL4 KD cells, with G1 phase (blue), S phase (pink) and G2/M phase (green) highlighted. (D) Analysis of fold change in number of cells in G1, S and G2/M phases of the cell cycle from 0 to 24 h post‐induction (*n* = 4) (i) or 0 to 48 h post‐induction (*n* = 3) (ii). Cells used were scr, circHIPK3 KD or DLL4 KD stable TREx cells and cycle analysis was performed using propidium iodide and flow cytometry (*n* = 3).EqPCR for ORF57 DNA levels in TREx cells at 48 h post‐lytic induction with treatment of RO 3306, nocodazole or thymidine (*n* = 3).FqPCR for ORF57 DNA levels at 48 h post‐lytic induction with treatment of RO 3306, nocodazole or thymidine in scr or DLL4 KD cell lines (*n* = 3). Data information: In (A, B and D–F) data are presented as mean ± SD. *P < 0.05, **P < 0.01 and ***P < 0.001 (unpaired Student’s *t*‐test). All repeats are biological. Source data are available online for this figure.

DLL4 is a transmembrane protein that acts as a ligand for Notch receptors 1 and 4. As such, it has multiple functions, for instance, modulating endothelial cell behaviour during angiogenesis (Bridge *et al*, 2012) and regulating cell cycle‐associated proteins (Emuss *et al*, 2009). Focusing on cell cycle‐associated proteins, we analysed several cyclins during KSHV lytic replication in TREx‐BCBL1‐RTA cells, comparing scrambled control, circHIPK3 KD and DLL4 KD cell lines. Lytic replication led to dysregulation of both *CCNE1* and *CCNB1*, whereas this downregulation was partly abrogated in both circHIPK3 and DLL4 KD cell lines (Fig 7A and B). Double KD of DLL4 and circHIPK3 was performed, utilising stable DLL4 KDs transfected with gapmeRs targeting circHIPK3, efficiency of the gapmeRs was analysed via qPCR (Appendix Fig S6A and B). Levels of *CCNE1* and *CCNB1* were assessed in the double KD cell line, with no cumulative effect seen, confirming that the impact of circHIPK3 on the cell cycle is likely through DLL4 (Appendix Fig S6C and D). Additionally, transfection of the miR‐30c mimic into DLL4 KD cells leads to no significant further reduction in *CCNE1* or *CCNB1,* with no cumulative effect on viral ORF65 levels either (Appendix Fig S6E–G).

As KSHV DNA replication is tied to viral manipulation of the cell cycle and our previously observed reductions in viral loads in both circHIPK3 and DLL4 KD cell lines, we assessed what effect dysregulation of the circHIPK3/miR‐30c/DLL4 regulatory circuit had upon KSHV‐mediated cell cycle dysregulation during lytic replication. Flow cytometry analysis using propidium iodide demonstrated that scrambled control TREx‐BCBL1‐RTA cells showed a significant drop in the number of cells in G2/M phase during lytic replication and a maintained number in G1 phase. In contrast, depletion of either circHIPK3 or DLL4 resulted in an increased number of cells in G2/M phase (Fig 7C and D). Together these data suggest that dysregulation of the circHIPK3/miR‐30c/DLL4 regulatory circuit affects cell cycle regulation to enhance KSHV lytic replication. This is clearly supported by results showing disruption of this axis at several points can inhibit virus replication.

To further confirm KSHV‐mediated dysregulation of the cell cycle is important for viral DNA replication, cells were treated with cell cycle inhibitors RO‐3306, nocodazole and thymidine and effects on the cell cycle were confirmed with flow cytometry (Appendix Fig S7A and B). Treatment with the G2/M phase arresting RO‐3306 and nocodazole led to a significant decrease in viral load in KSHV lytic cells (Fig 7E). In contrast, treatment with thymidine, a G1/S exit blocker, led to no decrease in viral load in scrambled control cells. However, more importantly, thymidine treatment of DLL4 KD cells, reversing DLL4‐mediated cell cycle changes, led to rescue of viral DNA replication (Fig 7F). This suggests that the effect of DLL4 on KSHV lytic replication in B cells is directly mediated through its cell cycle activities. Cell cycle dysregulation has been shown to be important for KSHV DNA replication, and we confirmed this with both the knockdown cell lines and cell cycle blocking inhibitors. Therefore, we hypothesised that due to this inhibition of viral DNA replication, formation of viral replication centres, where viral DNA replication occurs, would be reduced or delayed. Together this shows viral DNA replication is impaired in both circHIPK3 and DLL4 KD cell lines and through treatment with cell cycle inhibitors. Together these data suggest KSHV‐mediated dysregulation of the circHIPK3/miR30c/DLL4 axis is a novel mechanism for cell cycle regulation in B cells.

## Discussion

**Figure 8:**
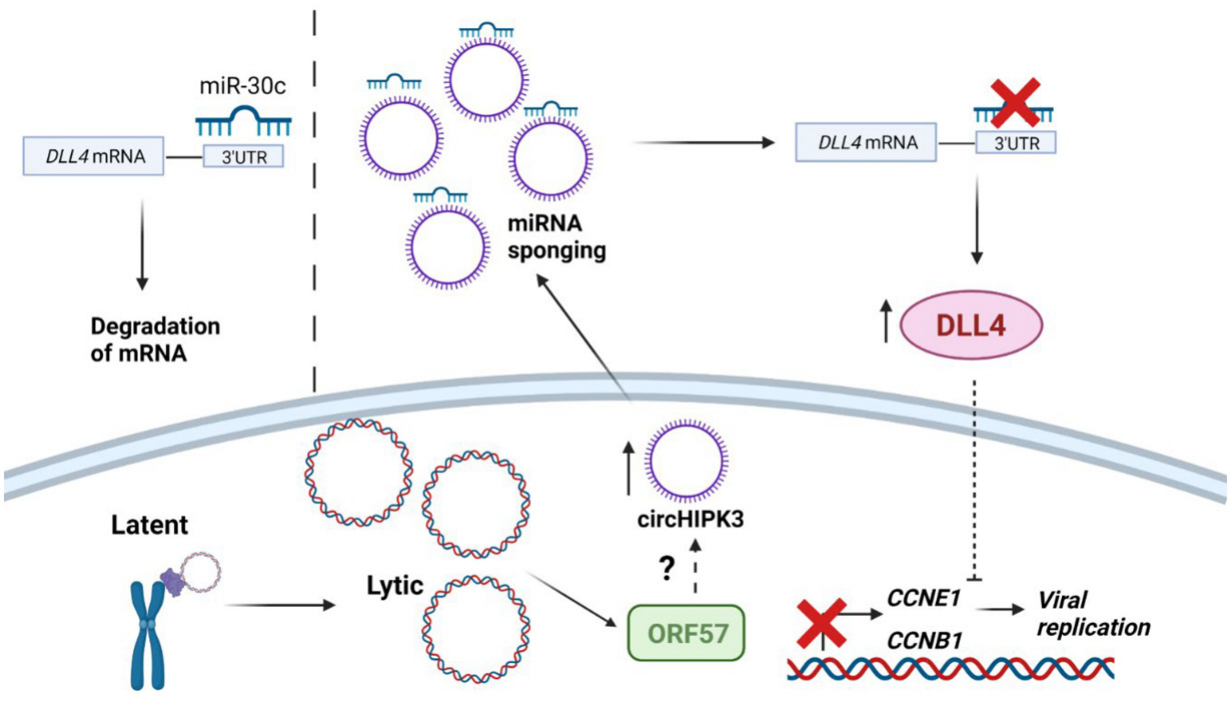
KSHV dysregulates a novel ncRNA network during lytic replication Schematic of proposed ncRNA network manipulated by KSHV. CircHIPK3 is upregulated by KSHV, partly mediated through ORF57, this leads to increased sponging of miR‐30c and increased DLL4 levels. DLL4 has downstream effects on cyclins leading to changes in the cell cycle aiding KSHV replication. Created with BioRender.com.

Despite circRNAs being relatively a recent discovery, functional analysis has suggested a wide range of crucial roles in cell regulation including transcriptional regulation, miRNA sponging and protein regulation (Li *et al*, 2018b; Wilusz, 2018). It is not surprising therefore the dysregulation of circRNAs is implicated in a range of diseases including diabetes, dementia and cancer (Akhter, 2018; Arnaiz *et al*, 2018; Zhang *et al*, 2018). Here, we show that the dysregulation of circRNAs also play a role in virus infection. We demonstrate that circHIPK3 is consistently upregulated during KSHV lytic replication and crucially, reversion of this upregulation leads to inhibition of viral replication and infectious virion production, highlighting the importance of KSHV replication. Furthermore, we performed functional analysis of the role circHIPK3 has during viral replication and have identified it as the first step in a novel non‐coding RNA regulatory network that is dysregulated during KSHV infection, summarised in Fig 8.

Previous research has identified circHIPK3 is capable of miRNA sponging, including miR‐7, miR‐124 and another member of the miR‐30 family: miR‐30a (Zheng *et al*, 2016; Zeng *et al*, 2018; Chen *et al*, 2019). Many of these miRNAs are classified as tumour suppressive, implicating circHIPK3 as a pro‐proliferative factor. Moreover, circHIPK3 is found upregulated in several cancers, including bladder, lung and HCC, and is therefore hypothesised to have regulatory roles in cell proliferation, cell cycle regulation and invasion (Wen *et al*, 2020; Xie *et al*, 2020; Zhang *et al*, 2020). However, the role of circHIPK3 has not previously been elucidated in viral infection. KSHV has been shown to be prolific at utilising ncRNAs to aid its replication and propagation, with its complex genome encoding both miRNAs and circRNAs (Qin *et al*, 2012; Toptan *et al*, 2018), while dysregulation of cellular miRNAs enables low‐immunogenicity post‐transcriptional regulation of many different targets. Through dysregulation of circRNAs, such as circHIPK3, it enables upstream regulation of these miRNAs and, through this ncRNA axis, allows subversion of cellular processes aiding viral replication and tumourigenesis. Similarly, a circARFGEF1/miR‐125a‐3p/GLRX3 axis has been shown to be required for KSHV vIRF1 induction of cell motility, proliferation and *in vivo* angiogenesis (Yao *et al*, 2021).

CircRNA functionality is tied to their localisation, with nuclear retained circRNAs interacting with RBPs or acting as transcriptional regulators (Li *et al*, 2015; Holdt *et al*, 2016), whereas using RNA FISH, circHIPK3 was shown to be localised in the cytoplasm, suggestive of a role in miRNA sponging. This was further supported by RIPs of biotinylated miR‐29b and miR‐30c. These RIPs showed enrichment of circHIPK3, confirming that circHIPK3 acts as a competitive endogenous RNA and binds to these miRNAs. CircHIPK3 was also enriched in Ago2 RIPs, implying it is interacting with the miRNA machinery. This once again supports its sponging function, with other well‐characterised miRNA‐sponging circRNAs such as circSRY and circCDR1 also associating with Ago2 (Du *et al*, 2017). Of particular note is that the enrichment was enhanced after transfection of miR‐29b and miR‐30c, suggesting when there are increased levels of target miRNAs interacting with Ago2, there is increased interaction by the sponging circRNA. Possibly either as the sponging circRNA is also upregulated as a compensatory mechanism or increased levels of the target miRNA can increase the stability of the interaction with Ago2, acting as a bridge.

Overexpression of miR‐29b and miR‐30c mimics had an inhibitory effect on KSHV lytic replication, therefore we hypothesise KSHV upregulates circHIPK3 to reduce the levels of these miRNAs. Crucially, circHIPK3 depletion inhibited viral replication, viral load and infectious virion production and also led to increases in miR‐29b and miR‐30c levels, implying a regulatory axis. Notably, analysis confirmed miR‐29b and miR‐30c downregulation did not occur at the primary or pre‐stages of miRNA biogenesis, once again implicating circHIPK3 as a miRNA sponge.

The exact mechanism of circHIPK3‐mediated dysregulation of the miRNAs is currently not elucidated, with circRNA sponging alone not sufficient to induce degradation and therefore decreased levels of the miRNAs. One potential mechanism for circRNA binding leading to active degradation is through target RNA–directed microRNA degradation (TDMD), where binding to highly complementary RNAs induces miRNA degradation (Fuchs Wightman *et al*, 2018). Interestingly, of the few miRNAs that have been shown to undergo TDMD, both miR‐29b and miR‐30c are among them, with TDMD able to occur even at low expression levels (Bitetti *et al*, 2018; Ghini *et al*, 2018). The exact requirements and factors in TDMD are still not fully known and appear to vary dependent on mechanism, however, a central mismatch bulge and high 3’ complementarity appear to be required (Sheu‐Gruttadauria *et al*, 2019), although binding site predictions for circHIPK3 and the miRNAs do not fully follow this basis.

Other potential models for degradation of miRNAs through RNA binding include binding leading to recruitment of degradation factors, relocalisation of the miRNA leading to its degradation or most similarly to TDMD: binding could lead to conformational changes that expose the miRNA to degradation machinery (Guo & Steitz, 2014). Of note, herpesviruses are particularly adept at regulating and inducing degradation of small RNAs through binding with lncRNAs, with HCMV, MCMV and the close relation of KSHV, herpesvirus saimiri (HVS), all having been shown capable of small RNA degradation (Guo & Steitz, 2014; McCaskill *et al*, 2015; Fuchs Wightman *et al*, 2018). However, at present, the mechanism and factors involved in this axis require further investigation.

miR‐30c was selected for further investigation over miR‐29b due to its more consistent effect on the virus and the increased circHIPK3 association with Ago2 in the presence of miR‐30c. CircHIPK3 depletion or overexpression of the miRNAs led to inhibition of KSHV lytic replication clearly demonstrating the importance of cellular circRNA dysregulation to enable virus‐mediated hijacking of cellular ncRNA networks enhancing viral replication.

The mechanism behind this dysregulation is not fully elucidated. However, our analysis suggests that the KSHV lytic protein, ORF57, may play a significant role. Initial investigations show circHIPK3 dysregulation occurs between 16 and 24 h upon lytic replication, coinciding with ORF57 expression during the viral cascade. Furthermore, ORF57 expression alone increased circHIPK3 levels. Interestingly, RIPs performed at 16 and 24 h after transfection indicated ORF57 bound to *HIPK3* and circHIPK3 with the ratios changing over time, suggesting a direct role in regulating the splicing. At present, the actual mechanism of ORF57‐mediated upregulation requires further investigation with cellular factors still to be identified.

ORF57 is a promiscuous RBP, with many interacting partners, as transfection of ORF57 into HEK‐293T cells leads to an increase in circHIPK3 levels without any other viral proteins, it is likely ORF57 recruits and utilises cellular proteins to aid this dysregulation; however, as of yet, these factors are unknown. Furthermore, we show its RNA‐binding properties are likely important in this role, with the ORF57 RNA‐binding negative mutant RGG1/2 unable to bind to circHIPK3 (Appendix Fig S3C). Notably, transfections of RGG1/2 into HEK‐293Ts only led to a partial loss in circHIPK3 upregulation, suggesting other factors may have a role. Interestingly, ORF57 is known to form a dimer (Yuan *et al*, 2018); many of the other known circRNA biogenesis‐promoting proteins act through dimerisation, for instance, QKI and Mbl (Ashwal‐Fluss *et al*, 2014; Conn *et al*, 2015). Here, the protein monomers bind to the upstream and downstream splice sites, and as they dimerise they bring the splice sites in close contact promoting circularisation; it is possible that ORF57 promotes circHIPK3 biogenesis by this mechanism. Unfortunately, the dimerisation negative ORF57 mutant we utilised was too unstable for further investigation (Appendix Fig S3D) (Ebbesen *et al*, 2017). Alternatively ORF57 role in circHIPK3 dysregulation may be through promotion of stability rather than biogenesis, with previous research identifying ORF57 as a key stability factor in the viral ncRNA transcript PAN (Ruiz *et al*, 2019). Further research is now needed to elucidate exact role of ORF57 in circRNA biogenesis.

DLL4 was identified as a potential target of the circHIPK3/miR‐30c ncRNA regulatory axis. Supporting this potential network, we have shown that DLL4 is upregulated following KSHV lytic reactivation in TREx‐BCBL1‐RTA cells. Importantly, upon circHIPK3 depletion and miR‐30c overexpression, we show that DLL4 upregulation is significantly reduced, whereas further depletion of miR‐30c leads to additional increases in DLL4 levels. Moreover, DLL4 depletion via stable lentivirus KDs has an inhibitory effect on viral replication, affecting late ORF65 protein production, viral load and infection virion production. The importance of DLL4 to KSHV replication has been previously investigated, with the viral protein vGPCR upregulating DLL4 in an ERK‐dependent manner leading to activation of Notch4 signalling in endothelial cells, although most of the studies focus on latent upregulation of DLL4 in endothelial cells (Emuss *et al*, 2009; Liu *et al*, 2010). Due to the importance of DLL4 in viral replication, this redundancy is not surprising. Additionally, non‐coding RNA expression patterns are cell type specific, therefore whether circHIPK3 and miR‐30c are functionally active in KSHV‐infected endothelial cells needs further investigation. Furthermore, exogenous miR‐30c was shown to inhibit KSHV replication through targeting of DLL4, although the functional role of endogenous miR‐30c has not been previously investigated in KSHV (Bridge *et al*, 2012).

We demonstrate through flow cytometry analysis that the circHIPK3:miR‐30c:DLL4 circuit has a role in regulating the host cell cycle during KSHV replication. Although the role of KSHV in the cell cycle during latency is understood, with v‐cyclin playing a key role, the role of the cell cycle during KSHV lytic replication is debated, with lytic replication either requiring entry into S phase or G1 arrest (Wu *et al*, 2003; Jones *et al*, 2014; Hollingworth *et al*, 2020). Our results show that levels of cells in G1 phase slightly increase from 0 to 24 h while S phase is not significantly altered. This increase in cells into G1 was also noticeable even when treated with G2/M inhibitors. This increase in G1 phase was also accompanied by a loss of cells in G2/M phase with the corresponding peak far less distinct. Other viruses have also been observed promoting G1 phase, notably EBV, a human gamma‐herpesvirus, which has been observed promoting G1 phase during lytic replication (Rodriguez *et al*, 2001; Kudoh *et al*, 2003). Furthermore, other herpesviruses including HCMV and HSV‐1 also require G1 entry for successful lytic replication (Salvant *et al*, 1998; Jordan *et al*, 1999). Interestingly, these studies found G1 exit into S phase was blocked upon infection; however, several cellular factors found in S phase were still promoted, it is hypothesised that these cellular conditions allow successful viral DNA replication using cellular proteins, however, prevent competition from cellular DNA synthesis which occurs in S phase. Further examination is needed to identify whether this occurs during KSHV lytic replication.

Both circHIPK3‐ and DLL4‐depleted cell lines demonstrated the same phenotype using flow cytometry analysis, with a prevention of the G2/M dysregulation seen during lytic replication in scramble control cells, indicative that KSHV‐mediated cell cycle dysregulation is being partly blocked. DLL4 has previously been characterised to regulate cyclin and cell cycle factors (Emuss *et al*, 2009), therefore we hypothesise that dysregulation of this ncRNA network at any phase prevents KSHV‐mediated dysregulation of the cell cycle leading to inhibition of productive viral replication. The regulation of cell cycle through circHIPK3 function aligns with its observed upregulation in many cancers leading to its identification as a pro‐oncogenic circRNA, as dysregulation of the cell cycle is a key step in the development of cancer (Wen *et al*, 2020). Interestingly, previous research has shown KSHV dysregulates this cell cycle in the development of cancer, however, it has focused on latent dysregulation, and we have therefore shown KSHV dysregulates circHIPK3 during lytic replication to dysregulate the cell cycle (Moore, 2007).

Supporting this hypothesis, we found the dysregulation of cellular cyclins during lytic replication was reduced in circHIPK3 and DLL4 KD cell lines. Moreover, to confirm cell cycle dysregulation is important for KSHV replication, lytic cells were treated with various known cell cycle inhibitors. Arrest in the G2/M phase through RO‐3306 and nocodazole treatment, reversing the observed loss of cells in G2/M phase upon typical lytic replication, led to a significant decrease in viral load. In contrast, addition of thymidine, a G1/S exit blocker, had no impact on viral replication, likely due to lytic replication causing a slight increase in cells in G1 phase, implying KSHV lytic replication requires cells to be in G1 phase. Importantly, addition of thymidine to DLL4 KD cells reversed the anti‐viral affect previously observed due to increases in cells in G1 phase and a decrease in G2/M phase, once again suggesting that DLL4 and circHIPK3 aid viral replication at least partly through cell cycle dysregulation (Emuss *et al*, 2009).

In summary, elucidating the role of circRNAs within viral infection is still in its infancy, however, in recent years, increasing number of studies have identified viral encoded circRNAs and also RNA‐Seq‐based experiments have highlighted aberrant circRNA expression profiles during infection. We have identified the host cell circRNA, circHIPK3, is dysregulated and essential for KSHV lytic replication. Notably, it forms the cornerstone of a novel ncRNA network, regulating the downstream targets of miR‐30c and DLL4, which are in turn, all essential for KSHV lytic replication. The finding of this novel network suggests there may be many more roles for circRNAs both in KSHV and in other viruses’ infection.

## Materials and Methods

### Cell culture

TREx‐BCBL1‐RTA cells, a B‐cell lymphoma cell line latently infected with KSHV engineered to contain a doxycycline‐inducible myc‐Rta, were a gift from Professor JU Jung (University of Southern California). TREx‐BCBL1‐RTA cells were cultured in RPMI1640 with glutamine (Gibco), supplemented with 10% foetal bovine serum (FBS) (Gibco), 1% P/S (Gibco) and 100 µg/ml hygromycin B (ThermoFisher). HEK‐293T cells were purchased from ATCC and cultured in Dulbecco’s modified Eagle’s medium with glutamine (DMEM) (Lonza), supplemented with 10% FBS and 1% P/S. BC‐3 cells were purchased from the ATCC and cultured in RPMI1640 with glutamine (Gibco) supplemented with 20% FBS and 1% P/S. HEK‐293T‐rKSHV.219 were kindly provided by Dr Jeffery Vieira (University of Washington) and were cultured in Dulbecco’s modified Eagle’s medium with glutamine (DMEM) (Lonza), supplemented with 10% FBS, 1% P/S and 3 μg/ml puromycin (Gibco). All cell lines tested negative for mycoplasma. Virus lytic replication in TREx‐BCBL1‐RTA cells was induced via addition of 2 µg/ml doxycycline hyclate (Sigma‐Aldrich). HEK‐293T‐rKSHV.219 cells and BC‐3s were induced via addition of 20 ng/ml TPA and 4 mM sodium butyrate. All cells were cultured at 37°C at 5% CO_2_. Fifty nM miRNA mimics (abm) and 20 nM antagomiRs (ThermoFisher) were transfected using Lipofectamine RNAi Max (ThermoFisher) and left for 24 h before addition of doxycycline and further experiments. Plasmids were transfected in a 1:2 ratio with Lipofectamine 2000 (ThermoFisher). CircHIPK3 LNA GapmeRs (Qiagen) were incubated at 100 nM for 24 h prior to lytic induction in DLL4 KD TREx‐BCBL1‐RTA cells for double‐KD experiments.

### RNA extraction, cDNA synthesis and qPCR

Total RNA was extracted using Monarch Total RNA Miniprep kit (NEB) as per manufacturer’s protocol. One microgram RNA was reverse transcribed using LunaScript RT SuperMix Kit (NEB). qPCR was performed using synthesised cDNA, GoTaq qPCR MasterMix (promega) and the appropriate primer. qPCR was performed on Rotorgene Q and analysed by the ΔΔCT method against a housekeeping gene as previously described before plotting as fold change (Livak & Schmittgen, 2001; Baquero‐Perez *et al*, 2019).

For miRNA analysis, total RNA was extracted using TRIzol (Invitrogen) as per manufacturer’s protocol. RNA was then DNase treated using DNase I (18068015 Thermo Fisher) as per protocol. One microgram RNA was reverse transcribed using miScript II RT kit (Qiagen) as per protocol before qPCR using miScript SYBR Green kit (Qiagen) and analysis by the ΔΔCT against SNORD68.

### Plasmids and antibodies

Antibodies used in western blotting are listed below: ORF65 (CRB crb2005224, 1/100), ORF57 (Santa Cruz sc‐135747 1/1,000), GAPDH (Proteintech 60004‐1‐Ig 1/5,000), GFP (Proteintech 66002‐1‐ig 1/5,000) and DLL4 (Proteintech 21584‐1‐AP 1/200). Ago2 (abcam ab186733) was used in RIPs.

pVSV.G and psPAX2 were a gift from Dr Edwin Chen (University of Westminster, London). PLKO.1 TRC cloning vector was purchased from Addgene (gift from David Root; Addgene plasmid #10878) with shRNAs against *DLL4* and circHIPK3 cloned into it. psiCheck2 was a gift from Dr James Boyne (Leeds Beckett University). GFP, GFP‐ORF50, GFP‐ORF57 and GFP‐ORF57 RGG1/2 have been described previously (Gould *et al*, 2009; Schumann *et al*, 2016). Primers are listed in Appendix Table S1.

### Immunoblotting

Cell lysates were separated using 8–12% polyacrylamide gels and transferred to Amersham Nitrocellulose Membranes (GE healthcare) via Trans‐blot Turbo Transfer system (Bio‐Rad). Membranes were blocked in TBS + 0.1% tween with 5% wt/vol dried skimmed milk powder. Membranes were probed with appropriate primary antibodies and secondary horseradish peroxidase conjugated IgG antibodies at 1/5,000 (Dako Agilent). Proteins were detected with ECL Western Blotting Substrate (Promega) or SuperSignal™ West Femto Maximum Sensitivity Substrate (ThermoFisher) before visualisation with G box (Syngene).

### Fluorescence *in situ* hybridisation

TREx‐BCBL‐1‐RTA cells were seeded onto poly‐L‐lysine (Sigma‐Aldrich)‐coated coverslips and 2 µg/ml doxycycline hyclate (Sigma‐Aldrich) was added 3 h later. FISH was performed 24 h later using ViewRNA Cell Plus Assay (ThermoFisher) as manufacturer’s protocol. Coverslips were mounted using Vectashield Hardset Mounting Medium with DAPI (Vector laboratories). Images were obtained using a Zeiss LSM880 Inverted Confocal Microscope and processed using ZEN 2009 imaging software (Carl Zeiss) as previously described (Baquero‐Perez & Whitehouse, 2015).

### Immunofluorescence

TREx‐BCBL‐1‐RTA cells were seeded onto poly‐L‐lysine‐coated coverslips and 2 µg/ml doxycycline hyclate (Sigma‐Aldrich) was added 3 h later. Cells were fixed with 15 min with 4% paraformaldehyde and permeabilised with PBS + 1% Triton. All further incubation steps occurred at 37°C. Coverslips were blocked for 1 h with PBS and 1% BSA before 1 h incubation with the appropriate primary antibody and followed by 1 h with Alexa‐Fluor conjugated secondary antibody 488 (Invitrogen 1/500). Coverslips were mounted using Vectashield Hardset Mounting Medium with DAPI (Vector laboratories). Images were obtained using a Zeiss LSM880 Inverted Confocal Microscope and processed using ZEN 2009 imaging software (Carl Zeiss) as previously described (Baquero‐Perez & Whitehouse, 2015).

### Viral reinfection and viral load assays

TREx‐BCBL‐1s were induced for 72 h before being spun down and supernatant was added to 293Ts in a 1:1 ratio with DMEM. Twenty‐four hours after addition of supernatant, cells were harvested and RNA extracted, reverse transcribed before qPCR analysis. For viral load, TREx‐BCBL‐1s were induced for 72 h before harvesting. DNA was extracted using DNeasy Blood and Tissue Kit (Qiagen) as per manufacturer’s instructions before qPCR analysis.

### RNA immunoprecipitations

For biotinylated RIPs, HEK‐293T cells were seeded and transfected with 20 nM biotinylated LNA miR‐29b or miR‐30c (Qiagen) in combination with 2 μl Lipofectamine RNAi Max (ThermoFisher). Twenty‐four hours post‐transfection, RIPs were performed as per manufacturer’s directions using Dynabeads MyOne Streptavidin T1 (ThermoFisher). RNA was extracted and purified using TRIzol LS (Invitrogen) as per manufacturer’s instructions before analysis via qPCR. Samples were analysed using fold enrichment over per cent inputs.

Ago2 RIPs were performed on TREx‐BCBL‐1 RTA cells using EZ‐Magna RIP RNA‐binding Immunoprecipitation kit (Merck millipore) as per manufacturer’s instructions. RNA was extracted and purified using TRIzol LS (Invitrogen) as per manufacturer’s instructions before analysis via qPCR. Samples were analysed using fold enrichment over per cent inputs.

For GFP RIPs, HEK‐293Ts were transfected with 2 μg GFP or GFP‐ORF57 plasmids with DNA in a 1:2 ratio with Lipofectamine 2000 (Thermo Fisher Scientific). Cells were lysed before incubated with GFP‐Trap Agarose beads overnight at 4°C (Chromotek) and washed as manufacturer’s protocol. Samples were incubated with Proteinase K buffer (containing 10 mM Tris pH 7.5, 150 mM NaCl, 0.5 mM EDTA, 10% SDS and proteinase K) for 30 min at 55°C before RNA extracted via TRIzol LS (Invitrogen) as per manufacturer’s instructions before analysis via qPCR. Samples were analysed using fold enrichment over per cent inputs.

### 3’ UTR luciferase assay

The 3’UTR of *DLL4* was identified using NCBI AceView before cloned into psiCheck2 plasmid. HEK‐293T cells were transfected with 50 nM miR‐30c or a scrambled control alongside psi‐check2 plasmid. Luciferase reporter assays were performed 24 h post‐transfection using Dual Luciferase Reporter Assay System (Promega) as per the manufacturer’s directions.

### Lentivirus‐based shRNA knockdown

Lentiviruses were generated by transfection of HEK‐293T cells seeded in 12‐well plates using a three‐plasmid system. Per 12‐well, 4 µl of Lipofectamine 2000 (Thermo Scientific) was used together with 1.2 µg of pLKO.1 plasmid expressing shRNA against the protein of interest, 0.65 µg of pVSV.G and 0.65 µg psPAX2. pVSV.G and psPAX2 were a gift from Dr. Edwin Chen (University of Westminster, London). Eight hours post‐transfection, media were changed with 1.5 ml of DMEM supplemented with 10% (v/v) FCS. Two days post‐transfection, viral supernatants were harvested, filtered through a 0.45 µm filter (Merck Millipore) and immediately used for transductions of TREx BCBL1‐RTA cells. Cells (500,000) in 12‐well plates were infected by spin inoculation for 60 min at 800 *g* at room temperature, in the presence of 8 μg/ml of polybrene (Merck Millipore). Three µg/ml Puromycin (Gibco) was added 48 h after transduction before KD analysed via qPCR and western blot if appropriate.

### Site‐directed mutagenesis

Site‐directed mutagenesis of GFP‐ORF57 for W292A mutant was carried out using QuikChange site‐directed mutagenesis kit (Agilent technologies) as per manufacturer’s instructions.

### Cell cycle analysis

Cells were seeded, harvested as required and followed by fixing overnight in 70% ethanol at −20°C. Cells were washed in PBS containing 0.5% BSA, before incubation for 30 min at room temperature in PBS containing 0.5% BSA, 5 µg/ml RNase A (ThermoFisher) and 25 µg/ml propidium iodide (Sigma). Samples were analysed by CytoFlex S Benchtop Flow Cytometer (Beckman Coulter). For cell cycle drug treatment, TREX‐BCBL‐1 cells were pre‐treated for 16 h with 10 µM RO‐3306, 0.5 µM nocodazole or 2 mm thymidine before addition of doxycycline. Viral load was measured after 48 h.

### miR‐seq analysis

Total RNA was extracted from TREx‐BCBL‐1s at 0, 16 and 24 h post‐lytic induction. Small RNA libraries were prepared using the TruSeq Small RNA Library Prep kit (Illumina). Samples were assessed using Agilent High Sensitivity D1000 Screen Tape Station and gel electrophoresis and size‐based extraction performed. cDNA libraries were analysed via Illumina HiSeq (Illumina) by University of Leeds NGS facility and data deposited at Gene Expression Omnibus (GSE186652).

### RNA‐seq analysis

Total RNA was extracted from TREx‐BCBL‐1s at 0 and 18 h post‐lytic induction. mRNA sequencing libraries were created using an Illumina TruSeq Stranded mRNA Sample Prep Kit (Illumina). cDNA libraries were generated and analysed using an Agilent Technologies 2100 Bioanalyze. Libraries were sequenced using a HiSeq (Illumina) 75 sequencing platform.

### Bioinformatics analysis of sequencing datasets

Raw reads were processed for RNA expression using standard bioinformatics pipeline. Quality filtered (Q < 20) and adapter trimmed reads (Trimmomatic v0.39) (Bolger *et al*, 2014) were aligned to the GRCh38/hg38 assembly of the human genome using Bowtie2 (V 2.4.2) or HISAT2 (V 2.1.0) (Langmead & Salzberg, 2012; Pertea *et al*, 2016) for miRNA and mRNA expressions, respectively. Then, the counts in different genomic features were generated using HTSeq (v0.11.1) (Anders *et al*, 2015) on human microRNA annotations (miRbase.org) or GRCh38 annotation (GENCODE Release 32). Expression levels were normalised by “counts per million” (CPM). Differential expression (DE) analyses between two KSHV replication times were performed using limma R package. The DE miRNAs and mRNA were defined at adjusted *P*‐value < 0.05. To reduce rate of false discovery rate in the DE analysis, we included only transcripts with at least 1 CPM in three samples.

### Microarray analysis

For microarray analysis of miR‐30c and miR‐29b expression, datasets GSE55625 and GSE18437 from NCBI Geo were used and analysed via GEO2R, based on the limma method.

### *In* *silico* analysis of global miR‐30c‐dependent changes

To identify the mRNA targets of miR‐30c, we extracted all its mRNA interactions from the miRTarBase database (Hsu *et al*, 2011). Next, we separated the mRNA expression values during KSHV replication at 20 h into, (i) mRNAs targeted by miR‐30c above and (ii) the rest and compared their distribution with the Kolmogorov–Smirnov test.

### Statistical analysis

Except otherwise stated, graphical data shown represent mean ± standard deviation of mean (SD) using three or more biologically independent experiments. Differences between means were analysed by unpaired Student’s *t*‐test, or distribution with two‐sample Kolmogorov–Smirnov test as detailed in the figure legends. Statistics was considered significant at *P* < 0.05, with *P < 0.05, **P < 0.01 and ***P < 0.001.

## Author contributions

**Katherine L Harper:** Conceptualization; Data curation; Formal analysis; Investigation; Writing—original draft; Writing—review and editing. **Timothy J Mottram:** Data curation; Investigation; Writing—review and editing. **Chinedu A Anene:** Data curation; Formal analysis; Writing—review and editing. **Becky Foster:** Investigation; Writing—review and editing. **Molly R Patterson:** Resources; Writing—review and editing. **Euan McDonnell:** Data curation; Writing—review and editing. **Andrew Macdonald:** Resources; Writing—review and editing. **David Westhead:** Funding acquisition; Writing—review and editing. **Adrian Whitehouse:** Conceptualization; Formal analysis; Supervision; Funding acquisition; Writing—original draft; Project administration; Writing—review and editing.

In addition to the CRediT author contributions listed above, the contributions in detail are:

Conceptualisation (KLH and AW); Data curation (KLH, TJM, CAA and EM); Formal Analysis (KLH and CAA); Funding acquisition (AW and DW); Investigation (KLH, BF and TJM); Reagents (MRP and AM); Writing – original draft (KLH and AW) and Writing – review & editing (all authors).

## Disclosure and competing interests statement

The authors declare that they have no conflict of interest.

## Supporting information



Appendix

Source Data for Appendix

Source Data for Figure 1

Source Data for Figure 2

Source Data for Figure 3

Source Data for Figure 4

Source Data for Figure 5

Source Data for Figure 6

Source Data for Figure 7

## Data Availability

RNA‐Seq data: Gene Expression Omnibus GSE186652 (https://www.ncbi.nlm.nih.gov/geo/query/acc.cgi?acc=GSE186652).
