# Supplementary material for: Dysregulation of the miR‐30c/DLL4 axis by circHIPK3 is essential for KSHV lytic replication
Source: EMBO Rep. 2022 Mar 3;23(5):e54117. doi: 10.15252/embr.202154117 (PMC9066072; doi:10.15252/embr.202154117)
Supplement: Supplementary file 1 — Appendix [file EMBR-23-e54117-s008.pdf]

Appendix Contents

Appendix Figure S1: pg 2

Appendix Figure S2: pg 4

Appendix Figure S3: pg 5

Appendix Figure S4: pg 6

Appendix Figure S5: pg 7

Appendix Figure S6: pg 8

Appendix Figure S7: pg 10

Appendix Table S1: pg 11

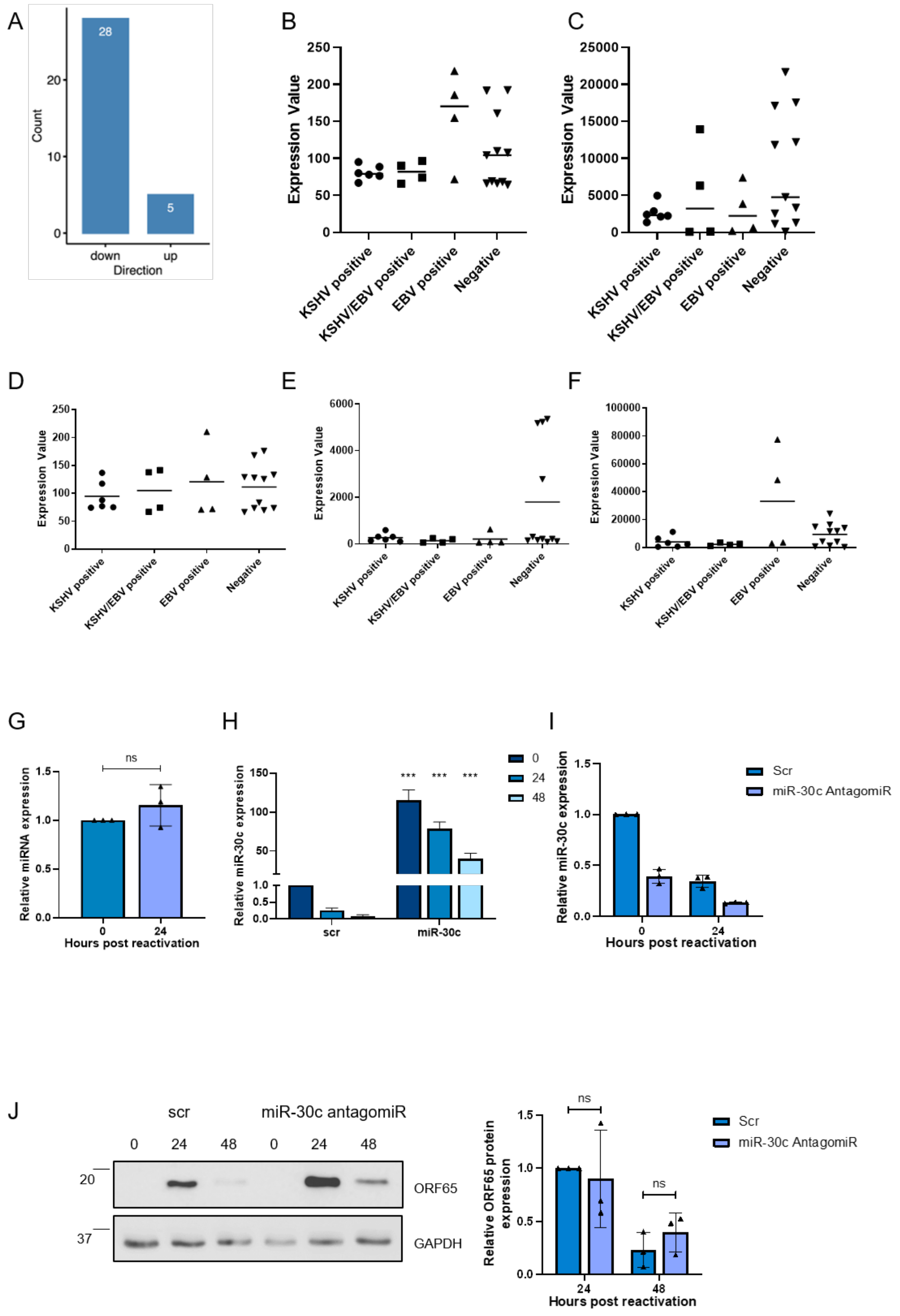

Appendix Figure S1:

(A) Bar plot of the direction of the 23 differentially expressed miRNAs identified using miR-Seq during KSHV replication

(B-F) Scatter plot data of miRNAs from GSE18437 with KSHV positive samples (n=6), KSHV + EBV positive samples (n=4), EBV positive samples (n=4) and KSHV + EBV negative samples (n=11). miRNAs are in order: miR-30c **(B)**, miR-29b **(C)**, miR-128 **(D)**, miR-27 **(E)** and miR-92 **(F).**

(G) qPCR analysis of levels of miR-29b-3p at 0 and 24 hours post-induction in TREx cells. SNORD68 was used as a housekeeper (n=3).

(H) qPCR analysis of miR-30c levels, TREx cells were transfected with a scrambled control or a miR-30c mimic before lytic replication induced 24 hours after transfection. miR-30c levels were analysed at 0, 24 and 48 hours post lytic induction. SNORD68 was used as a housekeeper (n=3).

(I) qPCR analysis of miR-30c levels after transfection with a scrambled control or a miR-30c antagomiR. 24 hours post transfection the TREx, cells were induced (n=3).

(J) Representative western blot of ORF65 levels at 0, 24 and 48 hours post induction in TREx cells. 24 hours prior to induction cells were transfected with either a scrambled control or a miR-30c antagomiR. GAPDH was used as a loading control. Densitometry analysis is n=3.

Data information: In G-J data are presented as mean ± SD. *P<0.05, **P<0.01, ***P<0.001 (Unpaired Student's t-test). All repeats are biological.

A

B

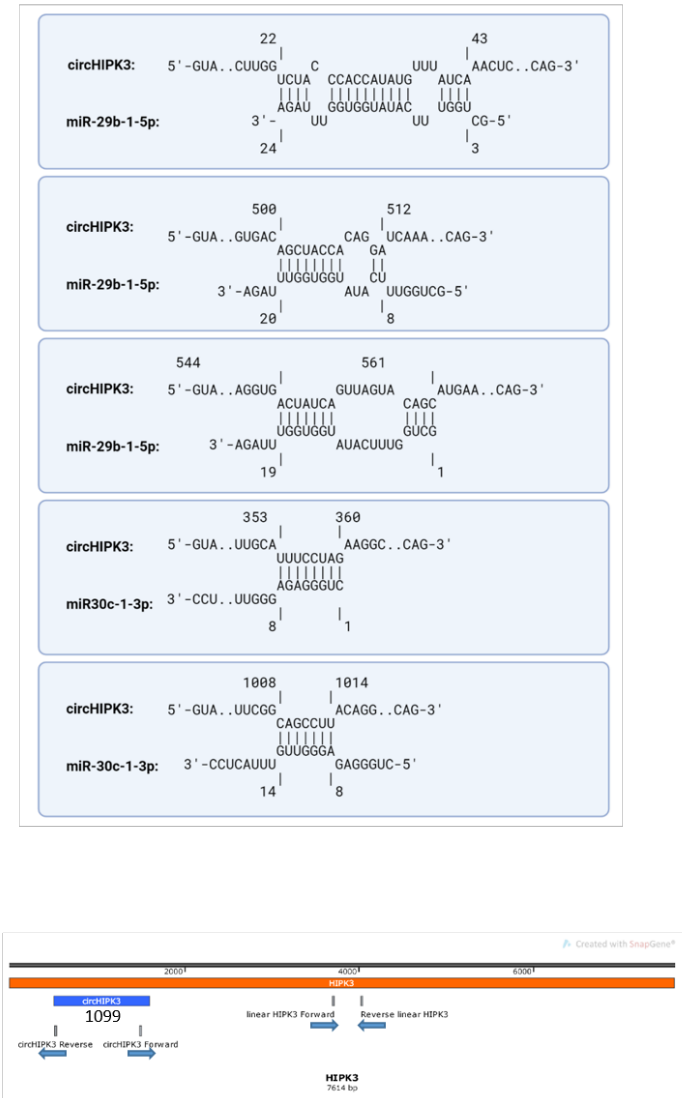

Appendix Figure S2:

(A) RNA binding predictions for circHIPK3 and miR-29b/miR-30c

(B) Schematic of the *HIPK3* gene with location of the circHIPK3 and primer sets used marked. Created using SnapGene software.

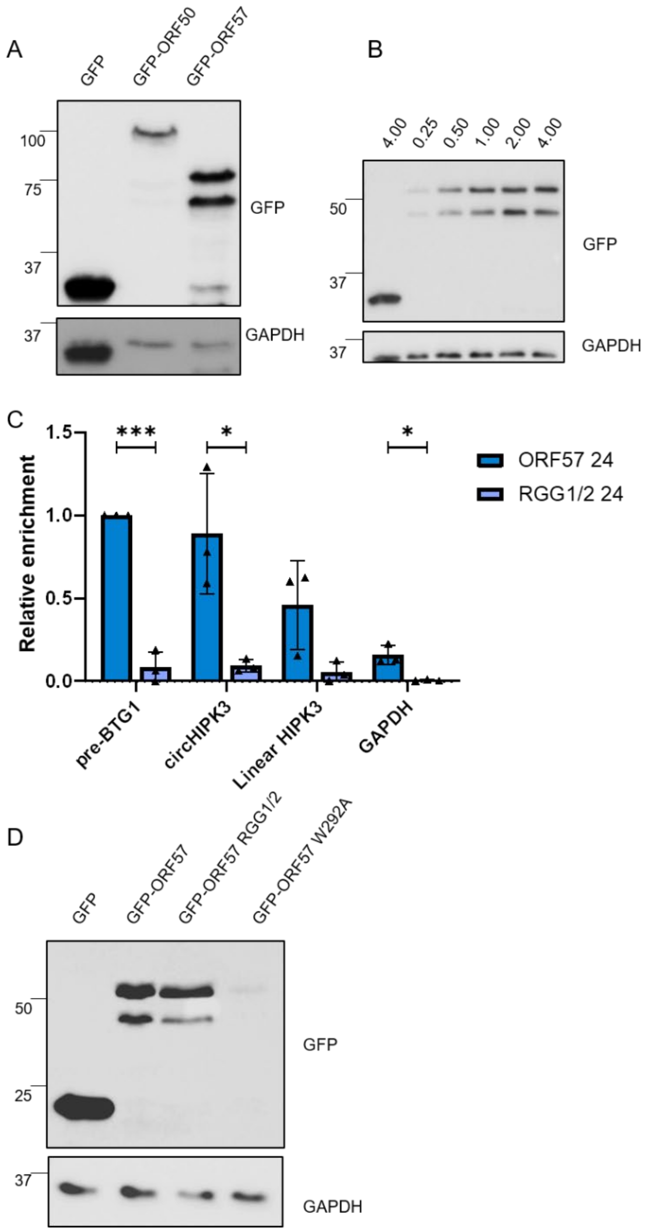

Appendix Figure S3:

(A) Western blot for GFP at 24 hours post-transfection in HEK-293Ts of GFP, GFP-ORF50 and GFP-ORF57. GAPDH was used as a loading control.

(B). Western blot for GFP at 24 hours post-transfection in HEK-293Ts of 4 µg GFP or varying amounts of GFP-ORF57.

(C) qPCR analysis GFP RIPs in GFP-ORF57 or GFP-ORF57 RGG1/2 transfected HEK-293Ts at 24 hours (n=3).

(D) Western blot for GFP at 24 hours post-transfection in HEK-293Ts of GFP, GFP-ORF57, GFP-ORF57 RGG1/2 and GFP-ORF57 W292A. GAPDH was used as a loading control.

Data information: In C data are presented as mean ± SD. *P<0.05, **P<0.01, ***P<0.001 (Unpaired Student's t-test). All repeats are biological.

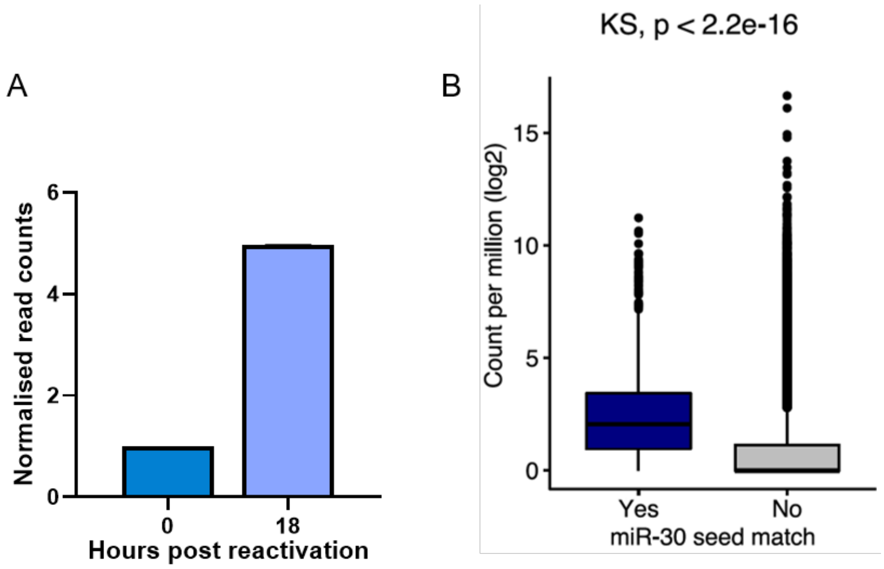

Appendix Figure S4:

(A) Levels of *DLL4* in mRNA Seq at 0 and 18 hours normalised to read count at 0hrs. N=2

(B) Box plot of the mRNA expression levels (CPM) between miR-30 targets and rest during KSHV replication at 20 hours. Two sample Kolmogorov-Smirnov test (KS, p) is indicated on top of the figure.

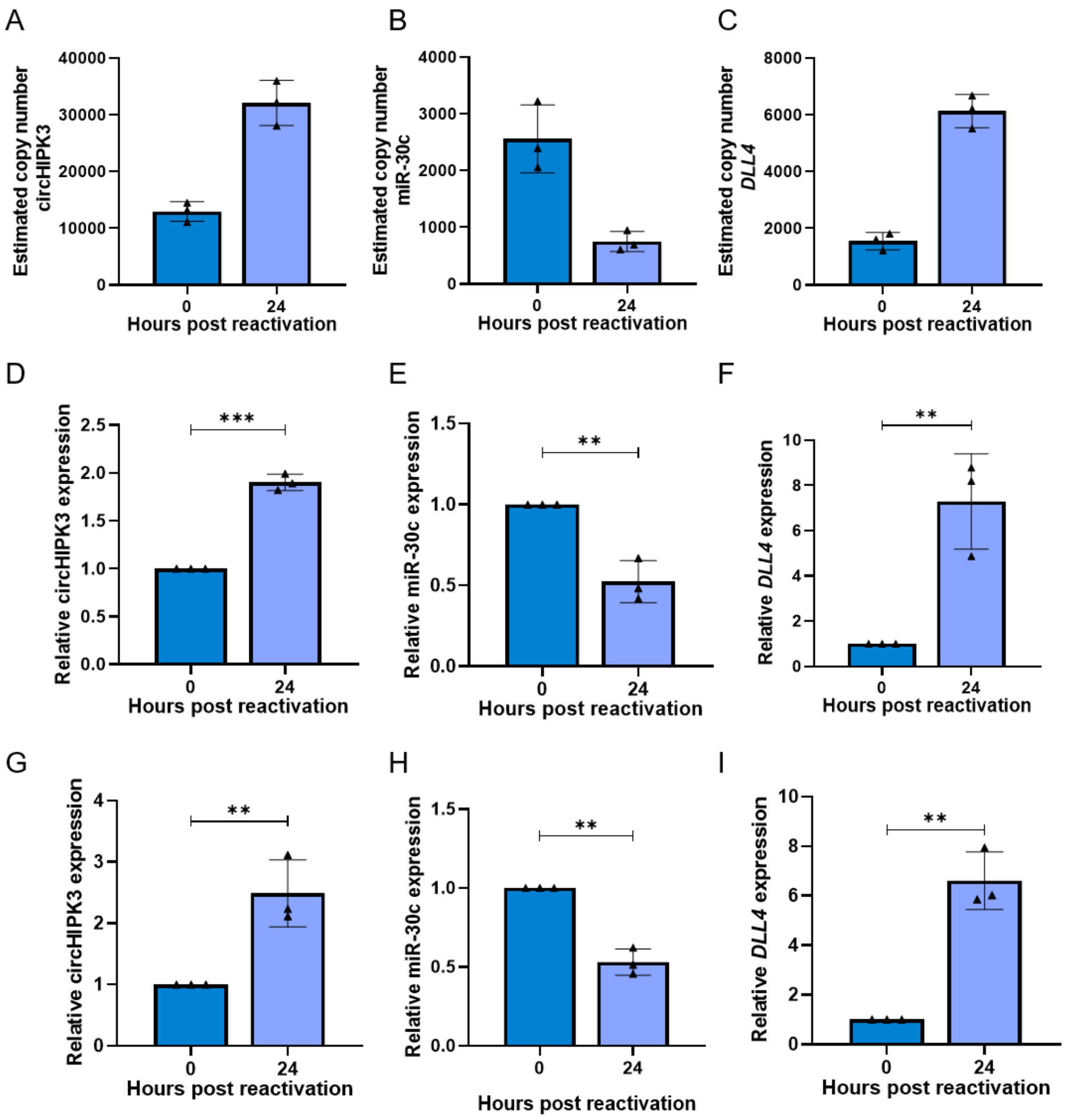

Appendix Figure S5:

(A-C) Copy number estimates per 5 ng TREx-BCBL-1-Rta cells RNA, calculated using the standard curve method for circHIPK3 **(A)** miR-30c **(B)** and *DLL4* **(C).** (n=3).

(D-F) qPCR analysis of circHIPK3 **(D)**, miR-30c **(E)** and *DLL4* **(F)** at 0 and 24 hours post lytic induction in HEK-293T-rKSHV.219 cells. GAPDH was used as a housekeeper for circHIPK3 and *DLL4* while SNORD68 was used as a housekeeper for miR-30c (n=3).

(G-I) qPCR analysis of circHIPK3 **(G)**, miR-30c **(H)** and *DLL4* **(I)** at 0 and 24 hours post lytic induction in BC-3 cells. GAPDH was used a housekeeper. (n=3)

Data information: In A-I data are presented as mean ± SD. *P<0.05, **P<0.01, ***P<0.001 (Unpaired Student's t-test). All repeats are biological.

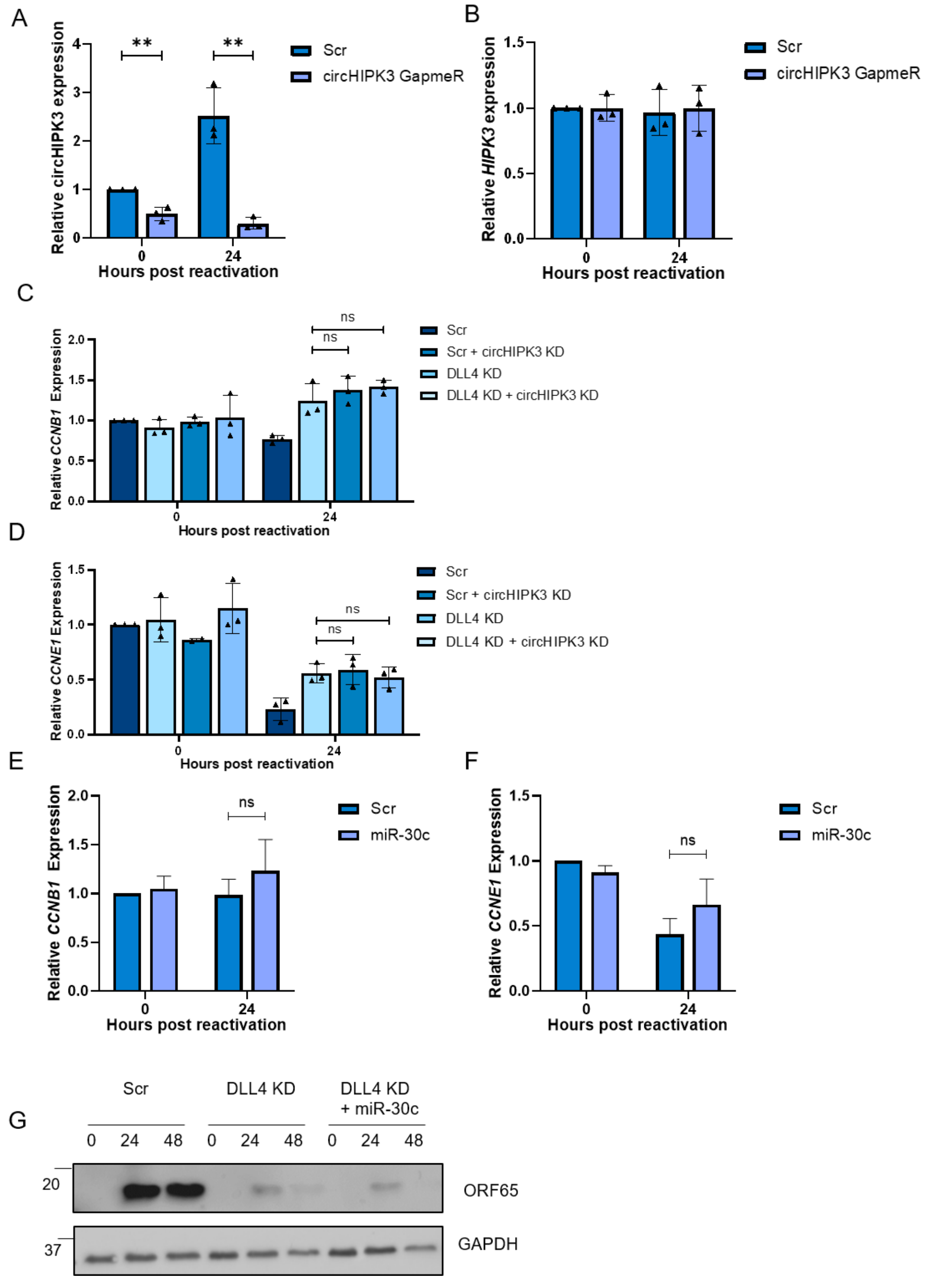

Appendix Figure S6:

(A) qPCR analysis of circHIPK3 levels in scr and circHIPK3 GapmeR treated TREx cells at 0 and 24 hours post lytic induction. GAPDH was used as a housekeeper (n=3).

(B) qPCR analysis of *HIPK3* levels in scr and circHIPK3 GapmeR treated TREx cells. GAPDH was used as a housekeeper (n=3).

(C) qPCR analysis of *CCNB1* expression in circHIPK3 and DLL4 KD TREx cells at 0 and 24 hours post lytic induction. GAPDH was used as a housekeeper (n=3).

(D) qPCR analysis of *CCNE1* expression in circHIPK3 and DLL4 KD TREx cells at 0 and 24 hours post lytic induction. GAPDH was used as a housekeeper (n=3).

(E) qPCR analysis of *CCNB1* expression in DLL4 KD cell lines transfected with either a scrambled control or miR-30c mimic 24 hours prior to lytic induction. GAPDH was used as a housekeeper (n=3).

(F) qPCR analysis of *CCNE1* expression in DLL4 KD cell lines transfected with either a scrambled control or miR-30c mimic 24 hours prior to lytic induction. GAPDH was used as a housekeeper (n=3).

(G) Representative western blot of scr or miR-30c transfected DLL4 KD TREx cells analysed for ORF65 expression at 0, 24 and 48 hours post lytic induction with GAPDH as a loading control. (n=3).

Data information: In A-F data are presented as mean ± SD. *P<0.05, **P<0.01, ***P<0.001 (Unpaired Student's t-test). All repeats are biological.

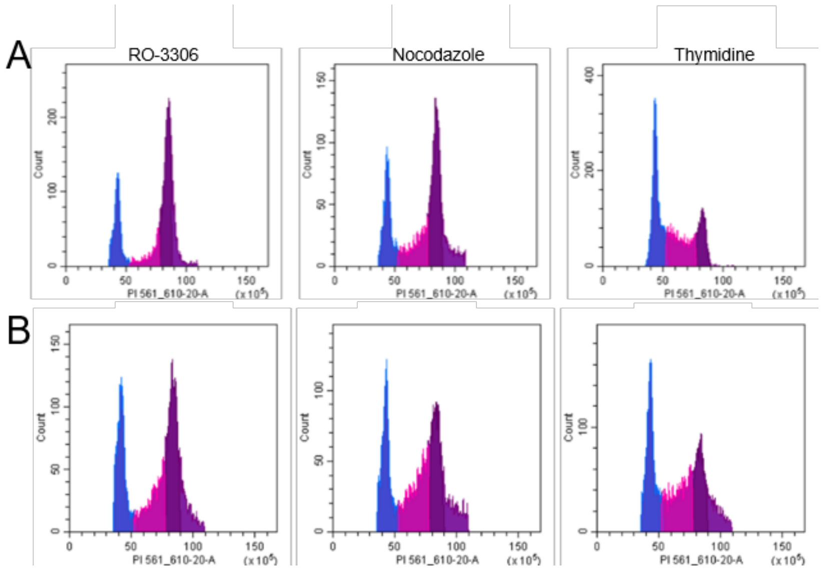

Appendix Figure S7:

(A-B) Representative plots of cell cycle distribution at 0 **(A)** and 24 **(B)** hours post induction in RO-3306, nocodazole and thymidine treated TREx cells, with G1 phase (blue), S phase (pink) and G2/M phase (purple) highlighted.

Primer Name	Sequence
GAPDH Forward	TGTCAGTGGTGGACCTGA
GAPDH Reverse	GTGGTCGTTGAGGGCAATG
circHIPK3 Forward	TATGTTGGTGGATCCTGTTCGGCA
circHIPK3 Reverse	TGGTGGGTAGACCAAGAGTGGTGA
ORF57 Forward	GCCATAATCAAGCGTACTGG
ORF57 Reverse	GCAGAGAAATATTGCGGTGT
DLL4 Forward	CCCTGGCAATGTACTTGTGAT
DLL4 Reverse	TGGTGGGTGCAGTAGTTG
Pri-miR-29b Forward	TGAAAGCAACAGCAGGATGG
Pri-miR-29b Reverse	ACCCAAGACAACCTAGAAAGGA
Pre-miR-29b Forward	TTGCCACTTGAGTCTGTT
Pre-miR-29b Reverse	CTTCTCTACTGTCACCTCTC
Pri-miR-30c Forward	CTGATCAACCCTGGACCCTG
Pri-miR-30c Reverse	GGTCGCATTCTGTCCGATCT
Pre-miR-30c Forward	GTGTAAACATCCTACACTCTCAGC
Pre-miR-30c Reverse	TGGCAGAAGGAGTAAACAACCC
Linear *HIPK3* Forward	GGCATCAAGAGTGGAATGGA
Linear *HIPK3* Reverse	TGATGAATGGTTGGGGATGG
Pre-BTG1 Forward	GTCAACGGCACAATTAACAG
Pre-BTG1 Reverse	TGCACACAATGGAGTTGATG
CCNB1 Forward	CATGGTGCACTTTCCTCCTT
CCNB1 Reverse	AGGTAATGTTGTAGAGTTGGTGTCC
CCNE1 Forward	CCACACCTGACAAAGAAGATCATCAC
CCNE1 Reverse	GAGCCTCTGGATGGTGCAATA
DLL4 KD 1 siRNA	GCAAGAAGCGCAATGACCACT
DLL4 KD 2 siRNA	GCAGGGAAGCCATGAACAACT
circHIPK3 KD siRNA	CTACAGGTATGGCCTCACA

Appendix Table S1:

Primer and siRNAs Sequences
