# Supplementary material for: Dysregulation of the miR‐30c/DLL4 axis by circHIPK3 is essential for KSHV lytic replication
Source: EMBO Rep. 2022 Mar 3;23(5):e54117. doi: 10.15252/embr.202154117 (PMC9066072; doi:10.15252/embr.202154117)
Supplement: Supplementary file 2 — Source Data for Appendix [file EMBR-23-e54117-s005.zip › Appendix_Figure_Source_Data/EMBOR-2021-54117V2-Appendix_Figure_S5_Source_Data-sd.pdf]

Supplementary 5

D

0	24
1	1.82134
1	1.892115
1	1.993081

E

0	24
1	0.484645
1	0.418994
1	0.668964

F

0	24
1	8.196455
1	8.784743
1	4.873641

G

0	24
1	2.114036
1	2.234574
1	3.116658

H

0	24
1	6.020987
1	7.94474
1	5.856343
