# Supplementary material for: Dysregulation of the miR‐30c/DLL4 axis by circHIPK3 is essential for KSHV lytic replication
Source: EMBO Rep. 2022 Mar 3;23(5):e54117. doi: 10.15252/embr.202154117 (PMC9066072; doi:10.15252/embr.202154117)

Supplementary 6

A

	Scr			circHIPK3 GapmeR		
0	1	1	1	0.514057	0.628507	0.346277
24	2.121375	3.182492	2.250361	0.217638	0.441351	0.239816

B

	Scr			circHIPK3 GapmeR		
0	1	1	1	0.952638	0.931688	1.115353
24	0.879649	0.846745	1.168804	1.152686	0.804926	1.038859

C

	Scr			Scr + circHIPK3 KD			DLL4 KD			DLL4 KD + circHIPK3 KD		
0	1	1	1	1.028114	0.840896	0.858565	0.936272	0.962594	1.05277	0.962594	0.817902	1.337928
24	0.770551	0.720742	0.817902	1.489677	1.136817	1.093952	1.205808	1.547565	1.36604	1.333299	1.500039	1.409321

D

	Scr			Scr + circHIPK3 KD			DLL4 KD			DLL4 KD + circHIPK3 KD		
0	1	1	1	0.972655	0.895025	1.274561	0.870551	0.849685		0.999062	1.414214	1.035265
24	0.273573	0.115023	0.304721	0.492769	0.657471	0.51943	0.635262	0.435275	0.699793	0.555555	0.413225	0.592546

E

	Scr			miR-30c		
0	1	1	1	1.010451	1.193336	0.946058
24	0.936272	0.849685	1.164734	1.278986	1.529074	0.888843

F

	Scr			miR-30c		
0	1	1	1	0.965936	0.858565	0.907519
24	0.576343	0.360982	0.373712	0.846745	0.690159	0.45376

G

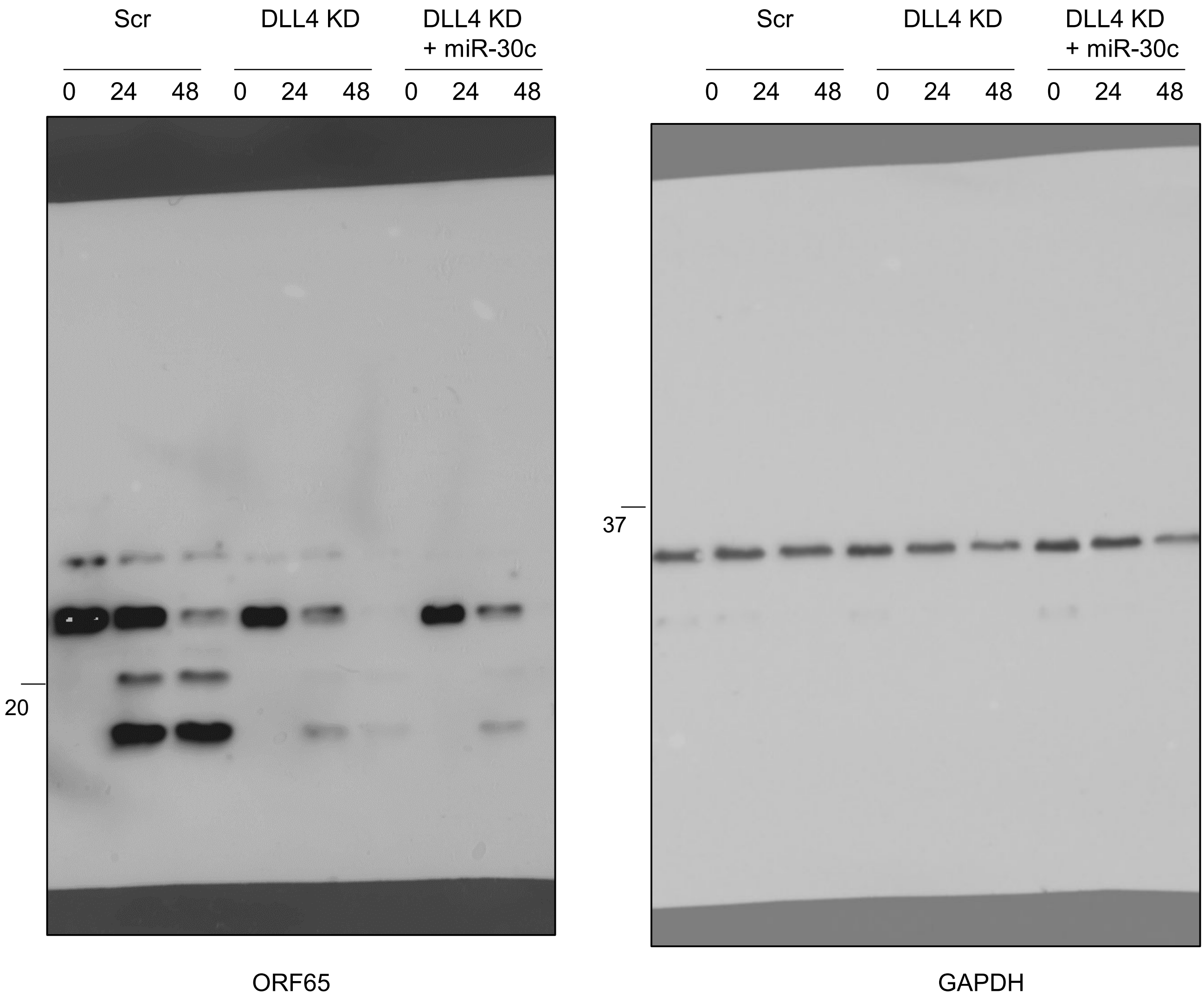

Supplement: Supplementary file 2 — Source Data for Appendix [file EMBR-23-e54117-s005.zip › Appendix_Figure_Source_Data/EMBOR-2021-54117V2-Appendix_Figure_S6_Source_Data-sd.pdf]
