# Supplementary figures and images for: Dysregulation of the miR‐30c/DLL4 axis by circHIPK3 is essential for KSHV lytic replication
Source: EMBO Rep. 2022 Mar 3;23(5):e54117. doi: 10.15252/embr.202154117 (PMC9066072; doi:10.15252/embr.202154117)

Supplementary 1

H

| 0 | 24       |
|---|----------|
| 1 | 0.926588 |
| 1 | 1.189207 |
| 1 | 1.347234 |

I

|         | 0        |          |   | 24       |          |   | 48       |          |   |
|---------|----------|----------|---|----------|----------|---|----------|----------|---|
|         | Mean     | SD       | N | Mean     | SD       | N | Mean     | SD       | N |
| scr     | 1        | 0        | 3 | 0.25437  | 0.070732 | 3 | 0.081616 | 0.046185 | 3 |
| miR-30c | 115.0587 | 13.30399 | 3 | 78.99156 | 8.185419 | 3 | 39.85568 | 7.423592 | 3 |

J

|    | Scr      |          |          | miR-30c AntagomiR |          |          |
|----|----------|----------|----------|-------------------|----------|----------|
| 0  | 1        | 1        | 1        | 0.461691          | 0.393654 | 0.322401 |
| 24 | 0.384219 | 0.275476 | 0.374212 | 0.12421           | 0.140146 | 0.135372 |

K

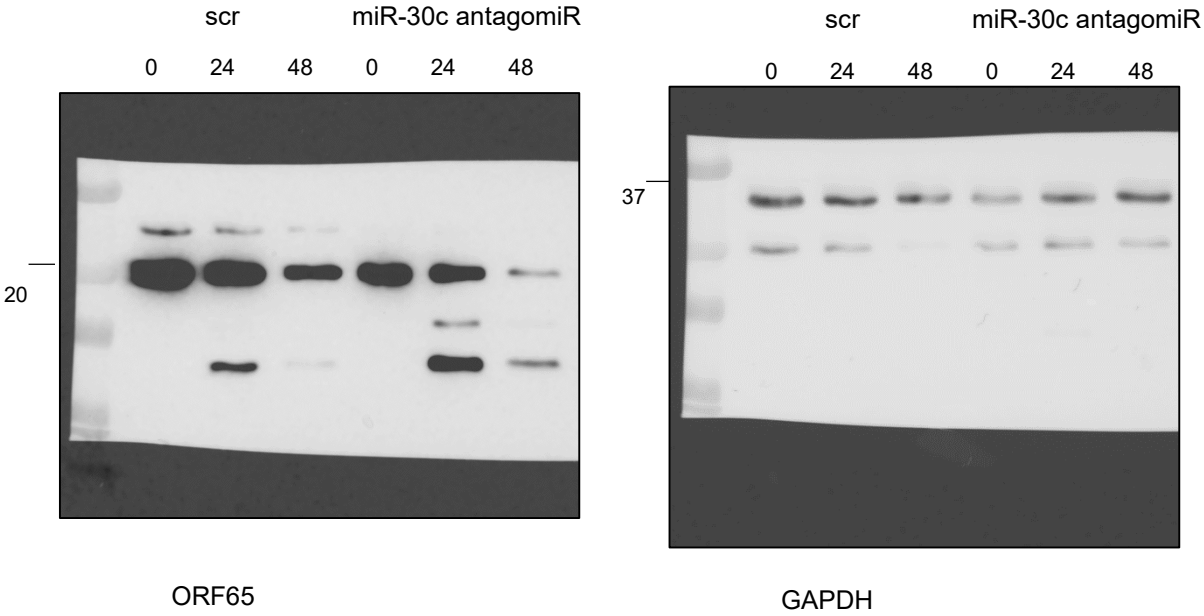

Supplement: Supplementary file 2 — Source Data for Appendix [file EMBR-23-e54117-s005.zip › Appendix_Figure_Source_Data/EMBOR-2021-54117V2-Appendix_Figure_S1_Source_Data-sd.pdf]

Supplementary 3

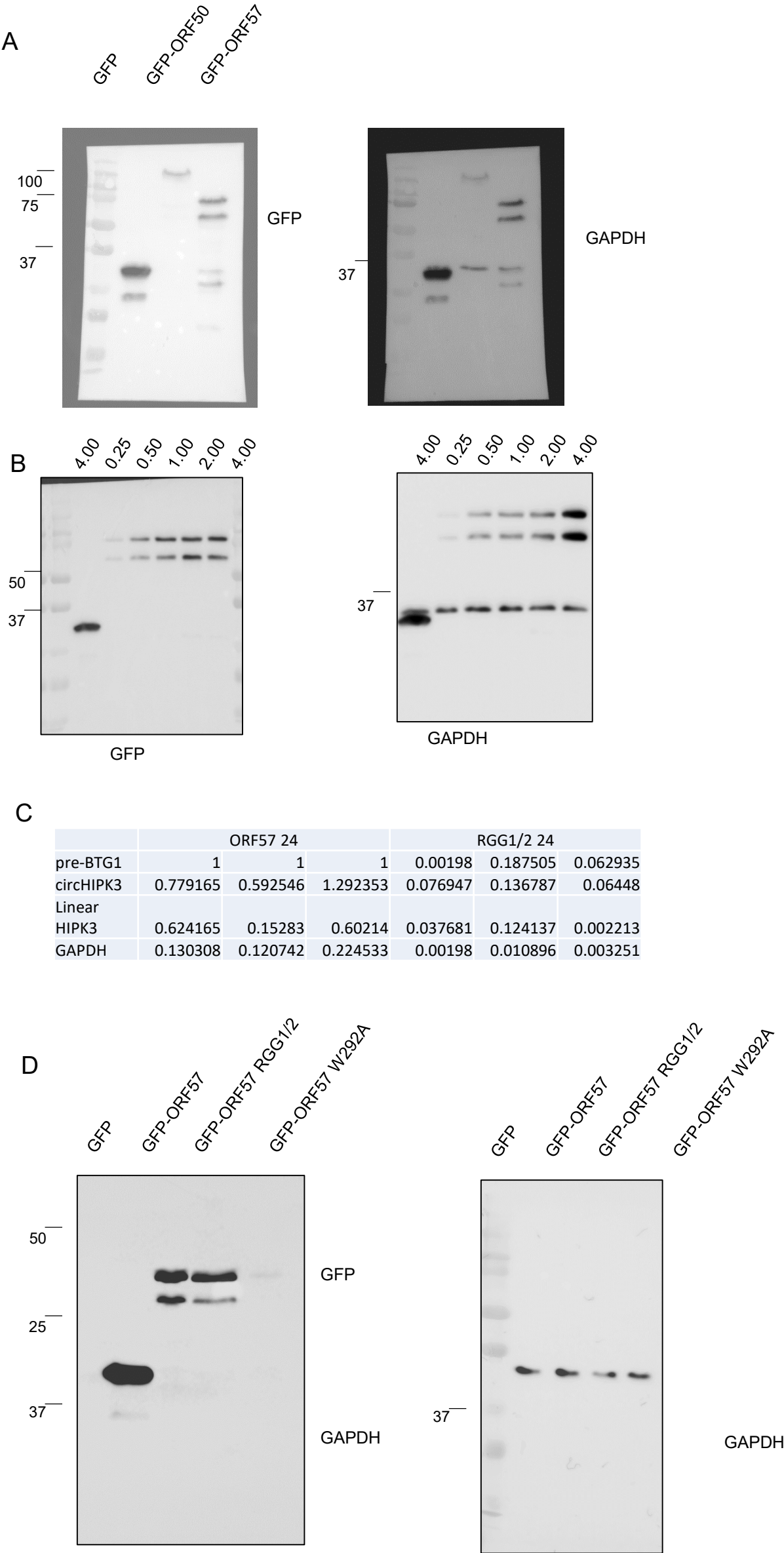

Supplement: Supplementary file 2 — Source Data for Appendix [file EMBR-23-e54117-s005.zip › Appendix_Figure_Source_Data/EMBOR-2021-54117V2-Appendix_Figure_S3_Source_Data-sd.pdf]

Figure 5

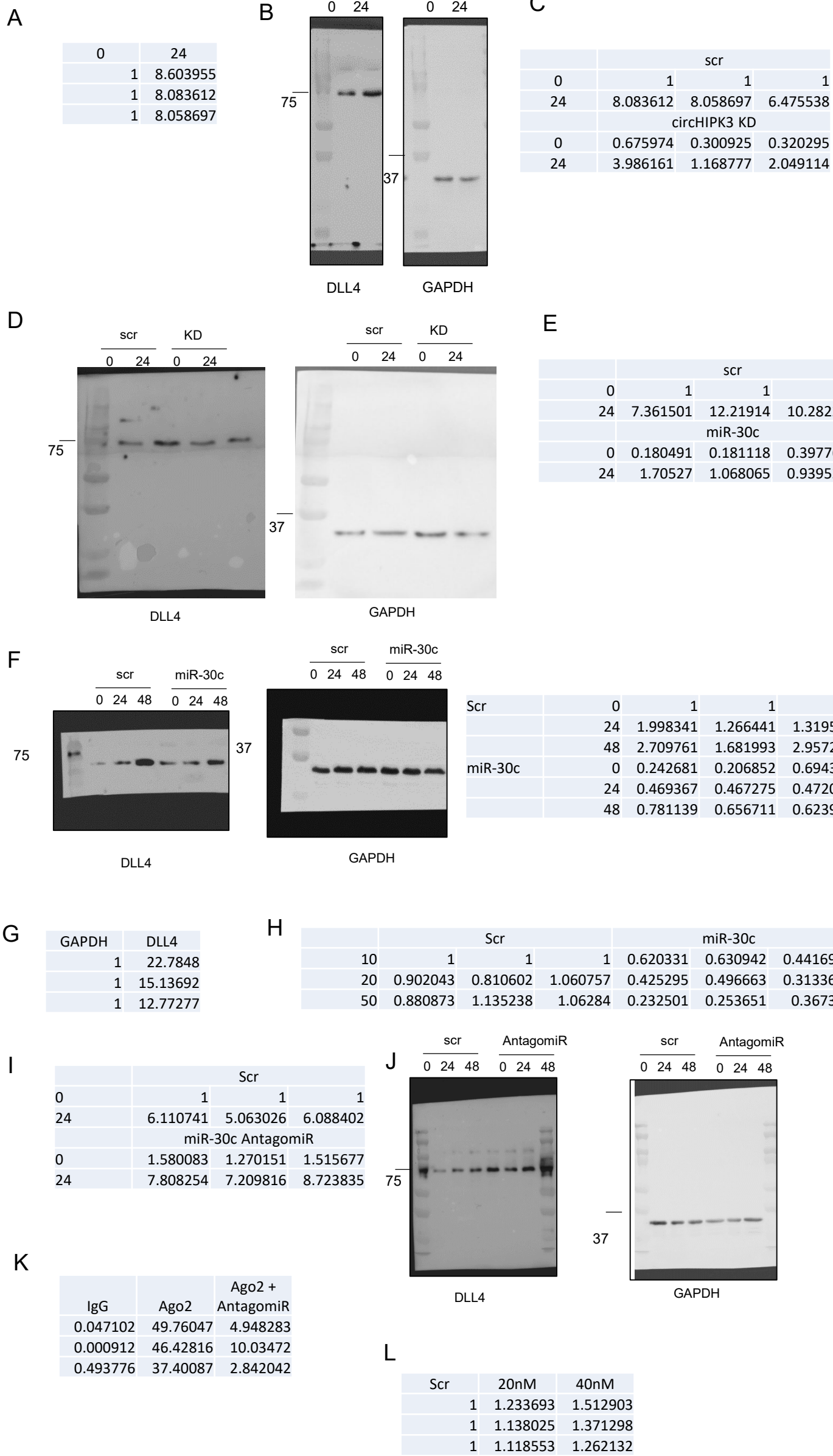

Supplement: Supplementary file 7 — Source Data for Figure 5 [file EMBR-23-e54117-s007.pdf]

Figure 6

A

|    | Scr      |         |          | KD 1     |          |          | KD 2     |          |          |
|----|----------|---------|----------|----------|----------|----------|----------|----------|----------|
| 0  | 1        | 1       | 1        | 0.382889 | 0.840896 | 0.343885 | 1.647182 | 0.680657 | 0.558644 |
| 24 | 6.453134 | 6.84476 | 6.892369 | 2.557971 | 2.505329 | 2.989698 | 2.378414 | 2.020903 | 3.784231 |

B

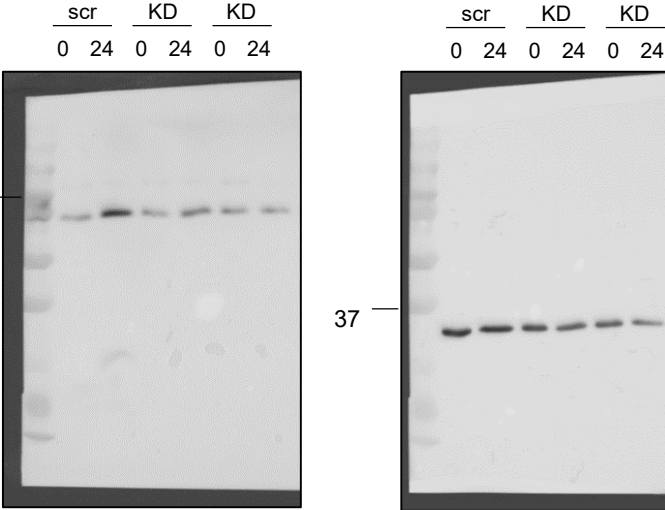

DLL4

GAPDH

C

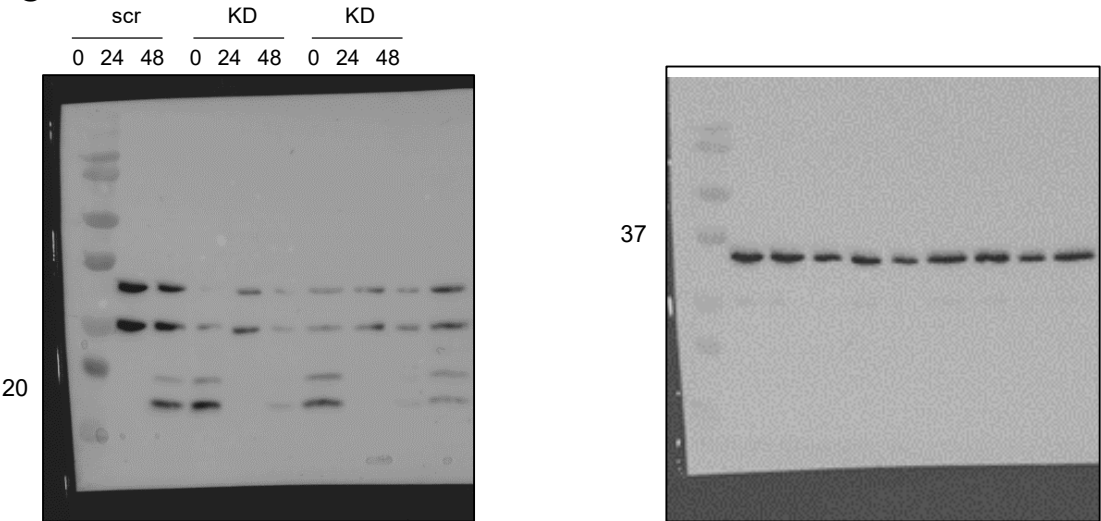

ORF65

GAPDH

D

| Latent   | scr | DLL4 KD 1 | DLL4 KD 2 |
|----------|-----|-----------|-----------|
| 0.167822 | 1   | 0.447513  | 0.510506  |
| 0.065607 | 1   | 0.493116  | 0.78187   |
| 0.095061 | 1   | 0.351111  | 0.543367  |

E

| Latent   | scr | DLL4 KD 1 | DLL4 KD 2 |
|----------|-----|-----------|-----------|
| 0.015843 | 1   | 0.099787  | 0.21299   |
| 0.017444 | 1   | 0.100134  | 0.451831  |
| 0.122428 | 1   | 0.093354  | 0.099787  |

Supplement: Supplementary file 8 — Source Data for Figure 6 [file EMBR-23-e54117-s003.pdf]
