# Supplementary material for: Dysregulation of the miR‐30c/DLL4 axis by circHIPK3 is essential for KSHV lytic replication
Source: EMBO Rep. 2022 Mar 3;23(5):e54117. doi: 10.15252/embr.202154117 (PMC9066072; doi:10.15252/embr.202154117)
Supplement: Supplementary file 3 — Source Data for Figure 1 [file EMBR-23-e54117-s006.pdf]

Figure 1

D

	miR-29b		miR-30c	
	0hr	24hr	0hr	24hr
Repeat 1	1	0.175556	1	0.285191
Repeat 2	1	0.267016	1	0.354781
Repeat 3	1	0.243164	1	0.213324

E

	Pri-miR-29b		Pri-miR-30c	
	0hr	24hr	0hr	24hr
Repeat 1	1	1.71119	1	0.815072
Repeat 2	1	1.287882	1	1.128964
Repeat 3	1	1.205808	1	0.707107

F

	Pre-miR-29b		Pre-miR-30c	
	0hr	24hr	0hr	24hr
Repeat 1	1	1.328686	1	0.80107
Repeat 2	1	1.010451	1	1.347234
Repeat 3	1	1.552938	1	1.827663

G

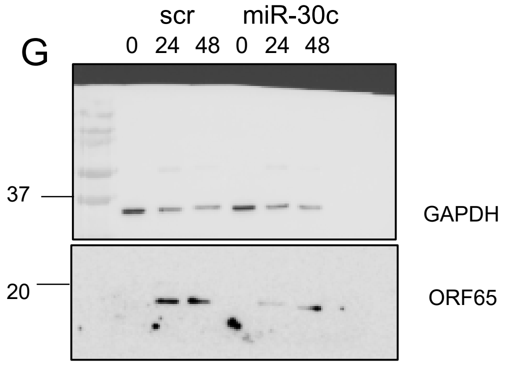

H

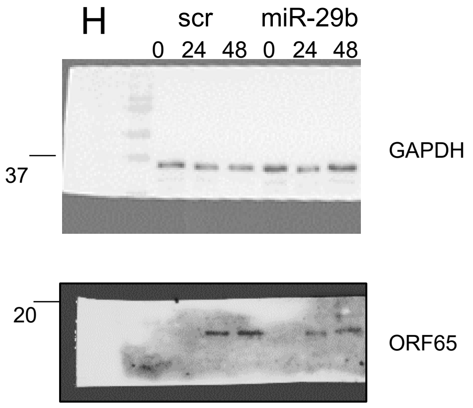

I

Latent	scr	miR-30c
0.081334	1	0.30566
0.07668	1	0.279322
0.076947	1	0.222211

J

Latent	scr	miR-30c
0.053034	1	0.305668
0.063728	1	0.588469
0.062633	1	0.260623
