# Supplementary material for: Dysregulation of the miR‐30c/DLL4 axis by circHIPK3 is essential for KSHV lytic replication
Source: EMBO Rep. 2022 Mar 3;23(5):e54117. doi: 10.15252/embr.202154117 (PMC9066072; doi:10.15252/embr.202154117)
Supplement: Supplementary file 4 — Source Data for Figure 2 [file EMBR-23-e54117-s002.pdf]

Figure 2

A

0hr	24hr
1	3.24901
1	2.685145
1	2.741566

B

0hr	24hr
1	0.939523
1	0.972655
1	1.028114

D

IgG scr	Ago2 scr	Ago2 miR-29b	Ago2 miR-30c	IgG scr	Ago2 scr	Ago2 + AntagomiR
1	5.105061	17.9005	15.52938	1	7.684376	3.07375
1	10.75494	6.04997	34.2238	1	13.94744	2.114036
1	8.321987	11.48698	22.81527	1	9.598814	3.077861

E

miR-29b		miR-30c	
GAPDH	circHIPK3	GAPDH	circHIPK3
0.389582	1	0.378931	1
0.562529	1	0.299401	1
0.087171	1	0.420168	1
0.006615	1	0.241546	1
