# Supplementary material for: Dysregulation of the miR‐30c/DLL4 axis by circHIPK3 is essential for KSHV lytic replication
Source: EMBO Rep. 2022 Mar 3;23(5):e54117. doi: 10.15252/embr.202154117 (PMC9066072; doi:10.15252/embr.202154117)
Supplement: Supplementary file 5 — Source Data for Figure 3 [file EMBR-23-e54117-s004.pdf]

Figure 3

A

scr		KD	
0	24	0	24
1	4.924578	0.806642	0.949342
1	3.375263	0.413225	1.010451
1	4.96	0.898132	0.803851

B

scr		KD	
0	24	0	24
1	1.075494	1.136817	1.156688
1	0.768438	1.035265	0.962594
1	1.239708	1.558329	0.933033

C

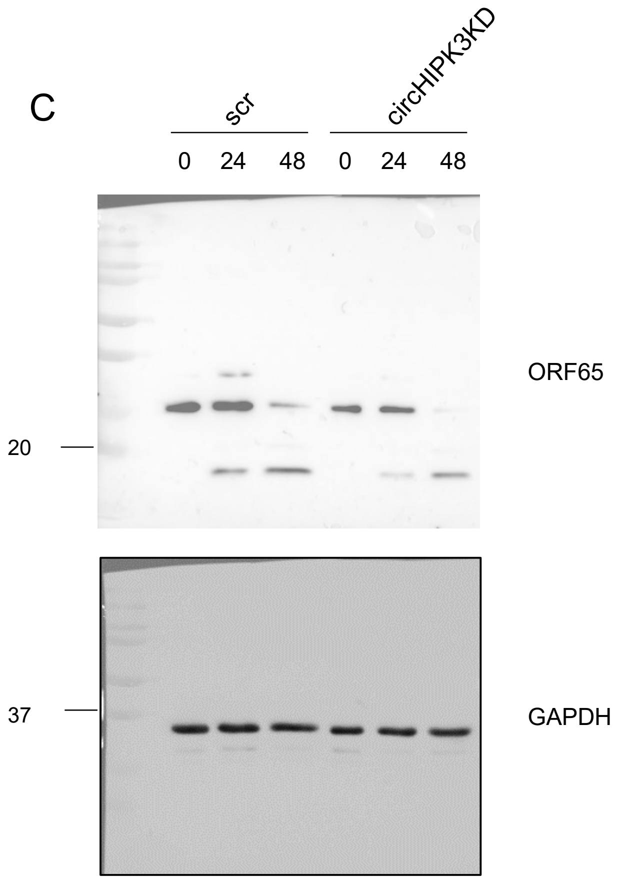

D

Latent	Scr	circHIPK3 KD
0.143091	1	0.455335
0.060162	1	0.596668
0.119493	1	0.430773

E

Latent	Scr	circHIPK3 KD
0.03901	1	0.381565
0.006872	1	0.151774
0.100481	1	0.380245

F

		miR-29b			miR-30c			miR-27a		
	0	1	1	1	1	1	1	1	1	1
Scr	24	0.380245	0.263401	0.225313	0.598739	0.379436	0.287175	0.231647	0.486327	0.236514
	0	1.520979	1.563739	1.031683	0.913831	1.351911	1.328686	0.913831	0.926588	0.96929
KD	24	1.248331	1.585568	0.885768	1.765406	1.328686	1.494849	0.158769	0.145088	0.325825
