# Supplementary material for: Dysregulation of the miR‐30c/DLL4 axis by circHIPK3 is essential for KSHV lytic replication
Source: EMBO Rep. 2022 Mar 3;23(5):e54117. doi: 10.15252/embr.202154117 (PMC9066072; doi:10.15252/embr.202154117)
Supplement: Supplementary file 6 — Source Data for Figure 4 [file EMBR-23-e54117-s001.pdf]

Figure 4

A

0	16	24	48	72
1	0.913831	3.193194	3.148929	4.053031
1	1.094294	3.105876	3.377526	3.815747
1	0.852635	2.908879	3.052598	4.292733

B

0	16	24	48	72
1	0.91067	0.972655	0.721965	0.628507
1	0.96929	1.082975	0.755236	0.191445
1	0.835088	0.939523	0.586417	0.535887

C

GFP	GFP-ORF50	GFP-ORF57	RGG1/2
1	0.959264	4.890561	2.703822
1	0.598739	4.789915	2.602684
1	1.261377	4.469149	2.281528

D

GFP	GFP-ORF50	GFP-ORF57	RGG1/2
1	1.172835	0.639493	1.049717
1	0.939523	0.765779	1.012804
1	0.965936	0.463294	0.750019

E

GFP	GFP-ORF57				
4.00	0.25	0.50	1.00	2.00	4.00
1	1.607702	2.353813	2.639016	3.294364	3.07375
1	1.45902	1.624505	1.965641	1.808759	1.693491
1	1.735077	2.181015	2.639016	3.182146	2.907945

D

	GFP 16			GFP 24			GFP-ORF57 16			GFP-ORF57 24		
	Mean	SD	N	Mean	SD	N	Mean	SD	N	Mean	SD	N
pre-BTG1	0.00467	0.007424	3	0.140315	0.127805	3	1	0	3	1	0	3
circHIPK3	0.087295	0.089496	3	0.069782	0.071603	3	0.912625	0.188741	3	0.651054	0.082742	3
Linear HIPK3	0.006407	0.008249	3	0.078792	0.147203	3	1.016981	0.359196	3	0.193854	0.140109	3
GAPDH	0.083626	0.057282	3	0.059233	0.044853	3	0.159921	0.021251	3	0.150347	0.076598	3
