# Supplementary material for: Dysregulation of the miR‐30c/DLL4 axis by circHIPK3 is essential for KSHV lytic replication
Source: EMBO Rep. 2022 Mar 3;23(5):e54117. doi: 10.15252/embr.202154117 (PMC9066072; doi:10.15252/embr.202154117)
Supplement: Supplementary file 9 — Source Data for Figure 7 [file EMBR-23-e54117-s009.pdf]

Figure 7

A

	Scr			circHIPK3 KD			DLL4 KD		
0	1	1	1	0.888843	1.333299	1.552938	1.075494	1.126733	1.082975
24	0.773782	0.68302	0.817902	1.226885	1.205808	0.972655	1.164734	1.414214	0.91067

B

	Scr			circHIPK3 KD			DLL4 KD		
0	1	1	1	0.989657	1.193336	1	0.812252	0.993092	0.986233
24	0.25349	0.243164	0.273573	0.427798	0.724471	0.41466	0.372419	0.369847	0.382889

C

	scr			circHIPK3 KD			DLL4 KD		
	Mean	SD	N	Mean	SD	N	Mean	SD	N
G1	1.14023	0.171075	3	0.990723	0.178196	3	1.152885	0.125927	3
S	0.938608	0.291243	3	1.014528	0.120517	3	0.881231	0.152468	3
G2	0.586319	0.073607	3	0.895598	0.1304	3	0.795677	0.076647	3

D

	scr			circHIPK3 KD			DLL4 KD		
	Mean	SD	N	Mean	SD	N	Mean	SD	N
G1	0.986391	0.19524	3	0.923999	0.082129	3	1.034594	0.077817	3
S	1.030178	0.313554	3	1.054191	0.180973	3	0.975614	0.255266	3
G2	0.577952	0.001185	3	0.841509	0.03356	3	0.853928	0.146794	3

E

	Untreated			RO 3306			Nocodazole			Thymidine		
0	0.064704	0.098755	0.06527	0.075887	0.103665	0.091823	0.116226	0.066293	0.089312	0.085971	0.135842	0.07966
24	1	1	1	0.655197	0.421908	0.626332	0.556711	0.539614	0.523042	1.013959	1.270151	1.193336

F

	Scr			Scr + thymidine			DLL4 KD			DLL4 KD + thymidine		
0	0.064704	0.098755	0.06527	0.089003	0.085971	0.135842	0.116226	0.066293	0.123279	0.085971	0.135842	0.067218
24	1	1	1	0.832199	1.013959	1.270151	0.390935	0.267016	0.515842	0.82932	0.752623	0.803942
