# Supplementary material for: Dysregulation of the miR‐30c/DLL4 axis by circHIPK3 is essential for KSHV lytic replication
Source: EMBO Rep. 2022 Mar 3;23(5):e54117. doi: 10.15252/embr.202154117 (PMC9066072; doi:10.15252/embr.202154117)
Supplement: Supplementary file 10 [file EMBR-23-e54117-s010.pdf]

EMBO reports

Dysregulation of the miR-30c/DLL4 axis by circHIPK3 is essential for KSHV lytic replication

Katherine Harper, Timothy Mottram, Chinedu Anene, Becky Foster, Molly Patterson, Euan McDonnell, Andrew Macdonald, David R. Westhead, and Adrian Whitehouse
DOI: 10.15252/embr.202154117

Corresponding author(s): Adrian Whitehouse (a.whitehouse@leeds.ac.uk) , Katherine Harper (bs14klh@leeds.ac.uk)

Review Timeline:

Submission Date:	7th Oct 21
Editorial Decision:	22nd Oct 21
Revision Received:	14th Jan 22
Editorial Decision:	28th Jan 22
Revision Received:	15th Feb 22
Accepted:	18th Feb 22

Editor: Achim Breiling

Transaction Report:

(Note: With the exception of the correction of typographical or spelling errors that could be a source of ambiguity, letters and reports are not edited. Depending on transfer agreements, referee reports obtained elsewhere may or may not be included in this compilation. Referee reports are anonymous unless the Referee chooses to sign their reports.)

1st Editorial Decision **22nd Oct 2021**

Dear Dr. Whitehouse,

Thank you for the submission of your research manuscript to EMBO reports. We have now received the reports from the three referees that were asked to evaluate your study, which can be found at the end of this email.

As you will see, the referees think that these findings are of interest. However, they have several comments, concerns and suggestions, indicating that a major revision of the manuscript is necessary to allow publication of the study in EMBO reports. As the reports are below, and all their points need to be addressed, I will not detail them here.

Given the constructive referee comments, we would like to invite you to revise your manuscript with the understanding that all referee concerns must be addressed in the revised manuscript or in the detailed point-by-point response. Acceptance of your manuscript will depend on a positive outcome of a second round of review. It is EMBO reports policy to allow a single round of revision only and acceptance of the manuscript will therefore depend on the completeness of your responses included in the next, final version of the manuscript.

Revised manuscripts should be submitted within three months of a request for revision. Please contact me to discuss the revision should you need additional time.

When submitting your revised manuscript, please also carefully review the instructions that follow below.

PLEASE NOTE THAT upon resubmission revised manuscripts are subjected to an initial quality control prior to exposition to re-review. Upon failure in the initial quality control, the manuscripts are sent back to the authors, which may lead to delays. Frequent reasons for such a failure are the lack of the data availability section (please see below) and the presence of statistics based on n=2 (the authors are then asked to present scatter plots or provide more data points).

When submitting your revised manuscript, we will require:

1) a .docx formatted version of the final manuscript text (including legends for main figures, EV figures and tables), but without the figures included. Please make sure that changes are highlighted to be clearly visible. Figure legends should be compiled at the end of the manuscript text.

2) individual production quality figure files as .eps, .tif, .jpg (one file per figure), of main figures and EV figures. Please upload these as separate, individual files upon re-submission.

The Expanded View format, which will be displayed in the main HTML of the paper in a collapsible format, has replaced the Supplementary information. You can submit up to 5 images as Expanded View. Please follow the nomenclature Figure EV1, Figure EV2 etc. The figure legend for these should be included in the main manuscript document file in a section called Expanded View Figure Legends after the main Figure Legends section. Additional Supplementary material should be supplied as a single pdf file labeled Appendix. The Appendix should have page numbers and needs to include a table of content on the first page (with page numbers) and legends for all content. Please follow the nomenclature Appendix Figure Sx, Appendix Table Sx etc. throughout the text, and also label the figures and tables according to this nomenclature.

For more details, please refer to our guide to authors:
http://www.embopress.org/page/journal/14693178/authorguide#manuscriptpreparation

Please consult our guide for figure preparation:
http://wol-prod-cdn.literatumonline.com/pb-assets/embo-site/EMBOPress_Figure_Guidelines_061115-1561436025777.pdf

See also the guidelines for figure legend preparation:
https://www.embopress.org/page/journal/14693178/authorguide#figureformat

3) a .docx formatted letter INCLUDING the reviewers' reports and your detailed point-by-point responses to their comments. As part of the EMBO Press transparent editorial process, the point-by-point response is part of the Review Process File (RPF), which will be published alongside your paper.

4) a complete author checklist, which you can download from our author guidelines (https://www.embopress.org/page/journal/14693178/authorguide). Please insert page numbers in the checklist to indicate where the requested information can be found in the manuscript. The completed author checklist will also be part of the RPF.

Please also follow our guidelines for the use of living organisms, and the respective reporting guidelines:
http://www.embopress.org/page/journal/14693178/authorguide#livingorganisms

5) that primary datasets produced in this study (e.g. RNA-seq, ChIP-seq, structural and array data) are deposited in an appropriate public database. If no primary datasets have been deposited, please also state this a dedicated section (e.g. 'No primary datasets have been generated and deposited'), see below.

See also: http://embor.embopress.org/authorguide#datadeposition

Please remember to provide a reviewer password if the datasets are not yet public.

The accession numbers and database should be listed in a formal "Data Availability " section (placed after Materials & Methods) that follows the model below. This is now mandatory (like the COI statement). Please note that the Data Availability Section is restricted to new primary data that are part of this study.

Data availability

The datasets produced in this study are available in the following databases:

- RNA-Seq data: Gene Expression Omnibus GSE46843 (https://www.ncbi.nlm.nih.gov/geo/query/acc.cgi?acc=GSE46843)
- [data type]: [name of the resource] [accession number/identifier/doi] ([URL or identifiers.org/DATABASE:ACCESSION])

*** Note - All links should resolve to a page where the data can be accessed. ***

Moreover, I have these editorial requests:

6) We strongly encourage the publication of original source data with the aim of making primary data more accessible and transparent to the reader. The source data will be published in a separate source data file online along with the accepted manuscript and will be linked to the relevant figure. If you would like to use this opportunity, please submit the source data (for example scans of entire gels or blots, data points of graphs in an excel sheet, additional images, etc.) of your key experiments together with the revised manuscript. If you want to provide source data, please include size markers for scans of entire gels, label the scans with figure and panel number, and send one PDF file per figure.

7) Our journal encourages inclusion of *data citations in the reference list* to directly cite datasets that were re-used and obtained from public databases. Data citations in the article text are distinct from normal bibliographical citations and should directly link to the database records from which the data can be accessed. In the main text, data citations are formatted as follows: "Data ref: Smith et al, 2001" or "Data ref: NCBI Sequence Read Archive PRJNA342805, 2017". In the Reference list, data citations must be labeled with "[DATASET]". A data reference must provide the database name, accession number/identifiers and a resolvable link to the landing page from which the data can be accessed at the end of the reference. Further instructions are available at: http://www.embopress.org/page/journal/14693178/authorguide#referencesformat

8) Regarding data quantification and statistics, can you please specify, where applicable, the number "n" for how many independent experiments were performed, if these were biological or technical replicates, the bars and error bars (e.g. SEM, SD) and the test used to calculate p-values in the respective figure legends. Please provide statistical testing where applicable, and also add a paragraph detailing this to the methods section. See:
http://www.embopress.org/page/journal/14693178/authorguide#statisticalanalysis

9) Please also note our reference format:
http://www.embopress.org/page/journal/14693178/authorguide#referencesformat

10) Please add scale bars of similar style and thickness to all the microscopic images, using clearly visible black or white bars (depending on the background). Please place these in the lower right corner of the images. Please do not write on or near the bars in the image but define the size in the respective figure legend.

I look forward to seeing a revised version of your manuscript when it is ready. Please let me know if you have questions or comments regarding the revision.

Please use this link to submit your revision: https://embor.msubmit.net/cgi-bin/main.plex

Yours sincerely,

Achim Breiling
Editor
EMBO Reports

Referee #1:

This is an interesting manuscript that will likely appeal to RNA biologists and virologists. Some of the key findings include: KSHV upregulates circHIPK3 in B cells, circHIPK3 competes with miR-30c resulting in upregulation of DLL4, and disruption of ncRNA network has negative impact on KSHV lytic replication. Overall, most of the conclusions are supported, but experimental and analysis details need to be expanded. Issues with proper controls were present in some assays.

1. Fig. 1G, H: GAPDH is a target of host shutoff, so this is not the best loading control for immunoblots. Fig. 3C: Why is GAPDH reduced in Fig. 1G,H in induced conditions, but not in induced conditions in Fig. 3C?
2. While authors performed RIP on ORF57 and an ORF57-RGG (Fig S6) mutant showing that the RNA binding mutant did not bind transcripts, authors did not show whether transfection of ORF57-RGG mutant still results in circHIPK3 increased expression. This is a critical experiment to support their conclusion that ORF57 enhances circHIPK3 expression.
3. Authors need to expand on details of analysis and results for miRNA-Seq dataset. The methods section was sparse and SRA/GEO accession numbers were not provided. Additional figures should be generated to support their claim at line 113 "with the vast majority downregulated during lytic replication in B cells."
4. Fig. 2C: Labels are needed on the figure. FISH typically is not used for BSJs because a single probe is usually not sensitive enough. Please include negative controls and show fields of cells, not a single cell.
5. Fig. 4: Showing the circHIPK3 to linear HIPK3 ratios would be helpful to assess how these ratios might be changing at these different timepoints.
6. In conventional ceRNA theory, miRNAs will bind to ceRNAs but not actively degraded. How do you explain there is a reduction of miR-30 with upregulated circHIPK3?
7. Current evidence is not enough to claim ORF57's function in promoting circularization. Do you see the reduction of linear HIPK3 upon overexpression of circHIPK3?
8. DLL4 is just one of miR-30's targets. What about other targets? Since you have done RNA-Seq (Fig. S8), you should be able to check expression changes of other targets. For example, you can separate mRNAs with miR-30 seed match and mRNAs without, and perform a Kolmogorov-Smirnov test.

Referee #2:

This is a very interesting study describing a non-coding RNA network driving KSHV lytic reactivation. In general, this is a well-designed and performed study. However, there are some key points that need to be addressed before the manuscript can be acceptable for publication in EMBO Reports.
Main points:
1) The authors propose a ceRNA network, which would be more accurately described as a circular RNA acting as a sponge. A critical point around any such network is the question of abundance (Denzler et al., Molecular Cell, 2014, PMID: 24793693). Is it possible circHIPK3 having 3 and 2 sites for miR-29b and miR-30c sequesters enough of the two miRNAs to de-repress DLL4 expression? The authors should consider this and determine absolute copy number of circHIPK3, miR-29b, miR-30c, DDL4 mRNA in latent and lytic cells. This would substantially strengthen the evidence supporting the existence of the proposed network.
2) Experimentally, the authors use extensively over-expression of miR-29b and miR-30c. These results in non-physiologically relevant copy numbers of the two mature miRNAs. Experiments where the two miRNAs are inhibited should be performed to confirm that the effects are observed with the endogenous miRNAs.
3) A critical experiment that would validate the existence of the circHIPK3/miR-30c/DDL4 network is missing: the authors should test whether circHIPK3 knockdown or miR-30c over-expression/knockdown have any effects on lytic replication and cell cycle genes in DLL4knockdown cells. I appreciate these are complex experiments, but they would demonstrate that circHIPK3 and miR-30c exert their effects on the lytic through DDL4.
4) All experiments in the manuscript are performed in one KSHV-infected B cell line. It would be useful to validate some of the key findings in another KSHV-positive PEL cell line. For example, regulation of miR-30c and circHIPK3 and the effect of their manipulation on the lytic switch.
5) The section on the role of ORF57 in circHIPK3 induction is interesting but incomplete. Do the authors imply that ORF57 contributes to generation (back-splicing) or stability of the circRNA? This needs to be further investigated or removed.
6) A section on statistics should be added to materials and methods.

Minor points:
1) Line 62-63: Please add some references on ceRNAs.
2) Line 114-115/Fig 1a: Please explain more what you mean by "Based on a combination of known function, abundance and Z-score, miR-30c and miR-29b were selected for further investigation". There is a long list of miRNAs in Fig1A.
3) Line 117/Fig 1b/c: interesting to see this miRNA regulation in KS biopsies. However, these are predominantly comprised of latently infected cells. How does this fit with the lytic switch?
4) Line 149: How were the RNA-binding predictions performed? Was it just alignment?
5) Fig 2d: This is a prime example where inhibition of miRNAs should be considered in addition to over-expression.
6) Fig 2d/e: These would benefit by inclusion of some known miR-30c targets (e.g. DLL4).
7) Fig 3f: Interesting that despite the effects of circHIPK3 inhibition on the lytic cycle, miR-27a is still down-regulated.

8) Line 225-226/Fig 5: "either circHIPK3 depletion or overexpression of a miR-30c mimic had on DLL4 expression in TREx-BCBL1-RTA cells". The reverse experiments would be invaluable, especially inhibition of the miR-30c, ideally over-expression of the circle too.
9) Fig 5g: As mentioned above in point 2. What about AGO2 association of the DDL4 3'UTR under these conditions? Or association of the miRNAs with the 3'UTR.
10) Fig 7: (See main point 3) What happens in double knockdown cells? circHIPK3 kd or miR-30c over-expression/kd should not have any effect on lytic replication and cell cycle genes in DLL4kd cells.
11) In many figures there is lack of error bars, where the control is set to 1. How is statistical significance calculated if one of the conditions has no variance? What statistical tests are used?

Referee #3:

Herpes viruses are known to subvert cellular pathways to facilitate their persistence in the infected host. This article by Harper et al. presents another example of how KSHV can manipulate a cellular non-coding RNA regulatory network, an emerging field of cellular homeostasis, to facilitate its replication upon lytic reactivation. The manuscript is well evidenced in terms of functional experiments that support their hypothesis; however, details of mechanistic insights are not well experimented, which is not also the main focus of EMBO reports. Major and minor comments that would improve the quality of the manuscript are described below.

Major comments:
1. The manuscript would greatly benefit from text editing. Breaking down longer than average sentences into multiple short sentences will improve readability of the manuscript and provide easy flow of the results being discussed. Conclusions are being presented ahead of the experiments in multiple occasions in the manuscript leading to redundancy and confusion without reading the whole manuscript. Figure legends indicate the number of experiments as N=X, the authors should indicate whether these are independent experiments or replicates of a single experiment. The authors should also consider condensing the number of supplementary Figures by combining those with similar messages (example, Fig S1-3, Fig S5-7and Fig S8-9 can be combined into individual figures reduce the 9 supplementary figures into only 4 that can go along with the main figures).
2. For a circular RNA to serve a sponging function to a particular miRNA, it needs to harbor multiple binding sites. Whereas, for target mediated degradation of an miRNA to happen there has to be extensive pairing of target RNA and miRNA or high 3' complimentary between the particular miRNA and its target, which does not seem to be the case for circHIPK3/miR-29b or circHIPK3/miR-30c interaction. A sponging function does not necessary lead to the degradation of either the miRNA or the circRNA. The authors should discuss these two concepts in the text with examples from the literature and provide experimental evidence on how circHIPK3 would lead to miR-30c degradation without itself being degraded by the miRNA machinery?
2. Figure 1: Does the down regulation of miR-30c and miR-29b during KSHV lytic replication happen in other PEL cells other than TREx-BCBL1-RTA?
3. Line 123-127: The authors should explain how they can differentiate between mature, pri- and pre-mRNA of miR-29b and miR-30c in their qPCR quantification. This has been shown neither in the results nor in the methods section. A control miRNA that is not affected by KSHV reactivation in their assay would demonstrate that this is ended specific to miR-29b and miR-30c.
4. The statement on the conclusion (Line 127): "potentially implicating ncRNA network regulation" is not founded with experimental results at this point, it can be re-stated as a hypothesis instead or removed all together as this it is being tested later starting from line 146.
5. Figure 1J and else where, it is curious why the authors chose to measure infectivity by quantitating a single viral transcript (ORF 57) instead of other robust methods such as using LANA IFA on re-infected cells.
6. Line 149: Please indicate the specific RNA binding prediction tool used and (Figure S4) please specify the total number of predicted binding sites for each miRNA on circHIPK3 in the text.
7. Figure 2A and B would benefit from a schematic depicting the HIPK3 circRNA relative to its linear transcript and primer binding sites used to analyze circular and linear HIPK3 RNA by qPCR. What kind of quantitative method was used (TaqMan, SYBR Green, or something else)?
8. Figure 2C and lines 158-160, did the authors tested the specificity of the FISH probe using a cell that does not express circHIPK3?
9. Figure 2D does not show the levels of circHIPK3 bound to the scrambled control as stated in line 167-170, it instead compares enrichment of circHIPK3 against GAPDH which will not support the conclusion being made.
10. Figure 4: Does ORF 57 overexpression also increase the linear/pre-mRNA for HIPK3? Or is it restricted to the circular form? Would it be possible for the authors to knockdown ORF 57 in the TRx-BCBL1 cells to see if this would abrogate circHIPK3 levels and consequently increase miR-29b and miR-30c levels during KSHV lytic replication? This would show whether ORF 57 is solely responsible for the increase in circHIPK3 levels or if there are other viral factors.
11. Figure 6a and line 235: Were the shRNAs used against DLL? If yes, why do we see KD only after 24 hours of KSHV induction but not at 0 hours? When was the knockdown performed in reference to induction of lytic replication? These points should be clarified in the manuscript.
12. Figure 7G: Why do the authors use Pol II IFA, which can stain both cellular and viral replication foci and therefore results can not be attributed to only virus replication, instead of using KSHV DNA FISH to specifically stain viral replication factories? Alternatively, as it does not add any significant information to the conclusions being made, this data can be removed from the

manuscipt.

Minor comments:
1. Line 109: identify instead of "identified"
2. Please use clear labeling and statistical analysis on supplemental figures similar to the main figures.
3.Line 120: please indicate the specific microarray dataset used in the text.
4. Line 156: the statement "Given the potential of circHIPK3 to function as a miR-29b and miR-30c sponge during KSHV lytic replication" is repetitive (see line 151) and ahead of the experimental proof.
5. Figure 5F: To be consistent, please use the same order and color for bar graphs of control vs. knockdown experiments with the rest of the panels in the figure.
6. Figure 8 is referenced nowhere in the manuscript.

Referee #1

1. Fig. 1G, H: GAPDH is a target of host shutoff, so this is not the best loading control for immunoblots. Fig. 3C: Why is GAPDH reduced in Fig. 1G,H in induced conditions, but not in induced conditions in Fig. 3C?

We agree with the reviewer that GAPDH is a target of KSHV-mediated host cell shut-off and levels can decrease with high virus levels after virus reactivation. However, it is still widely used as standard loading control in KSHV biology (eg Gottwein *et al.* 2019 Cell Reports). Variation in the levels of KSHV-mediated host cell shutoff is always observed due to the chemical induction process and reactivation levels of the TREX cells. To highlight that GAPDH is a suitable loading protein, we have re-run Fig 3C samples using Tubulin. This highlights consistency with the GAPDH below (not included in the revised manuscript).

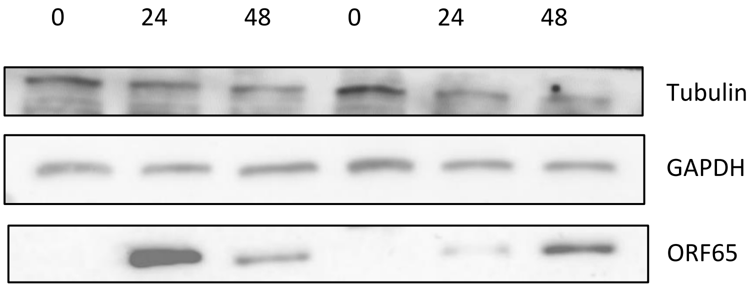

2. While authors performed RIP on ORF57 and an ORF57-RGG (Fig S6) mutant showing that the RNA binding mutant did not bind transcripts, authors did not show whether transfection of ORF57-RGG mutant still results in circHIPK3 increased expression. This is a critical experiment to support their conclusion that ORF57 enhances circHIPK3 expression.
Transfections of RGG1/2 in 293Ts have been included with levels of circHIPK3 and HIPK3 analysed via qPCR. We see a significant decrease in circHIPK3 levels in the RGG mutant transfections compared to WT. However, we still see a slight upregulation compared to GFP or GFP-ORF50, indicative of involvement of additional factors. Lines 214-219, pg 8; Fig 4c-4d.

3. Authors need to expand on details of analysis and results for miRNA-Seq dataset. The methods section was sparse and SRA/GEO accession numbers were not provided. Additional figures should be generated to support their claim at line 113 "with the vast majority downregulated during lytic replication in B cells."
We have now included a new section in Methods "Bioinformatics analysis of sequencing datasets" to highlight the data analysis text. This section combines both the miRNA-Seq and mRNA-Seq analysis. We have also included a bar plot of the count of downregulated versus upregulated miRNAs, (Fig. S1a, line 111, pg5).

4. Fig. 2C: Labels are needed on the figure. FISH typically is not used for BSJs because a single probe is usually not sensitive enough. Please include negative controls and show fields of cells, not a single cell.
FISH images now include a field of cells (Fig 2C), although, utilisation of Airy Scan limited the field of view to a group of cells. FISH was performed using the Thermo Fisher ViewCell RNA kit which uses further steps to amplify weaker signals as detailed in the Methods section. To ensure specificity of the probe, FISH was also performed in circHIPK3 KD cell lines with results showing that the signal was not strong enough to be visualised. However, we have not included this result in the manuscript.

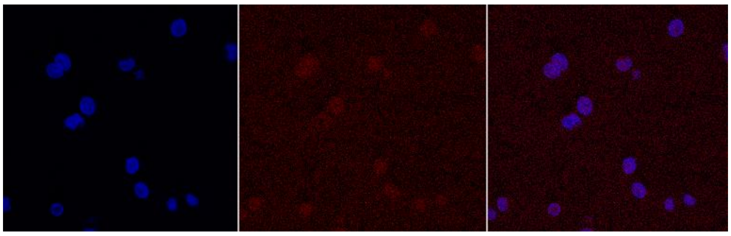

5. Fig. 4: Showing the circHIPK3 to linear HIPK3 ratios would be helpful to assess how these ratios might be changing at these different timepoints.
qPCR of linear HIPK3 levels at 0-72 hrs have now been included. Line 202-203, pg 8; Fig 4B.

6. In conventional ceRNA theory, miRNAs will bind to ceRNAs but not actively degraded. How do you explain there is a reduction of miR-30 with upregulated circHIPK3?
We have now discussed these concepts in the discussion section. Lines 369-387, pgs 13-14.

7. Current evidence is not enough to claim ORF57's function in promoting circularization. Do you see the reduction of linear HIPK3 upon overexpression of circHIPK3?
We assume the reviewer is asking whether linear HIPK3 levels reduce upon ORF57 expression. New results show that upon WT ORF57 transfection into 293Ts, we observe a small reduction in linear HIPK3 levels, however no reduction is observed in the presence of the RGG mutant (Lines 216-219, pg8; Fig 4D).

8. DLL4 is just one of miR-30's targets. What about other targets? Since you have done RNA-Seq (Fig. S8), you should be able to check expression changes of other targets. For example, you can separate mRNAs with miR-30 seed match and mRNAs without, and perform a Kolmogorov-Smirnov test.
We thank the reviewer for the suggestion regarding miR-30 dependent changes in mRNA expression. We extracted known miR-30-mRNA interactions from the miRTarBase database and separated the mRNA expression values during KSHV replication at 20 hours into 1) mRNAs targeted by miR-30c above and 2) the rest and compared their distribution with the Kolmogorov-Smirnov test. We observed a significant increase in the expression of these mRNAs (Line 240-242, pg 9; Supp Fig 4B), which is consistent with the idea that miR-30 is important in KSHV lytic replication.

Referee #2

1) The authors propose a ceRNA network, which would be more accurately described as a circular RNA acting as a sponge. A critical point around any such network is the question of abundance (Denzler et al., Molecular Cell, 2014, PMID: 24793693). Is it possible circHIPK3 having 3 and 2 sites for miR-29b and miR-30c sequesters enough of the two miRNAs to de-repress DLL4 expression? The authors should consider this and determine absolute copy number of circHIPK3, miR-29b, miR-30c, DDL4 mRNA in latent and lytic cells. This would substantially strengthen the evidence supporting the existence of the proposed network.
We used a standard curve method to show final copy number estimates of circHIPK3, miRNAs and DLL4 per uL. This has shown that circHIPK3 is a highly abundant circRNA. This is further validated by its consistently low CT values in qPCR and in the literature where it has previously been identified as highly expressed (Zheng *et al*. 2016. Nat Comms Circular RNA profiling reveals an abundant circHIPK3 that regulates cell growth by sponging multiple miRNAs). This is included in Sup Fig5A-C. Lines 271-274, pg 10).

2) Experimentally, the authors use extensively over-expression of miR-29b and miR-30c. These results in non-physiologically relevant copy numbers of the two mature miRNAs. Experiments where

the two miRNAs are inhibited should be performed to confirm that the effects are observed with the endogenous miRNAs.
Experiments have been performed using a miR-30c antagomiR, looking at RNA and protein levels of DLL4, which showed a small increase (Lines 255-256, pg 9; Fig 5I-J). Additionally ORF65 levels were investigated to show the antagomiR had no inhibitory effect on the virus (Lines 142-144, pg 6; Supp Fig 1K). Finally the antagomiR was used in both Ago2 RIPs (Line 170, pg 7; Fig 2D) and the DLL4 3'UTR luciferase experiments (Lines 259-261, pg 9; Fig 5L).

3) A critical experiment that would validate the existence of the circHIPK3/miR-30c/DDL4 network is missing: the authors should test whether circHIPK3 knockdown or miR-30c over-expression/knockdown have any effects on lytic replication and cell cycle genes in DLL4knockdown cells. I appreciate these are complex experiments, but they would demonstrate that circHIPK3 and miR-30c exert their effects on the lytic through DDL4.
We have performed 2 sets of double KD experiments with either circHIPK3 + DLL4 KD or with miR-30c O/E and DLL4 KD (Lines 287-293, pg10-11; Supp Fig 6). The dual KD led to no significant changes in cell cycle genes compared to the single KDs. The effect on the virus was also examined using ORF65 and no significant change was observed.

4) All experiments in the manuscript are performed in one KSHV-infected B cell line. It would be useful to validate some of the key findings in another KSHV-positive PEL cell line. For example, regulation of miR-30c and circHIPK3 and the effect of their manipulation on the lytic switch.
We have looked at the levels of circHIPK3 and DLL4 in BC-3 cells (another KSHV PEL line) and HEK-293T-rKSHV.219. Results in all cell lines show a similar trend, with an upregulation of circHIPK3 and DLL4. We also looked at miR-30c levels in HEK-293T-rKSHV.219 which were reduced (Lines 275-280 , pg10; Supp Fig 5D-H).

5) The section on the role of ORF57 in circHIPK3 induction is interesting but incomplete. Do the authors imply that ORF57 contributes to generation (back-splicing) or stability of the circRNA? This needs to be further investigated or removed.
The potential role of ORF57 has been further expanded on in the discussion (lines 403-419, pgs 14-15). Additional experiments have also been included utilising the RGG mutant (lines 214-219, pg 8; Fig 4C/D).

6) A section on statistics should be added to materials and methods.
We now include a statistical analysis section in the updated manuscript (lines 659-663, pg 23) as well explicitly stating the tests in the figure legends.

Minor points:
1) Line 62-63: Please add some references on ceRNAs.
References added.

2) Line 114-115/Fig 1a: Please explain more what you mean by "Based on a combination of known function, abundance and Z-score, miR-30c and miR-29b were selected for further investigation". There is a long list of miRNAs in Fig1A.
The manuscript now explains in greater detail the selection process behind miRNAs (Lines 113-121; pg 5). We have also now included in supplementary the online datasets of other miRNAs that were dysregulated in our Seq but not in other public datasets, and therefore we focused on 30c and 29b (Supp Fig 1d-f).

3) Line 117/Fig 1b/c: interesting to see this miRNA regulation in KS biopsies. However, these are predominantly comprised of latently infected cells. How does this fit with the lytic switch?

It is a common misconception that KS only contains latent cells. Both latent and lytic replicating cells are essential for KSHV-mediated tumourigenesis. Lytic replication has been shown to be required to sustain the population of latently-infected tumour cells that would otherwise be reduced due to the poor persistence of the KSHV episome during tumour cell division (Grundhoff, A. & Ganem, D. J Clin Invest 113, 124-36 (2004)). Lytically-expressed proteins also mediate paracrine secretion of growth and angiogenic factors, essential for tumour development (Nicholas, J. Front Biosci 12, 265-81 (2007), and induce DNA damage and genomic instability (Jackson, B. et al. PLoS Path 10, e1004098 (2014). It should also be noted that the miR-Seq analysed is compared to adjacent non-KSHV infected cells which should not have any dysregulation of the miRNAs, allowing any lytic KS changes to be noted.

4) Line 149: How were the RNA-binding predictions performed? Was it just alignment?
This has now been added in text, RNA-binding predictions were performed with specialist software for identifying interactions between miRNAs and longer RNAs, we used 2 separate programmes: University of Freiburg IntaRNA – RNA-RNA interaction programme and University of Bielefeld (BiBiServ) RNAhybrid program. Both programmes use alignments, free energy predictions and other key aspects for alignments (Lines 152-156, pg 6; Supp Fig 2A).

5) Fig 2d: This is a prime example where inhibition of miRNAs should be considered in addition to over-expression.
See above review 2, main point 2. The Ago RIP with antagomiR has now been included (Line 170, pg 7; Fig 2D).

6) Fig 2d/e: These would benefit by inclusion of some known miR-30c targets (e.g. DLL4).
We have added a figure showing enrichment of DLL4 in the AGO2 RIP. Importantly we show that transfection of the miR-30c antagomiR led to reduced association of DLL4. This indicates that when miR-30c levels are reduced, DLL4 as a putative target associates less with the miRNA machinery (Lines 256-261, pg 9; Fig 5K). Additionally, experiments showing enrichment of DLL4 in miR-30c pulldowns have also been included (Line 249-251, pg9, Fig5G).

7) Fig 3f: Interesting that despite the effects of circHIPK3 inhibition on the lytic cycle, miR-27a is still down-regulated.
We believe this is because miR-27a is downregulated very early in viral replication (strongly downregulated at 16hrs on the miR-Seq), whereas circHIPK3 KD starts to affect the virus from 24hrs onwards in the lytic cascade, for instance we see circHIPK3 KD reduces the late protein ORF65 but not the early protein ORF57.

8) Line 225-226/Fig 5: "either circHIPK3 depletion or overexpression of a miR-30c mimic had on DLL4 expression in TREx-BCBL1-RTA cells". The reverse experiments would be invaluable, especially inhibition of the miR-30c, ideally over-expression of the circle too.
Unfortunately overexpressing circRNAs is tricky, with overexpression of the linear form usually occurring too due to inefficient circularisation and thereby you can't attribute which functions are a result of the linear or the circular form. To circumvent this issue, we have looked at DLL4 levels at both protein and RNA levels in cells where miR-30c is inhibited, which results in a small increase in DLL4 (Lines 255-257, pg 9; Fig 5I+J).

9) Fig 5g: As mentioned above in point 2. What about AGO2 association of the DDL4 3'UTR under these conditions? Or association of the miRNAs with the 3'UTR.
We have examined DLL4 association with Ago2 in miR-30c antagomiR transfected cells (Lines 255-259, pg 9; Fig 5K). Results show a decreased association with Ago2 when miR-30c is inhibited. In

addition, we have performed the 3'UTR luciferase assay in antagomiR transfected cells (Lines 259-261, pg 9; Fig 5L), showing a slight increase in luminescence.

10) Fig 7: (See main point 3) What happens in double knockdown cells? circHIPK3 kd or miR-30c over-expression/kd should not have any effect on lytic replication and cell cycle genes in DLL4kd cells.
See response to Reviewer 2, Main point 3.

11) In many figures there is lack of error bars, where the control is set to 1. How is statistical significance calculated if one of the conditions has no variance? What statistical tests are used?
It is common practice to perform statistics for qPCR on the delta-delta CT values before calculating and graphing the fold changes (Livak and Schmittgen. Methods 25, 402-8; 2001). Thus, both the target and control groups have variances for statistical significance.

Referee #3:

1. The manuscript would greatly benefit from text editing. Breaking down longer than average sentences into multiple short sentences will improve readability of the manuscript and provide easy flow of the results being discussed. Conclusions are being presented ahead of the experiments in multiple occasions in the manuscript leading to redundancy and confusion without reading the whole manuscript. Figure legends indicate the number of experiments as N=X, the authors should indicate whether these are independent experiments or replicates of a single experiment. The authors should also consider condensing the number of supplementary Figures by combining those with similar messages (example, Fig S1-3, Fig S5-7and Fig S8-9 can be combined into individual figures reduce the 9 supplementary figures into only 4 that can go along with the main figures).
The manuscript has been modified to be more readable, with premature conclusions removed throughout and extra text breaks added. Supplementary figures have now been modified and combined. Figure legends now clarify whether n=x are biological repeats or experimental.

2. For a circular RNA to serve a sponging function to a particular miRNA, it needs to harbor multiple binding sites. Whereas, for target mediated degradation of an miRNA to happen there has to be extensive pairing of target RNA and miRNA or high 3' complimentary between the particular miRNA and its target, which does not seem to be the case for circHIPK3/miR-29b or circHIPK3/miR-30c interaction. A sponging function does not necessary lead to the degradation of either the miRNA or the circRNA. The authors should discuss these two concepts in the text with examples from the literature and provide experimental evidence on how circHIPK3 would lead to miR-30c degradation without itself being degraded by the miRNA machinery?
We have now discussed these concepts in the discussion (Lines 269-287; pg 13-14). However, we feel that to fully investigation the TDMD mechanism it is outside the scope of the paper and not feasible in 3 month response.

2. Figure 1: Does the down regulation of miR-30c and miR-29b during KSHV lytic replication happen in other PEL cells other than TREx-BCBL1-RTA?
See response to Reviewer 2, Main point 4.

3. Line 123-127: The authors should explain how they can differentiate between mature, pri- and pre-mRNA of miR-29b and miR-30c in their qPCR quantification. This has been shown neither in the results nor in the methods section. A control miRNA that is not affected by KSHV reactivation in their assay would demonstrate that this is ended specific to miR-29b and miR-30c.
Apologies for the omission, we have amended Fig1E+F, with primers for either: the primary alone, the primary and pre combined and the mature Details of primers are in Supp Table 1. Primary alone

used primers for a sequence only found in the pri-transcript. Pri+pre was for a sequence not found in the mature. Mature levels were measured using the qiagen miScript kit which is specific for mature only. A control miRNA of miR-29b-3p has been included in supplementary with levels not affected by KSHV lytic replication. (Supp 1H lines 124-125, pg 5).

4. The statement on the conclusion (Line 127): "potentially implicating ncRNA network regulation" is not founded with experimental results at this point, it can be re-stated as a hypothesis instead or removed all together as this it is being tested later starting from line 146.
We have re-stated the sentence as a hypothesis, line 126-128.

5. Figure 1J and else where, it is curious why the authors chose to measure infectivity by quantitating a single viral transcript (ORF 57) instead of other robust methods such as using LANA IFA on re-infected cells.
Reinfection assays are a validated assay to confirm successful production of infectious virions using naïve 293 cells followed by measuring lytic gene expression. Similar assays have been performed in many papers (eg Schumann et al Nature Microbiology, 2:16201), including papers that initially characterised whether a cell type was permissible (Limited Transmission of Kaposi's Sarcoma-Associated Herpesvirus in Cultured Cells, 1998), (The restricted cellular host range of human herpesvirus 8, 2000).

6. Line 149: Please indicate the specific RNA binding prediction tool used and (Figure S4) please specify the total number of predicted binding sites for each miRNA on circHIPK3 in the text.
We have now stated this information in the text, Lines 152-156, pg6.

7. Figure 2A and B would benefit from a schematic depicting the HIPK3 circRNA relative to its linear transcript and primer binding sites used to analyze circular and linear HIPK3 RNA by qPCR. What kind of quantitative method was used (TaqMan, SYBR Green, or something else)?
Our qPCR analysis utilised Promega GoTaq. This is a master mix containing BRYT Green dye, a fluorescent DNA binding green dye similar to SYBR Green. A schematic has now been added highlighting the primer binding sites and the direction of the primers (convergent vs divergent), Lines 157-158, pg6; Supp Fig 2B).

8. Figure 2C and lines 158-160, did the authors tested the specificity of the FISH probe using a cell that does not express circHIPK3?
Please see response to Review 1, main point 4.

9. Figure 2D does not show the levels of circHIPK3 bound to the scrambled control as stated in line 167-170, it instead compares enrichment of circHIPK3 against GAPDH which will not support the conclusion being made.
We agree the wording of this was unclear, the Ago2 RIP was performed with a dual normalisation. To account between different repeats, fold enrichment was used, analysing circHIPK3 over GAPDH, which represented the 'background'. For each repeat this was value was then normalised as fold change relative to the circHIPK3/GAPDH enrichment of the IgG control, which again should represent 'background'. We have clarified this in the manuscript. Through this analysis, circHIPK3 was bound higher than the background GAPDH in Ago2 RIPs, suggesting association. This increased association was seen with transfection of the miRNAs and reduced with the antagomiR (Lines 164-168, pg 6-7).

10. Figure 4: Does ORF 57 overexpression also increase the linear/pre-mRNA for HIPK3? Or is it restricted to the circular form? Would it be possible for the authors to knockdown ORF 57 in the TRx-BCBL1 cells to see if this would abrogate circHIPK3 levels and consequently increase miR-29b

and miR-30c levels during KSHV lytic replication? This would show whether ORF 57 is solely responsible for the increase in circHIPK3 levels or if there are other viral factors.
We agree this would be a nice experiment to perform however KD of ORF57 is technically challenging, KO of ORF57 has been shown to lead to failure of lytic replication as shown by BeltCappellino *et al* 2019 (CRISPR/Cas9-Mediated Knockout and *In Situ* Inversion of the ORF57 Gene from All Copies of the Kaposi's Sarcoma-Associated Herpesvirus Genome in BCBL-1 Cells). Furthermore it takes months to achieve due to the multiple KSHV genomes in each cell requiring editing, necessitating single cell monoclonal KOs. Additionally as ORF57 is an integral early protein in the lytic cascade, KD of ORF57 also prevents all downstream KSHV protein expression, therefore you cannot identify if any effects are due to ORF57 specifically or due inhibition of other viral factors. Instead, to circumvent the ORF57 KD issues, we have performed transfection of ORF57 into 293Ts to look at linear HIPK3, which show a small decrease, notably we didn't see this decrease with transfection of the ORF57 RGG1/2 mutant (Lines 207-210, pg 8; Fig 4D).

11. Figure 6a and line 235: Were the shRNAs used against DLL? If yes, why do we see KD only after 24 hours of KSHV induction but not at 0 hours? When was the knockdown performed in reference to induction of lytic replication? These points should be clarified in the manuscript.
Stable KDs using lentiviruses expressing shRNA were performed at least 2 weeks prior to all experiments and is now clarified in the manuscript (Lines 264-265, pg10). KD is present at 0 hrs, however, due to the upregulation at 24 hrs in scr it is more noticeable.

12. Figure 7G: Why do the authors use Pol II IFA, which can stain both cellular and viral replication foci and therefore results can not be attributed to only virus replication, instead of using KSHV DNA FISH to specifically stain viral replication factories? Alternatively, as it does not add any significant information to the conclusions being made, this data can be removed from the manuscipt.
We have removed it from the manuscript

Minor comments:
1. Line 109: identify instead of "identified"
Changed

2. Please use clear labeling and statistical analysis on supplemental figures similar to the main figures.
Corrected

3.Line 120: please indicate the specific microarray dataset used in the text.
Now included

4. Line 156: the statement "Given the potential of circHIPK3 to function as a miR-29b and miR-30c sponge during KSHV lytic replication" is repetitive (see line 151) and ahead of the experimental proof.
Deleted

5. Figure 5F: To be consistent, please use the same order and color for bar graphs of control vs. knockdown experiments with the rest of the panels in the figure.
Changed

6. Figure 8 is referenced nowhere in the manuscript.
Now referred to in the text (Line 334, pg 12).

1st Revision - Editorial Decision **28th Jan 2022**

Dear Prof. Whitehouse,

Thank you for the submission of your revised manuscript to our editorial offices. I have now received the reports from the three referees that were asked to re-evaluate your study, you will find below. As you will see, the referees support publication of the revised manuscript. Referee #2 and #3 have further suggestions to improve the study I ask you to address in a final revised version of the manuscript. Please also provide a point-by-point-response addressing these remaining points.

Moreover, I have these editorial requests:

- I would suggest this modified title:
Dysregulation of the miR-30c/DLL4 axis by circHIPK3 is essential for KSHV lytic replication

- Please provide the abstract written in present tense throughout.

- It seems authors Euan McDonnell and Andrew Macdonald are missing from the author contributions. Please check.

- We updated our journal's competing interests policy in January 2022 and request authors to consider both actual and perceived competing interests. Please review the policy https://www.embopress.org/competing-interests and add a statement declaring your competing interests. Please name that section 'Disclosure and Competing Interests Statement' and add it after the acknowledgements section.

- Please make sure that the number "n" for how many independent experiments were performed, their nature (biological versus technical replicates), the bars and error bars (e.g. SEM, SD) and the test used to calculate p-values is indicated in the respective figure legends (main and Appendix figures), and that statistical testing has been done where applicable. Please avoid phrases like 'independent experiment', but clearly state if these were biological or technical replicates. Please add complete statistical testing to all diagrams. Please also indicate (e.g. with n.s.) if testing was performed, but the differences are not significant.

- Please provide the Appendix as pdf file with page numbers, a table of contents (including page numbers) and titles and legends for each figure and table. Thus, please move these legends from the main manuscript text to the Appendix file. Please put each legend below the corresponding figure/table. Please name the figures 'Appendix Figure Sx' and the tables 'Appendix Table Sx' in the Appendix file and change the callouts for these accordingly. Finally, please remove the Appendix legends from the manuscript text file.

- Please add the title 'Figure Legends' to the figure legends in the main manuscript text.

- Supplementary Figure 1 (that should be named 'Appendix Figure S1') has no panel G (but panels H-K). Please check and name the panels sequentially and alphabetically.

- It seems there are no separate callouts for the panels of Appendix Fig S7. Please add these.

- Please add a link for direct access to the deposited dataset to the Data Availability section and make sure the data is public upon publication of the paper. Please also update field F18 of the author checklist accordingly.

- For microscopic images, please add scale bars of similar style and thickness to all the microscopic images, using clearly visible black or white bars (depending on the background). Presently, many scale bars are too thin. Please do not write on or near the bars in the image but define the size in the respective figure legend.

- Please also note our reference format (and change the references accordingly):
http://www.embopress.org/page/journal/14693178/authorguide#referencesformat

- Finally, please find attached a word file of the manuscript text (provided by our publisher) with a few changes and queries we ask you to include in your final manuscript text. Please provide your final manuscript file with track changes, in order that we can see any modifications done.

In addition, I would need from you:
- a short, two-sentence summary of the manuscript (not more than 35 words).
- two to four short bullet points (2 lines) highlighting the key findings of your study.
- a schematic summary figure (in jpeg or tiff format with the exact width of 550 pixels and a height of not more than 400 pixels) that can be used as a visual synopsis on our website.

I look forward to seeing the final revised version of your manuscript when it is ready. Please let me know if you have questions regarding the revision.

Please use this link to submit your revision: https://embor.msubmit.net/cgi-bin/main.plex

Yours sincerely,

Achim Breiling
Editor
EMBO Reports

Referee #1:

The authors have included new experiments, analysis, and descriptions of the methods used in this manuscript. While some questions remain, no manuscript can answer every question in great detail. The reviewers have sufficiently addressed my previous concerns.

Referee #2:

The authors have made suitable efforts to address my points. However, there are still some issues which make me have reservations before I recommend this manuscript for publication.
1) The authors claim that "disruption at any point within this novel ncRNA regulatory network has a detrimental effect on KSHV lytic replication". However, inhibition of miR-30c does not have any effect on lytic reactivation (or at least ORF65 levels), despite affecting DLL4. How can circHIPK3 sequestering of miR-30c affect the lytic cycle but sequestering miR-30c with an antagomir does not? The main findings of the manuscript are really interesting but this weakness suggests that sequestering miR-30c is, at best, only partly the mechanism employed by circHIPK3 to regulate the lytic switch. My suggestion would be for the authors to include an honest evaluation of this key limitation in their Discussion and tone down the statement about "disruption at any point...".
2) The authors determined copy numbers as suggested. I am unclear why there are no error bars in the relevant figure (Fig S5) and what "Copy number estimates per µL of 1µg total cDNA from TREx-BCBL-1-Rta cells" means. Commonly absolute abundance of transcripts should be expressed per cell or an amount of total RNA.
3) I want to thank the authors for testing more cell lines. However, they should add a figure showing miR-30c levels in BC3 cells upon induction of the lytic cycle.
4) I made a minor point about KS biopsies. I would like to reassure the authors that this was not based on a misconception (I used "predominantly comprised..."). I am not convinced that the results presented in the manuscript can be related to KS lesions compared to adjacent tissues, which are most likely comprised of different cell types. In any case comparison of KS biopsies to normal tissues cannot be used to address regulators of the lytic cycle (i.e. comparing latent to lytic cells). Furthermore, as the authors point out the role of DLL4 in endothelial cells is very different to that in B lymphocytes and there is enough literature indicating that it cannot be assumed that the lytic switch operates through identical mechanisms in B cells and endothelia cells. Overall, the reference to miRNA levels in KS should be removed as it is not helpful nor meaningful.
5) Please add the authors response to my point number 11 (about statistics in qPCR) to the Methods section.

Referee #3:

The authors have addressed our concerns. One minor correction is needed on page 17, line 481 that should be deleted or modified since this figure was deleted from the manuscript (response to review comment #12).

Dear Dr Breiling

We thank the reviewers for their positive additional comments to which we provide a point-by-point response below. Changes are highlighted in the marked up version of the revised manuscript.

We hope that you now deem this manuscript suitable for publication and thank you for the opportunity to resubmit this article.

Yours sincerely

Prof Adrian Whitehouse

Editorial requests:

I would suggest this modified title:
Dysregulation of the miR-30c/DLL4 axis by circHIPK3 is essential for KSHV lytic replication

Changed

- Please provide the abstract written in present tense throughout.

Changed

- It seems authors Euan McDonnell and Andrew Macdonald are missing from the author contributions. Please check.
Added

- We updated our journal's competing interests policy in January 2022 and request authors to consider both actual and perceived competing interests. Please review the policy https://www.embopress.org/competing-interests and add a statement declaring your competing interests. Please name that section 'Disclosure and Competing Interests Statement' and add it after the acknowledgements section.
Added

- Please make sure that the number "n" for how many independent experiments were performed, their nature (biological versus technical replicates), the bars and error bars (e.g. SEM, SD) and the test used to calculate p-values is indicated in the respective figure legends (main and Appendix figures), and that statistical testing has been done where applicable. Please avoid phrases like 'independent experiment', but clearly state if these were biological or technical replicates. Please add complete statistical testing to all diagrams. Please also indicate (e.g. with n.s.) if testing was performed, but the differences are not significant.
Added

- Please provide the Appendix as pdf file with page numbers, a table of contents (including page numbers) and titles and legends for each figure and table. Thus, please move these legends from the main manuscript text to the Appendix file. Please put each legend below the corresponding figure/table. Please name the figures 'Appendix Figure Sx' and the tables 'Appendix Table Sx' in the Appendix file and change the callouts for these accordingly. Finally, please remove the Appendix

legends from the manuscript text file.
Changed as instructed

- Please add the title 'Figure Legends' to the figure legends in the main manuscript text.
Added

- Supplementary Figure 1 (that should be named 'Appendix Figure S1') has no panel G (but panels H-K). Please check and name the panels sequentially and alphabetically.
Changed

- It seems there are no separate callouts for the panels of Appendix Fig S7. Please add these.
Added

- Please add a link for direct access to the deposited dataset to the Data Availability section and make sure the data is public upon publication of the paper. Please also update field F18 of the author checklist accordingly.
Added and updated

- For microscopic images, please add scale bars of similar style and thickness to all the microscopic images, using clearly visible black or white bars (depending on the background). Presently, many scale bars are too thin. Please do not write on or near the bars in the image but define the size in the respective figure legend.

Added

- Please also note our reference format (and change the references accordingly):
http://www.embopress.org/page/journal/14693178/authorguide#referencesformat

Dataset references and original research paper references changed

Referee #1:

The authors have included new experiments, analysis, and descriptions of the methods used in this manuscript. While some questions remain, no manuscript can answer every question in great detail. The reviewers have sufficiently addressed my previous concerns.

No additonal comments required

Referee #2:

The authors have made suitable efforts to address my points. However, there are still some issues which make me have reservations before I recommend this manuscript for publication.

1) The authors claim that "disruption at any point within this novel ncRNA regulatory network has a

detrimental effect on KSHV lytic replication". However, inhibition of miR-30c does not have any effect on lytic reactivation (or at least ORF65 levels), despite affecting DLL4. How can circHIPK3 sequestering of miR-30c affect the lytic cycle but sequestering miR-30c with an antagomir does not? The main findings of the manuscript are really interesting but this weakness suggests that sequestering miR-30c is, at best, only partly the mechanism employed by circHIPK3 to regulate the lytic switch. My suggestion would be for the authors to include an honest evaluation of this key limitation in their Discussion and tone down the statement about "disruption at any point...".

miR-30c is inhibitory to the virus and usually downregulated through lytic replication, thus when we transfected in an antagomiR alongside lytic replication, miR-30c levels are already significantly reduced and as such had little additional effect on the virus, in short when using the antagomiR, circHIPK3 is already sequestering the majority of miR-30c. Prevention of this sequestering of miR-30c through either circHIPK3 KDs or transfection of a miR-30C mimic however, does inhibit viral replication. Therefore these results clearly show disruption at multiple points during the ncRNA network affect KSHV lytic replication.

2) The authors determined copy numbers as suggested. I am unclear why there are no error bars in the relevant figure (Fig S5) and what "Copy number estimates per µL of 1µg total cDNA from TREx-BCBL-1-Rta cells" means. Commonly absolute abundance of transcripts should be expressed per cell or an amount of total RNA.

Error bars have now been added to copy number calculations and abundance now estimated per amount of total RNA (clarified in Appendix figure legend S5)

3) I want to thank the authors for testing more cell lines. However, they should add a figure showing miR-30c levels in BC3 cells upon induction of the lytic cycle.

This has now been added and referred to in text (Appendix S5h, pg 10, line 282)

4) I made a minor point about KS biopsies. I would like to reassure the authors that this was not based on a misconception (I used "predominantly comprised..."). I am not convinced that the results presented in the manuscript can be related to KS lesions compared to adjacent tissues, which are most likely comprised of different cell types. In any case comparison of KS biopsies to normal tissues cannot be used to address regulators of the lytic cycle (i.e. comparing latent to lytic cells). Furthermore, as the authors point out the role of DLL4 in endothelial cells is very different to that in B lymphocytes and there is enough literature indicating that it cannot be assumed that the lytic switch operates through identical mechanisms in B cells and endothelia cells. Overall, the reference to miRNA levels in KS should be removed as it is not helpful nor meaningful.

This has now been removed.

5) Please add the authors response to my point number 11 (about statistics in qPCR) to the Methods section.

This has now been added (pg 19, line 519).

Referee #3:

The authors have addressed our concerns. One minor correction is needed on page 17, line 481 that should be deleted or modified since this figure was deleted from the manuscript (response to review comment #12).

We have now removed this from the paper.

Prof. Adrian Whitehouse
University of Leeds
SMCB
Garstang Building
Leeds LS2 9JT
United Kingdom

Dear Prof. Whitehouse,

I am very pleased to accept your manuscript for publication in the next available issue of EMBO reports. Thank you for your contribution to our journal.

At the end of this email I include important information about how to proceed. Please ensure that you take the time to read the information and complete and return the necessary forms to allow us to publish your manuscript as quickly as possible.

As part of the EMBO publication's Transparent Editorial Process, EMBO reports publishes online a Review Process File to accompany accepted manuscripts. As you are aware, this File will be published in conjunction with your paper and will include the referee reports, your point-by-point response and all pertinent correspondence relating to the manuscript.

If you do NOT want this File to be published, please inform the editorial office within 2 days, if you have not done so already, otherwise the File will be published by default [contact: emboreports@embo.org]. If you do opt out, the Review Process File link will point to the following statement: "No Review Process File is available with this article, as the authors have chosen not to make the review process public in this case."

Thank you again for your contribution to EMBO reports and congratulations on a successful publication. Please consider us again in the future for your most exciting work.

Yours sincerely,

Achim Breiling
Editor
EMBO Reports

****

THINGS TO DO NOW:

Please note that you will be contacted by Wiley Author Services to complete licensing and payment information. The required 'Page Charges Authorization Form' is available here: https://www.embopress.org/pb-assets/embo-site/er_apc.pdf

You will receive proofs by e-mail approximately 2-3 weeks after all relevant files have been sent to our Production Office; you should return your corrections within 2 days of receiving the proofs.

Please inform us if there is likely to be any difficulty in reaching you at the above address at that time. Failure to meet our deadlines may result in a delay of publication, or publication without your corrections.

All further communications concerning your paper should quote reference number EMBOR-2021-54117V3 and be addressed to emboreports@wiley.com.

Should you be planning a Press Release on your article, please get in contact with emboreports@wiley.com as early as possible, in order to coordinate publication and release dates.

EMBO PRESS

YOU MUST COMPLETE ALL CELLS WITH A PINK BACKGROUND ↓

PLEASE NOTE THAT THIS CHECKLIST WILL BE PUBLISHED ALONGSIDE YOUR PAPER

Corresponding Author Name: Adrian Whitehouse and Katherine Harper
Journal Submitted to: EMBO Reports
Manuscript Number: EMBOR-2021-54117V2

Reporting Checklist For Life Sciences Articles (Rev. June 2017)

This checklist is used to ensure good reporting standards and to improve the reproducibility of published results. These guidelines are consistent with the Principles and Guidelines for Reporting Preclinical Research issued by the NIH in 2014. Please follow the journal's authorship guidelines in preparing your manuscript.

A- Figures
1. Data
The data shown in figures should satisfy the following conditions:

- the data were obtained and processed according to the field's best practice and are presented to reflect the results of the experiments in an accurate and unbiased manner.
- figure panels include only data points, measurements or observations that can be compared to each other in a scientifically meaningful way.
- graphs include clearly labeled error bars for independent experiments and sample sizes. Unless justified, error bars should not be shown for technical replicates.
- if n< 5, the individual data points from each experiment should be plotted and any statistical test employed should be justified
- Source Data should be included to report the data underlying graphs. Please follow the guidelines set out in the author ship guidelines on Data Presentation.

2. Captions

Each figure caption should contain the following information, for each panel where they are relevant:

- a specification of the experimental system investigated (eg cell line, species name).
- the assay(s) and method(s) used to carry out the reported observations and measurements
- an explicit mention of the biological and chemical entity(ies) that are being measured.
- an explicit mention of the biological and chemical entity(ies) that are altered/varied/perturbed in a controlled manner.

- the exact sample size (n) for each experimental group/condition, given as a number, not a range;
- a description of the sample collection allowing the reader to understand whether the samples represent technical or biological replicates (including how many animals, litters, cultures, etc.).
- a statement of how many times the experiment shown was independently replicated in the laboratory.
- definitions of statistical methods and measures:
  - common tests, such as t-test (please specify whether paired vs. unpaired), simple χ2 tests, Wilcoxon and Mann-Whitney tests, can be unambiguously identified by name only, but more complex techniques should be described in the methods section;
  - are tests one-sided or two-sided?
  - are there adjustments for multiple comparisons?
  - exact statistical test results, e.g., P values = x but not P values < x;
  - definition of 'center values' as median or average;
  - definition of error bars as s.d. or s.e.m.

Any descriptions too long for the figure legend should be included in the methods section and/or with the source data.

In the pink boxes below, please ensure that the answers to the following questions are reported in the manuscript itself. Every question should be answered. If the question is not relevant to your research, please write NA (non applicable). We encourage you to include a specific subsection in the methods section for statistics, reagents, animal models and human subjects.

USEFUL LINKS FOR COMPLETING THIS FORM

http://www.antibodypedia.com
http://1degreebio.org
http://www.equator-network.org/reporting-guidelines/improving-bioscience-research-repor
http://grants.nih.gov/grants/olaw/olaw.htm
http://www.mrc.ac.uk/Ourresearch/Ethicsresearchguidance/Useofanimals/index.htm
http://ClinicalTrials.gov
http://www.consort-statement.org
http://www.consort-statement.org/checklists/view/32-consort/66-title
http://www.equator-network.org/reporting-guidelines/reporting-recommendations-for-tumo
http://datadryad.org
http://figshare.com
http://www.ncbi.nlm.nih.gov/gap
http://www.ebi.ac.uk/ega
http://biomodels.net/
http://biomodels.net/miriam/
http://jjj.biochem.sun.ac.za
https://osp.od.nih.gov/biosafety-biosecurity-and-emerging-biotechnology/
http://www.selectagents.gov/

B- Statistics and general methods

Please fill out these boxes ↓ (Do not worry if you cannot see all your text once you press return)

1.a. How was the sample size chosen to ensure adequate power to detect a pre-specified effect size?	As standard in the field, when planning the experiment, 3 biological repeats were decided
1.b. For animal studies, include a statement about sample size estimate even if no statistical methods were used.	N/A
2. Describe inclusion/exclusion criteria if samples or animals were excluded from the analysis. Were the criteria pre-established?	N/A
3. Were any steps taken to minimize the effects of subjective bias when allocating animals/samples to treatment (e.g. randomization procedure)? If yes, please describe.	N/A
For animal studies, include a statement about randomization even if no randomization was used.	N/A
4.a. Were any steps taken to minimize the effects of subjective bias during group allocation or/and when assessing results (e.g. blinding of the investigator)? If yes please describe.	N/A
4.b. For animal studies, include a statement about blinding even if no blinding was done	N/A
5. For every figure, are statistical tests justified as appropriate?	Yes, for data we gathered, unpaired student T test was appropriate.
Do the data meet the assumptions of the tests (e.g., normal distribution)? Describe any methods used to assess it.	Data showed a normal distribution
Is there an estimate of variation within each group of data?	Yes, graphs include error bars showing standard deviation

Is the variance similar between the groups that are being statistically compared?	

C- Reagents

6. To show that antibodies were profiled for use in the system under study (assay and species), provide a citation, catalog number and/or clone number, supplementary information or reference to an antibody validation profile. e.g., Antibodypedia (see link list at top right), 1DegreeBio (see link list at top right).	Antibody catalogue numbers are listed in methods, page 19 under 'Plasmids and Antibodies'. In short ORF65 (CRB<br>crb2005224, 1/100), ORF57 (Santa Cruz sc-135747 1/1000), GAPDH (Proteintech 60004-1-Ig 1/5000), GFP (Proteintech 66002-1-ig 1/5000), DLL4 (Proteintech 21584-1-AP 1/200). Ago2 (abcam ab186733) was used in RIPs
7. Identify the source of cell lines and report if they were recently authenticated (e.g., by STR profiling) and tested for mycoplasma contamination.	All cell line sources are described in methods page 18, paragraph 'Cell Culture'. In short TREx-BCBL1-RTA cells (gift from Professor JU Jung, University of Southern California), HEK-293T cells (ATCC), BC-3s (ATCC), HEK-293T-rKSHV.219 (gift from Dr Jeffery Vieira, University of Washington). All cell lines were tested in lab for mycoplasma contamination.

* for all hyperlinks, please see the table at the top right of the document

D- Animal Models

8. Report species, strain, gender, age of animals and genetic modification status where applicable. Please detail housing and husbandry conditions and the source of animals.	N/A
9. For experiments involving live vertebrates, include a statement of compliance with ethical regulations and identify the committee(s) approving the experiments.	N/A
10. We recommend consulting the ARRIVE guidelines (see link list at top right) (PLoS Biol. 8(6), e1000412, 2010) to ensure that other relevant aspects of animal studies are adequately reported. See author guidelines, under 'Reporting Guidelines'. See also: NIH (see link list at top right) and MRC (see link list at top right) recommendations. Please confirm compliance.	N/A

E- Human Subjects

11. Identify the committee(s) approving the study protocol.	N/A
12. Include a statement confirming that informed consent was obtained from all subjects and that the experiments conformed to the principles set out in the WMA Declaration of Helsinki and the Department of Health and Human Services Belmont Report.	N/A
13. For publication of patient photos, include a statement confirming that consent to publish was obtained.	N/A
14. Report any restrictions on the availability (and/or on the use) of human data or samples.	N/A
15. Report the clinical trial registration number (at ClinicalTrials.gov or equivalent), where applicable.	N/A
16. For phase II and III randomized controlled trials, please refer to the CONSORT flow diagram (see link list at top right) and submit the CONSORT checklist (see link list at top right) with your submission. See author guidelines, under 'Reporting Guidelines'. Please confirm you have submitted this list.	N/A
17. For tumor marker prognostic studies, we recommend that you follow the REMARK reporting guidelines (see link list at top right). See author guidelines, under 'Reporting Guidelines'. Please confirm you have followed these guidelines.	N/A

F- Data Accessibility

18: Provide a "Data Availability" section at the end of the Materials & Methods, listing the accession codes for data generated in this study and deposited in a public database (e.g. RNA-Seq data: Gene Expression Omnibus GSE39462, Proteomics data: PRIDE PXD000208 etc.) Please refer to our author guidelines for 'Data Deposition'.<br><br>Data deposition in a public repository is mandatory for:<br>a. Protein, DNA and RNA sequences<br>b. Macromolecular structures<br>c. Crystallographic data for small molecules<br>d. Functional genomics data<br>e. Proteomics and molecular interactions	This is included in methods for the RNA-Seq on page 24 under section 'Data avaliability'. Link is https://www.ncbi.nlm.nih.gov/geo/query/acc.cgi?acc=GSE186652
19. Deposition is strongly recommended for any datasets that are central and integral to the study; please consider the journal's data policy. If no structured public repository exists for a given data type, we encourage the provision of datasets in the manuscript as a Supplementary Document (see author guidelines under 'Expanded View' or in unstructured repositories such as Dryad (see link list at top right) or Figshare (see link list at top right).	N/A
20. Access to human clinical and genomic datasets should be provided with as few restrictions as possible while respecting ethical obligations to the patients and relevant medical and legal issues. If practically possible and compatible with the individual consent agreement used in the study, such data should be deposited in one of the major public access-controlled repositories such as dbGAP (see link list at top right) or EGA (see link list at top right).	N/A
21. Computational models that are central and integral to a study should be shared without restrictions and provided in a machine-readable form. The relevant accession numbers or links should be provided. When possible, standardized format (SBML, CellML) should be used instead of scripts (e.g. MATLAB). Authors are strongly encouraged to follow the MIRIAM guidelines (see link list at top right) and deposit their model in a public database such as Biomodels (see link list at top right) or JWS Online (see link list at top right). If computer source code is provided with the paper, it should be deposited in a public repository or included in supplementary information.	N/A

G- Dual use research of concern

22. Could your study fall under dual use research restrictions? Please check biosecurity documents (see link list at top right) and list of select agents and toxins (APHIS/CDC) (see link list at top right). According to our biosecurity guidelines, provide a statement only if it could.	N/A
